# A novel peptide stapling strategy enables the retention of ring-closing amino acid side chains for the Wnt/β-catenin signalling pathway†
†Electronic supplementary information (ESI) available. See DOI: 10.1039/c7sc02420g



**DOI:** 10.1039/c7sc02420g

**Published:** 2017-08-29

**Authors:** Ye Wu, Ye-Hua Li, Xiang Li, Yan Zou, Hong-Li Liao, Lei Liu, Ye-Guang Chen, Donald Bierer, Hong-Gang Hu

**Affiliations:** a School of Pharmacy , Second Military Medical University , Shanghai 200433 , China . Email: huhonggang_fox@msn.com; b Tsinghua-Peking Center for Life Sciences , Tsinghua University , Beijing 100084 , China . Email: ygchen@mail.tsinghua.edu.cn; c Bayer AG , Department of Medicinal Chemistry , Aprather Weg 18A , Wuppertal 42096 , Germany . Email: donald.bierer1@bayer.com; d School of Pharmacy , Chengdu Medical College , Chengdu 610083 , China

## Abstract

The all-hydrocarbon peptide stapling strategy has recently been extensively explored in drug discovery. There remains the potential for improvement regarding the retention of the amino acid side chains at the stapled positions. Herein, we describe a new series of amino acids that not only contain the native side chains, but also carry the alkenyl arms that are needed for the ring-closing stapling chemistry. We incorporate the new amino acids into a β-catenin-binding domain of Axin (469–482) and develop a new category of stapled peptides with the retention of the native side chains. These stapled peptides exhibit high α-helicity, strong proteolytic stability and good cell permeability. Biochemical experiments demonstrate that these stapled peptides can activate β-catenin more efficiently than canonical stapled peptides due to the presence of extra side chains. We expect that the new side-chain-retention stapling method would expand the scope of the all-hydrocarbon stapled peptide strategy by retaining some important peripheral residues for protein–protein interactions or preserving key hydrophilic side chains to improve solubility.

## Abstract 2

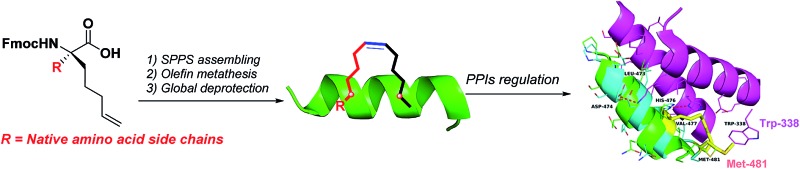
An alternative all-hydrocarbon stapling approach in which the amino acid side chains are retained at the stapled positions.

## Introduction

**Fig. 1:**
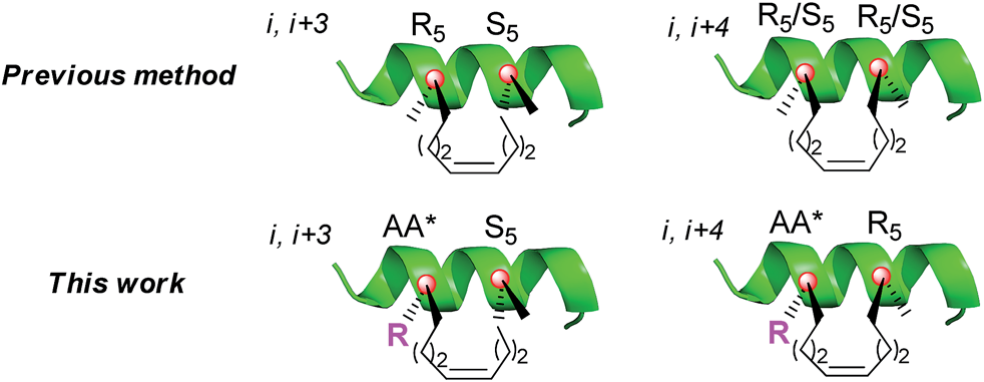
The difference between the previous method and the new method in this work. *i* = stapling position; R_5_ = Fmoc-R_5_-OH; S_5_ = Fmoc-S_5_-OH; AA* = new amino acids with modifications of the pentene groups on the α-carbon; R = amino acid side chain.

Peptide drugs offer the prospect of enhanced potency, high specificity and low toxicity because of their large interacting surfaces. These features are particularly attractive for the disruption of protein–protein interactions (PPIs). A large group of peptides bind their receptors in the α-helical conformation but the helix proportion of linear peptides is usually low in solution.^1^ The construction of peptide derivatives with a pre-organized stable α-helix topology is expected to favour receptor binding and therefore has been widely investigated in recent years.^2–4^ Generally, the stabilization of helices has been fulfilled through side-chain constraints by the covalent connection of two side-chain residues with disulfides,^5^ lactams,^6^ triazoles,^7^ and others.^8^ Among them, the all-hydrocarbon stapled peptide strategy developed by Verdine *et al.* ^9^ has been considered as one of the most promising stapling strategies for PPIs (Fig. 1). This strategy has been successfully used to improve the *in vivo* stability of peptides and enhance their membrane penetration against many biological macromolecular receptors associated with cancer, atherosclerosis, HIV and other diseases.^10–12^

To avoid the intrinsic helix-destabilizing effect of d-configured amino acids while capitalizing on the helix-stabilizing effect of α,α-disubstituted amino acids, Verdine and co-workers introduced an α-methyl group into Fmoc-R_5_-OH and Fmoc-S_5_-OH,^9^ which restricted the stapling position to Ala or mutated Ala. However, it is technically difficult to staple a peptide at the Ala position for every sequence, especially when each stapled peptide requires two stapling positions. Although reasonable stapled peptides can be rationally designed based on the Ala-scan and crystal structure of a peptide–protein complex, it remains interesting to explore whether the retention of the side chain of the stapling residue may bring about any extra benefit (Fig. 1). This may be especially important for the cases where we cannot ignore the role of peripheral residues around PPIs. Moreover, another often encountered drawback of the all-hydrocarbon stapling strategy is the solubility problem, especially in the cases where the native hydrophilic side chains of Ser, Lys or Arg are sacrificed due to the stapling.

To solve the above problems, several methods have been developed to stabilize α-helices by the replacement of an internal (*i*, *i* + 4) hydrogen bond with a covalent linkage, such as an ethylene bridge developed by Alewood *et al.*,^13^ a hydrazone bond proposed by Cabezas *et al.*,^14^ an alkyl linkage used by Arora *et al.*,^15^ a proline-derived transannular N-cap by Li *et al.*,^16^ or a pre-made diaminodiacid strategy.^17^ In this context, we are interested in developing a new alternative stapling approach in which the amino acid side chains are retained at the stapled positions.

## Results and discussion

**Fig. 2:**
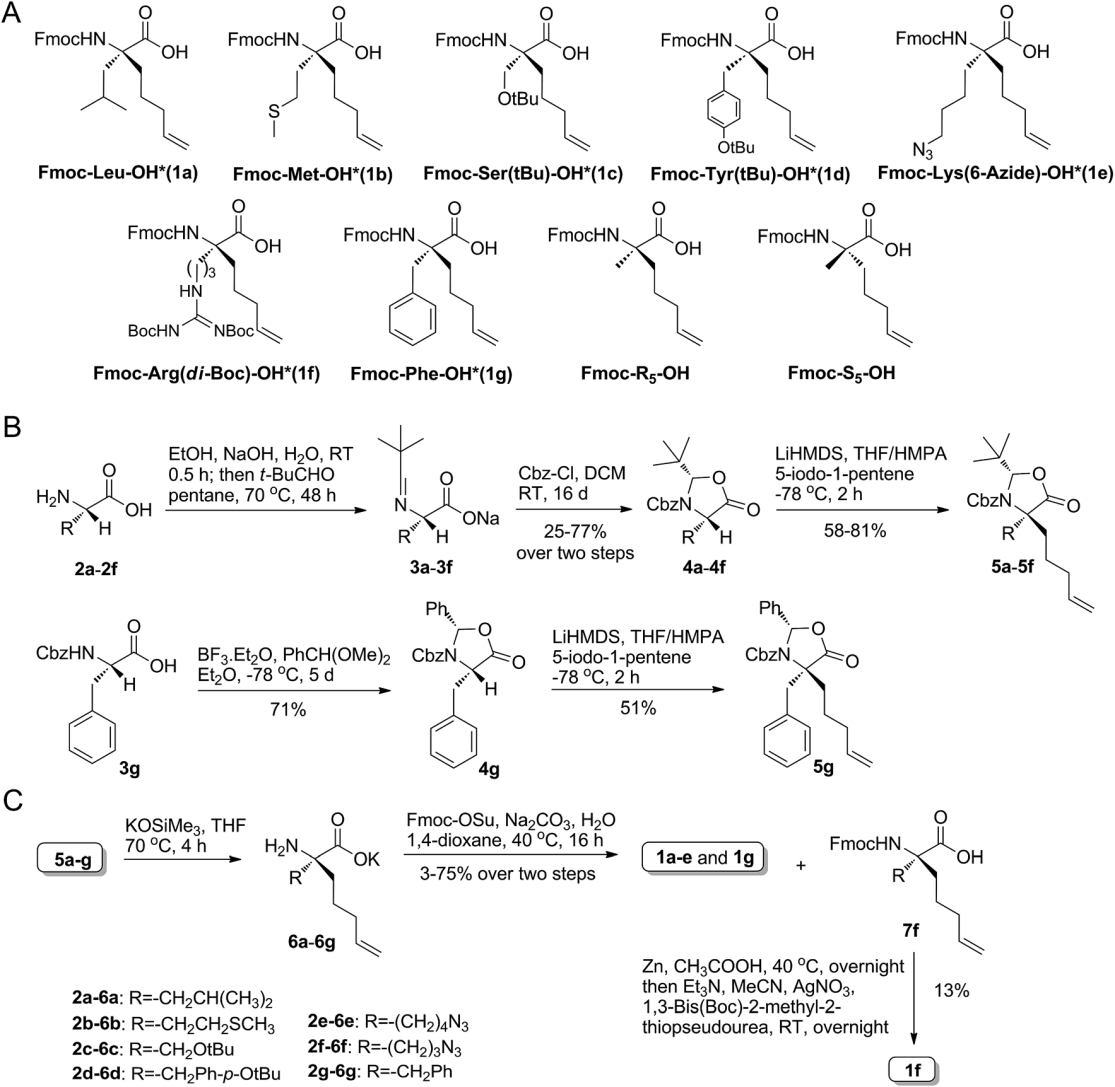
(A) The new amino acid derivatives Fmoc-AA*-OH and the commercially available Fmoc-R_5_-OH and Fmoc-S_5_-OH used in this work. (B) The synthetic routes for the key intermediates **5a–g**. (C) The synthetic route for Fmoc-AA*-OH **1a–g**. Fmoc = fluorenylmethyloxycarbonyl; *t*Bu = *tert*-butyl; Boc = *tert*-butyloxycarbonyl; Cbz = benzoxycarbonyl; RT = room temperature; DCM = dichloromethane; LiHMDS = lithium bis(trimethylsilyl)amide; HMPA = hexamethyl phosphoryl triamide; Ph = Benzene; MeCN = acetonitrile.

To achieve the above goal, we first need to make amino acids which contain both native side chains and stapling groups. For this purpose, seven classical N-Fmoc-α-pentene amino acids were tested as examples in the beginning, namely, Leu*, Met*, Ser*, Tyr*, Lys*, Arg* and Phe* (**1a–g**, as shown in Fig. 2A). A versatile *de novo* approach for the synthesis of these novel amino acids was developed by the optimization of a reported strategy for preparing Fmoc-R_8_-OH (Fig. 2B).^18*a*^

The configuration of the unnatural amino acids is critical and depends on a series of key intermediates **5a–g** (Fig. 2B), oxazolidinone-like amino acid derivatives. In our study, steric hindrance caused by the *N*-benzyl protected group and oxazolidinone was exploited to acquire the product with correct stereochemistry. First, native α-amino acids (**2a–f**), which can be obtained easily from commercial resources or the reported methods (as shown in the ESI†), were protected at the nitrogen atom to provide *N-t*-Bu imide amino acids (**3a–f**).^18*b*^ Second, **3a–f** were converted to the corresponding oxazolidinones **4a–f** by Cbz-Cl in DCM.^19^ Third, treatment of **4a–g** with LiHMDS and 5-iodo-1-pentene in a THF/HMPA solution at –78 °C afforded the desired **5a–g** in good yields. Particularly, the cyclization of **3g** with BF_3_/Et_2_O and PhCH(OMe)_2_ generated **4g** in 71% isolated yield. Hydrolysis of the alkylated oxazolidinones with KOSiMe_3_ afforded the free amino acids. Finally, the free amino groups of **6a–g** were protected by a Fmoc group to give the key amino acids **1a–e**,^20^ which were ready for use in standard Fmoc SPPS. The azide of **7f** was reduced by Zn and CH_3_COOH, and then we used AgNO_3_ to promote the guanidinylation of the amino group, yielding **1f** (Fig. 2C).^21^ To assume our novel amino acids would not racemize, we also synthesized d-configuration Phe* (**1g**), and then examined the enantiopurity of **1g**. As anticipated, our synthesis route enabled **1g** to reach 95.82% enantiopurity.

As a typical PPI model, previous studies have suggested that the Axin–β-catenin interaction is suitable for targeting by hydrocarbon-stapled peptides.^22*a*^ Herein we incorporated our novel amino acids into a β-catenin-binding domain of Axin (469–482) and presented the first stapled peptides with the retention of the native side chains. The scaffolding protein Axin, glycogen synthase kinase-3β (GSK-3β) and the adenomatous polyposis coli protein (APC) constructed a cytoplasmic protein complex, which can catalyze the phosphorylation of β-catenin in the absence of a Wnt signal.^23^ It has been demonstrated that canonical Wnt/β-catenin signaling plays a critical role in the development of embryogenesis, the maintenance of adult tissue homeostasis and the control of tissue regeneration. A variety of human diseases, including osteoporosis, neurodegenerative diseases, diabetes and Joubert syndrome, are concluded to be closely related to the aberrant activation of the Wnt/β-catenin signalling pathway.^24^

As shown in Fig. 4a, the Axin–β-catenin interface comprised both a rather hydrophobic interaction and some critical salt bridges and hydrogen bonds, of which Leu-473, Asp-474, and His-476 are critical residues and Ile-472, Val-477, Val-480 and Met-481 play moderately important roles.^22*b*^ Each of these residues is more or less involved in the binding interaction. To evaluate the new stapling strategy, we incorporated the amino acids Leu*, Met*, Ser* and Lys* combined with S_5_ or R_5_ into the β-catenin-binding domain (Axin (469–482)) (Table 1). Solid-phase peptide synthesis^25*a*^ and RCM^25*b*^ were carried out as previously reported, and thirteen stapled peptides containing Leu*/Met*/Ser*/Lys* and S_5_/R_5_ were successfully synthesized (Table 1).

**Table 1:** The synthetic route and amino acid sequences of the stapled peptides^*a*^

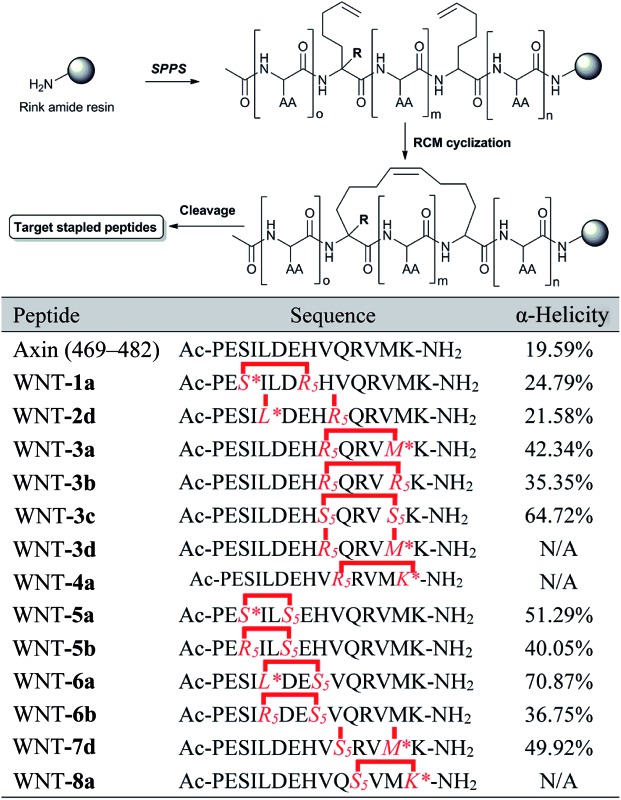

^a^*: the synthetic amino acid with the retention of the native side chain. WNT-**2d** and -**7d**: the peptide failed to undergo RCM. N/A: not applicable.

The linear peptide was first prepared using standard Fmoc SPPS procedures with Rink Amide MBHA resin as the solid support. The Fmoc protected amino acids were successively assembled onto the resin. After the peptide assembly was completed, the olefin-containing peptide was stapled using Grubb’s first-generation catalyst. The peptide was cleaved off from the resin and globally deprotected with reagent K (82.5% TFA, 5% H_2_O, 2.5% EDT, 5% thioanisole and 5% phenol). Ether precipitation gave the crude peptides, which were purified by semi-preparative RP-HPLC. WNT-**2d** and WNT-**7d** failed to undergo RCM, even under high temperature, suggesting that steric hindrance of these two sequences can result in non-productive conformations for the cyclization step.^8*d*^ The amino acid side chain of Lys* was initially an azide group, which was reduced to an amine on the resin by a nickel catalyst in the presence of NaBH_4_ after RCM (ESI, 3.1†). To test the coupling efficiency of our unnatural amino acids, we monitored the coupling step of Met* and the subsequent elongation of Val in WNT-**3a** by analytical HPLC through micro-cleavage. According to the trace comparison of each intermediate in HPLC (Fig. S1†), the coupling efficiency was not obviously compromised in spite of the introduction of an extra long alkene linker.

**Fig. 3:**
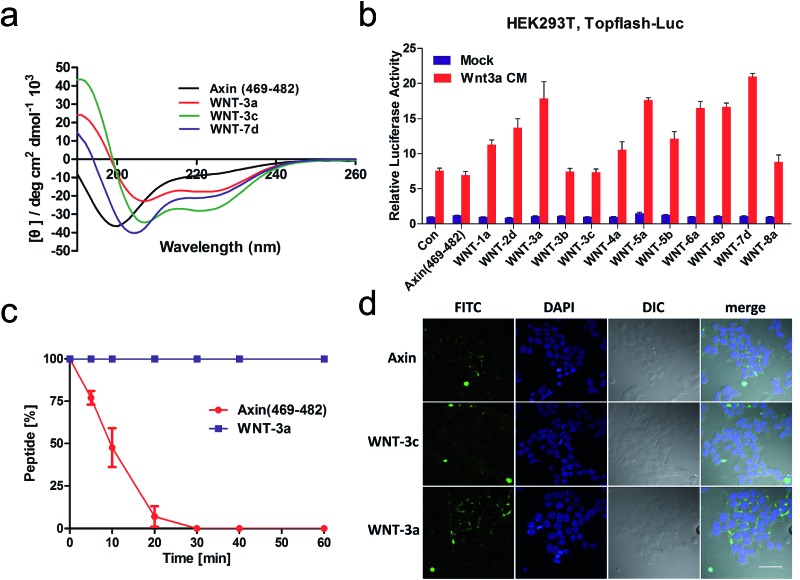
(a) CD spectra of the stapled peptides. The peptides were dissolved in PBS buffer at a final concentration of 50 μM. The percent helicity was calculated based on the [*θ*]_222_ value. (b) HEK293T cells transfected with Topflash-luciferase were treated with Wnt3a conditioned medium (CM) and target peptides (40 μM) at the indicated concentrations for 12 h and then harvested for luciferase measurement. Con = non-specific control. (c) Proteolytic stability of the peptides (Axin (469–482) *vs.* WNT-**3a**) in the α-chymotrypsin solution (5 ng μL^–1^ in 50 mM PBS buffer, pH = 7.4) at a final concentration of 0.1 mM. Data points are displayed as the mean value SEM of duplicate independent experiments. The percent of residual peptide was monitored by analytical HPLC. (d) Confocal microscopic images of FITC–β-Ala–Axin, -**3a**, and -**3c** treated HEK 293T/17 cells. The cells were incubated with FITC–β-Ala–peptide (40 μM) for 16 h and then washed with PBS twice before fixation for confocal microscopy. Scale bar: 50 μm.

We next explored the effects of these peptides on Wnt/β-catenin dependent transcriptional activity. The Topflash reporter, in which firefly luciferase is transcriptionally activated by β-catenin, was employed. As shown in Fig. 3b, the new stapled peptides WNT-**1a**, -**3a**, -**4a**, -**5a**, -**6a** and -**8a** enhanced the Wnt3a-induced reporter expression more effectively than Axin (469–482), among which WNT-**3a**, -**5a**, and -**6a** treatment exhibited a three-fold enhancement. As expected, these stapled peptides promoted more Wnt3a-induced reporter expression than the corresponding regular stapled peptides WNT-**3b**, -**3c**, -**5b**, and -**6b**, demonstrating the benefit of maintaining the side chains of Met-481, Ser-471 and Leu-473. Among them, WNT-**3a** showed a more significant improvement than -**3b** and -**3c**, highlighting the influence of the side chain Met-481. As we expected, the activity of WNT-**3d**, the uncycled WNT-**3a**, showed less promotion when compared to Axin (469–482) owing to the remarkable weakness of linear peptides (Fig. S2†). All these results showcase the power of our new stapled peptides and the necessity of the retention of the specific side chain. It is worth noting that WNT-**2d** and -**7d** also significantly promoted Wnt3a-induced reporter expression as uncycled linear peptides, of which the most potent, WNT-**7d**, with a modified Met compared with WNT-**3a**, exhibited a three-fold increase in potency compared to the wild-type peptide. The failure of cyclizing WNT-**7d** makes us unable to evaluate the activity of WNT-**7a**, a potential potent activator of the Wnt signalling pathway.

Circular dichroism (CD) analysis of the peptides (Table 1 and Fig. 3a and S3†) indicates that the helicity of Axin (469–482) is only 20%, while the helicity of WNT-**1a** to WNT-**7d** ranges from 22% to 71% corresponding to a 1.1- to 3.6-fold increase. Consistent with the previous report,^26^ the “unstapled” analogue of WNT-**2d** and WNT-**7d** also displayed relatively high α-helicity, with an α-helical content value of 22% and 50%, respectively. These results demonstrate that our novel stapled peptide strategy can maintain the improvement of helicity when compared with the regular stapling strategy.

To test the protease stability of the stapled peptides with the retention of the side chains, we measured their susceptibility towards α-chymotrypsin-mediated degradation at room temperature in pH 7.4 PBS buffer containing 2 mM of CaCl_2_ as monitored by HPLC. α-Chymotrypsin is a protease that predominantly cleaves at the carboxyl side of positively charged amino acids such as methionine and leucine. Under these conditions, the half-life of Axin (469–482) is 9 min (Fig. 3c). In sharp contrast, WNT-**3a** is, as expected, completely stable and no degradation was observed even after 1 h. These results clearly demonstrate the inherent superiority of the new stapled peptides over linear peptides with respect to proteolytic stability.

**Fig. 4:**
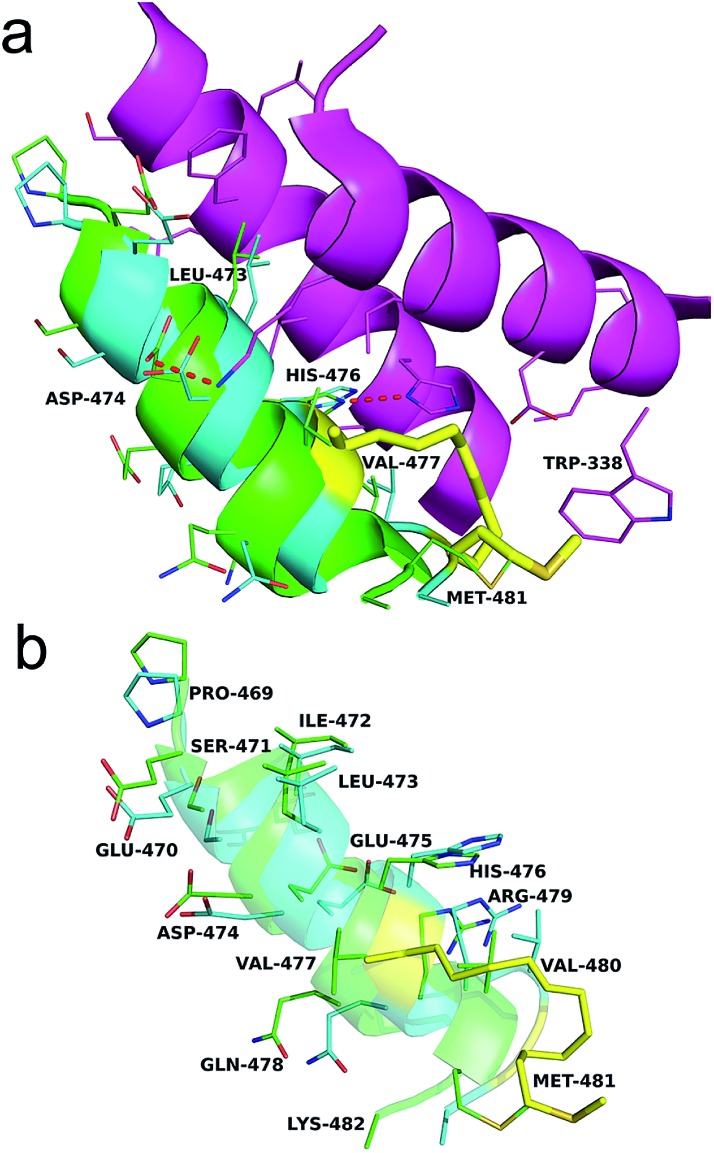
(a) The binding mode of Axin and WNT-**3a** with the β-catenin interface. Two critical helices of β-catenin (purple), Axin (green) and WNT-**3a** (blue) are shown. The stapling residues of WNT-**3a** are shown as a yellow stick. Red dashes represent the salt bridge and H-bond interactions. (b) A superposition inter-view of Axin (green) and WNT-**3a** (blue) bound to β-catenin. The stapling residues of WNT-**3a** are shown as a yellow stick.

We then assessed the cell permeability of the peptides by conjugating fluorescein isothiocyanate (FITC) to Axin (469–482), WNT-**3a** and WNT-**3c** (Fig. 3d). Our data show that FITC–β-Ala–WNT-**3a** and -**3c** efficiently entered into the cytoplasm in the HEK 293T/17 cells. Unexpectedly, FITC–β-Ala–Axin (469–482) also penetrated the cell membrane but showed weaker reporter expression in the presence of Wnt3a compared to WNT-**3a**, demonstrating the critical role of the native side chain of Met-481. To explore the detailed binding mode of WNT-**3a** against β-catenin, we conducted a Z-dock of the WNT-**3a**–β-catenin complex to predict structural models of the protein–protein complexes.^27^ As shown in Fig. 4a and b, subtle but important differences in the intermolecular interaction exist between WNT-**3a** and Axin (469–482). Owing to an introduction of the stapling group, Met-481 is closer to the Trp-338 of β-catenin, and thus forms a stronger interaction with that residue. In addition, a stronger salt bridge between the side chain carboxyl group of Asp-474 coordinated with the amino group of β-catenin Lys-292 and a more efficient hydrogen bond between the side chain of His-476 and β-catenin Asp-299 are constructed because of a closer distance geometrically. It is conceivable that these changes enhanced the conformational stability of WNT-**3a** in its bound state, thereby contributing to the improved binding activity.

## Conclusions

In conclusion, we have developed a new series of stapling amino acids, which contain the native amino acid side chains and can be applied in the standard stapling chemistry for stabilized α-helical conformation peptides. In this study, we have made use of these amino acids and S_5_/R_5_, to prepare potent inhibitors of the Axin–β-catenin interaction. We have demonstrated that incorporation of AA* at the *i* position of an *i*, *i* + 3 or *i*, *i* + 4 stapled peptide is a tolerated modification that allows the all-hydrocarbon staple to effectively mimic Axin, and exhibit superior protease stability. In this regard, this methodology is a more flexible and alternative strategy for stapled peptides with potential impact for related PPIs.

## Conflicts of interest

There are no conflicts to declare.

## References

[cit1] Azzarito V., Long K., Murphy N. S., Wilson A. J. (2013). Nat. Chem..

[cit2] Keskin O., Gursoy A., Ma B., Nussinov R. (2008). Chem. Rev..

[cit3] Mason J. M. (2010). Future Med. Chem..

[cit4] Guarracino D. A., Bullock B. N., Arora P. S. (2011). Biopolymers.

[cit5] Pellegrini M., Royo M., Chorev M., Mierke D. F. (1997). J. Pept. Res..

[cit6] Chorev M., Roubini E., McKee R. L., Gibbons S. W., Goldman M. E., Caulfield M. P., Rosenblatt M. (1991). Biochemistry.

[cit7] Cantel S., Le Chevalier Isaad A., Scrima M., Levy J. J., DiMarchi R. D., Rovero P., Halperin J. A., Ursi A. M. D., Papini A. M., Chorev M. (2008). J. Org. Chem..

[cit8] Lautrette G., Touti F., Lee H. G., Dai P., Pentelute B. L. (2016). J. Am. Chem. Soc..

[cit9] Schafmeister C. E., Po J., Verdine G. L. (2000). J. Am. Chem. Soc..

[cit10] Walensky L. D., Kung A. L., Escher I., Malia T. J., Barbuto S., Wright R. D., Wagner G., Verdine G. L., Korsmeyer S. J. (2004). Science.

[cit11] Moellering R. E., Cornejo M., Davis T. N., Del B. C., Aster J. C., Blacklow S. C., Kung A. L., Gilliland D. G., Verdine G. L., Bradner J. E. (2009). Nature.

[cit12] Baek S., Kutchukian P. S., Verdine G. L., Huber R., Holak T. A., Lee K. W., Popowicz G. M. (2012). J. Am. Chem. Soc..

[cit13] Vernall A. J., Cassidy P., Alewood P. F. (2009). Angew. Chem., Int. Ed..

[cit14] Cabezas E., Satterthwait A. C. (1999). J. Am. Chem. Soc..

[cit15] Chapman R. N., Dimartino G., Arora P. S. (2004). J. Am. Chem. Soc..

[cit16] Tian Y., Wang D. Y., Li J. X., Shi C., Zhao H., Niu X. G., Li Z. G. (2016). Chem. Commun..

[cit17] Cui H. K., Guo Y., He Y., Wang F. L., Chang H. N., Wang Y. J., Wu F. M., Tian C. L., Liu L. (2013). Angew. Chem., Int. Ed..

[cit18] Bird G. H., Crannell W. C., Walensky L. D. (2011). Curr. Protoc. Chem. Biol..

[cit19] Walter M. W., Thaker N., Baldwin J. E., Mueller M., Schofield C. J. (2000). J. Chem. Res..

[cit20] Krawczyk B., Ensle P., Müller W. M., Süssmuth R. D. (2012). J. Am. Chem. Soc..

[cit21] Ma D., Xia C., Jiang J., Zhang J., Tang W. (2003). J. Org. Chem..

[cit22] Cui H. K., Zhao B., Li Y. H., Guo Y., Hu H., Liu L., Chen Y. G. (2013). Cell Res..

[cit23] Ikeda S., Kishida S., Yamamoto H., Murai H., Koyama S., Kikuchi A. (1998). EMBO J..

[cit24] Clevers H., Nusse R. (2012). Cell.

[cit25] Pan M., Gao S., Zheng Y., Tan X., Lan H., Tan X., Sun D., Lu L., Wang T., Zheng Q., Huang Y., Wang J., Liu L. (2016). J. Am. Chem. Soc..

[cit26] Checco J. W., Lee E. F., Evangelista M., Sleebs N. J., Rogers K., Pettikiriarachchi A., Kershaw N. J., Eddinger G. A., Belair D. G., Wilson J. L., Eller C. H., Raines R. T., Murphy W. L., Smith B. J., Gellman S. H., Fairlie W. D. (2015). J. Am. Chem. Soc..

[cit27] Pierce B. G., Wiehe K., Hwang H., Kim B. H., Vreven T., Weng Z. (2014). Bioinformatics.

