# Supplementary material for: A novel peptide stapling strategy enables the retention of ring-closing amino acid side chains for the Wnt/β-catenin signalling pathway
Source: Chem Sci. 2017 Aug 29;8(11):7368–73. doi: 10.1039/c7sc02420g (PMC5672839; doi:10.1039/c7sc02420g)
Supplement: Supplementary file 1 [file SC-008-C7SC02420G-s001.pdf]

Electronic Supplementary Material (ESI) for Chemical Science.
This journal is © The Royal Society of Chemistry 2017

Supporting Information

A novel peptide stapling strategy enables retention of ring-closing amino acid side chains for Wnt/β-catenin signalling pathway

Ye Wu,[†a, d] Ye-Hua Li,[†b] Xiang Li,[†a] Yan Zou,[a] Hong-li Liao,[d] Lei Liu,[b] Ye-Guang Chen,[*b] Donald Bierer,[*c] and Hong-Gang Hu[*a]

a) School of Pharmacy, Second Military Medical University, Shanghai 200433, China. Email: huhonggang_fox@msn.com.

b) Tsinghua-Peking Center for Life Sciences, Tsinghua University, Beijing 100084, China. Email: ygchen@mail.tsinghua.edu.cn

c) Bayer AG, Department of Medicinal Chemistry, Aprather Weg 18A, 42096 Wuppertal, Germany. Email: donald.bierer1@bayer.com

d) School of Pharmacy, Chengdu Medical College, Chengdu 610083, China.

Table of Contents

1. General Information
2. Synthesis of amino acids Fmoc-AA*-OH
3. Synthesis and characterization of *α*-helix stapled, side chains retention peptides
4. Cell permeability
5. Protease stability
6. Spectrum
7. References

1. General Information

1.1 Materials

All reagents and solvents were purchased from J&K Scientific, GL Biotech, Energy Chemical or Sinopharm Chemical Reagent Co. Ltd and were purified when necessary. Rink Amide MBHA resin (0.28 mmol/g loading) was purchased from Tianjin Nankai Hecheng Science & Technology Co. Ltd. THF was distilled from sodium/benzophenone before use. DMF was distilled under reduced pressure from sodium sulfate and stored in flask containing 4 Å molecular sieves. Et_3N and CH_2Cl_2 were distilled from calcium hydride immediately prior to use. All organic extracts were dried over sodium sulfate and concentrated under rotary evaporator. All other commercially obtained reagents and solvents were used directly without further purification. TLC was performed on plates pre-coated with silica gel 60 F_{254} (250 layer thickness). Flash column chromatographic purification of products was finished using forced-flow chromatography on Silica Gel (300-400 mesh). Visualization was accomplished with 5% (v/v) H_2SO_4 in EtOH, UV light, and/or phosphomolybdic acid (PMA) solution.

1.2 ^{1}H and ^{13}C nuclear magnetic resonance spectra

^{1}H-NMR and ^{13}C-NMR spectra were obtained on a Bruker Avance 400 MHz or 600 MHz NMR Spectrometer. The chemical shifts of protons are given on the δ scale, ppm, with tetramethylsilane (TMS) as the internal standard. All NMR experiments were conducted at room temperature unless otherwise stated. The ^{1}H-NMR spectra are reported as follows: δ/ppm (multiplicity, coupling constant J /Hz, number of protons). Multiplicity is abbreviated as follows: s = singlet, d = doublet, t = triplet, m = multiplet, dd = doublet of doublet. Coupling constants (J) are quoted in Hertz and recorded to the nearest 0.1 Hz. The ^{13}C-NMR spectra are reported in δ/ppm.

1.3 High resolution mass spectra

HR-Q-TOF-MS was measured on an Agilent 6538 UHD Accurate Mass Q-TOF LC/MS mass spectrometer.

1.4 Reversed phase analytical HPLC

Analytical HPLC was run on a SHIMADZU (Prominence LC-20AD) instrument using an analytical column (Grace Vydac “Protein & Peptide C18”, 250 × 4.6 mM, 5 μm particle size, flow rate 1.0 mL/min, r.t.). Analytical injections were monitored at 214 nm, 254 nm.

1.5 Purification of peptides by reversed phase preparative HPLC

Semi preparative HPLC was run on a SHIMADZU (LC-6A) instrument using a semi preparative column (Grace Vydac “Peptide C18”, 250 × 10 mM, 10 μm particle size, flow rate 4 mL/min). Solution A was 0.1% TFA in water, and solution B was 0.1% TFA in MeCN. Gradient A: A linear gradient of 10% to 10% B over 2 mins, then a linear gradient of 10% to 90% B over 25 mins. Gradient B: A linear gradient of 1% to 1% B over 3 mins, then a linear gradient of 1% to 35% B over 25 mins.

1.6 Solid Phase Peptide Synthesis

Solid phase peptide synthesis was carried out as described by Kim, Grossman and Verdine[1]. As a typical example, 400 mg Rink Amide MBHA resin was swelled with DCM (5 mL) for 20 mins. Then the resin was treated with 20% piperidine/DMF twice (10 and 5 mins), followed by washing with DMF (5 times), DCM (5 times) and DMF (5 times). For coupling of the first amino acid, Fmoc-AA-OH (1 mmol), HCTU (0.9 mmol), DIEA (2 mmol) and DMF (6 mL) were mixed for 2 mins and then added to the resin. After 2 hrs, the resin was washed with DMF (5 times), DCM (5 times), and DMF (5 times). The peptide couplings of N-Fmoc-α-pentene amino acids and S_5/R_5 were carried out over a single two hours coupling cycle using 2 eq. of the Fmoc protected amino acids. The deprotection, washing, coupling and washing steps were repeated until all the amino acid residues were assembled reagent. The peptide-bound resin was treated with 20% piperidine/DMF to remove the Fmoc group from the N-terminus. After the resin was washed it was treated with 3 mL solution of acetic anhydride and pyridine (1:1) for 20 mins. Then the resin was washed with DMF (5 times), DCM (5 times), and DMF (5 times). The ring-closing metathesis reaction was carried out in

1,2-dichloroethane (DCE) at room temperature (20-25 °C) using Grubbs' first-generation catalyst, The resin was washed with DCM (3 times), and DCE (3 times) and then treated with 10 mM solution of Grubbs' first-generation catalyst in DCE. After the first round of the 2 hrs metathesis, we repeated the same procedure for a second round of catalyst treatment with fresh catalyst solution, the peptide-resin was washed with DMF (5 times), DCM (5 times). Peptides were cleaved from their resin by treatment with reagent K (80% TFA, 5%, H_2O, 2.5% EDT, 5% Thioanisole and 7.5% phenol) for 4 hrs at room temperature. After completion of the cleavage reaction, TFA was evaporated by blowing with Ar. The crude peptides were obtained by precipitation with 40 mL of cold diethyl ether and centrifugation at 3500 r/min for 3 mins (3 times). The supernatant diethyl ether was decanted from the centrifuge tube and the crude peptides were allowed to air dry.

2. Synthesis of amino acids N-Fmoc-*α*-pentene amino acids

2.1 Synthesis of 3a-3f

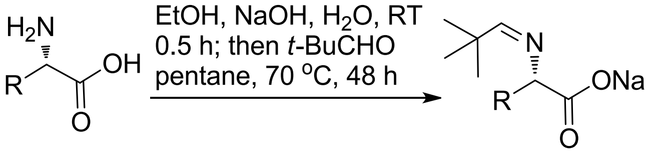

2a-2f **3a-3f**

2.1.1 sodium (S, E)-2-((2,2-dimethylpropylidene)amino)-4-methylpentanoate (3a)

The commercially available **2a** (30 g, 229 mmol) was dissolved in anhydrate EtOH (500 mL), then the solution of NaOH (9.13 g, 229 mmol) in water (60 mL) was added. The mixture was then allowed to room temperature and stirred for 0.5 hrs. Then the solution was concentrated *in vacuo* and the residue was added *n*-pentane (350 mL) and trimethylacetaldehyde (30 g, 344 mmol). The reaction mixture was then stirred at refluxing for 2 days (equipped with a water separator). After 48 hrs, the solution was concentrated *in vacuo* and the solid was dried to yield **3a** as white solid powder, which was used without further purification.

IR (thin film, cm^{-1}): 3416, 2958, 2938, 2870, 2745, 2630, 1601, 1583, 1553, 1518, 1483, 1458, 1411,

1387, 1362, 1316, 1296, 1225, 1207, 1188, 1175, 1136, 1086, 1057, 1030, 1005, 980, 943, 918, 893, 847, 835, 806, 770, 735, 669, 627, 602, 540, 503.

2.1.2 sodium (S, E)-2-((2,2-dimethylpropylidene)amino)-4-(methylthio)butanoate (3b)

This compound was prepared following the procedure described for **3a** as white solid powder.

IR (thin film, cm^{-1}): 3416, 3173, 2957, 2922, 2870, 2745, 2369, 2143, 1763, 1665, 1599, 1586, 1555, 1508, 1483, 1448, 1412, 1362, 1316, 1286, 1277, 1260, 1244, 1225, 1207, 1188, 1173, 1121, 1069, 1057, 1030, 1005, 980, 966, 953, 943, 914, 893, 876, 806, 770, 750, 725, 692, 679, 656, 644, 627, 615, 594, 544, 476, 448, 438, 424..

2.1.3 sodium (S, E)-3-(tert-butoxy)-2-((2,2-dimethylpropylidene)amino)propanoate (3c)

This compound was prepared following the procedure described for **3a** as white solid powder.

2.1.4 sodium (S, E)-3-(4-(tert-butoxy)phenyl)-2-((2,2-dimethylpropylidene)amino) propanoate (3d)

This compound was prepared following the procedure described for **3a** as white solid powder.

2.1.5 sodium (S, E)-6-azido-2-((2,2-dimethylpropylidene)amino)hexanoate (3e)

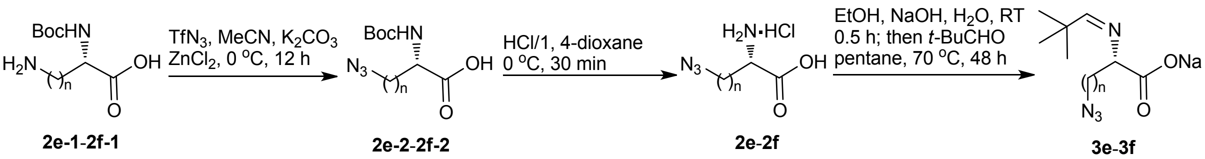

2e-1-2f-1 2e-2-2f-2 2e-2f 3e-3f

Tf_2O (40 g, 141.84 mmol) was added dropwise into a solution of NaN_3 (9.22 g, 141.84 mmol) in MeCN (140 mL) at 0 °C. The reaction mixture was stirred at 0 °C for 30 mins. The solid was washed with MeCN (100 mL), and the filtrate was used directly in the next step. A mixture of commercially available **Boc-Lys-OH (2e-1)** (17.47 g, 70.92 mmol), K_2CO_3 (19.58 g, 141.84 mmol) and $ZnCl_2$ (308 mg, 2.26 mmol) in MeCN (340 mL) and water (340 mL) was added freshly prepared TfN_3 dropwise at 0 °C. Then the reaction mixture was stirred at 0 °C

for additional 2 hrs. The mixture was then allowed to warm to room temperature and stirred overnight. The MeCN was removed *under vacuo* and the aqueous solution was extracted with EA (3 × 400 mL). The organic layer was washed with saturated NaCl (1 × 400 mL), dried over Na_2SO_4, concentrated and purified by column chromatography (20:1 DCM/MeOH) to yield **Boc-Lys(6-Azide)-OH** (**2e-2**) as yellow oil, which was used without further purification.

^{1}H NMR (600 MHz, $CDCl_3$): δ 4.11-4.08 (m, 1H), 1.86-1.81 (m, 1H), 1.70-1.45 (m, 16H).

^{13}C NMR (150 MHz, MeOD): δ 174.3, 156.3, 78.7, 52.8, 50.4, 30.5, 27.6, 26.9 (2C), 26.7, 22.3.

HR-Q-TOF-MS *m/z* calcd for $C_{11}H_{20}N_4O_4$ 272.1481; found $[M-H]^-$ 271.1408.

A solution of 4.0 M HCl in 1,4-dioxane (100 mL, 400 mmol) was added into dried **2e-2** (29.95 g, 110 mmol) under the protecttion of Ar at 0 ^{o}C and the reaction mixture was stirred at room temperature for 30 mins to form a precipitate to yield **H-Lys(6-Azide)-OH** (**2e**) as white solid powder, which was used without further purification. The solid was washed with Et_2O, dried *under vacuo* and in anhydrous alcohol was added NaOH (8.80 g, 220 mmol) in water (60 mL) and the mixed solution was stirred at room temperature for 30 mins. Then the solution was removed *under vacuo* to yield **H-Lys(6-Azide)-ONa** (17.56 g, 82.23 %) as white solid powder.

The **3e** was prepared following the procedure described for **3a**.

2.1.6 sodium (S, E)-5-azido-2-((2,2-dimethylpropylidene)amino)pentanoate (3f)

This compound was prepared following the procedure described for **3e.**

Boc-Orn (5-Azide)-OH (2f-2) as yellow oil.

^{1}H NMR (600 MHz, MeOD): δ 4.14-4.13 (m, 1H), 1.96-1.94 (m, 1H), 1.77-1.78(m, 3H), 1.48 (m, 11H).

^{13}C NMR (150 MHz, MeOD): δ 174.5, 156.7, 79.2, 53.0, 50.6, 28.7, 27.3 (2C), 27.2, 25.0.

HR-Q-TOF-MS *m/z* calcd for $C_{10}H_{18}N_4O_4$ 258.1331; found $[M-H]^-$ 257.1258.

H-Orn (5-Azide)-ONa (5.20 g, 75 %) as white solid powder.

2.2 Synthesis of 4a-4g.

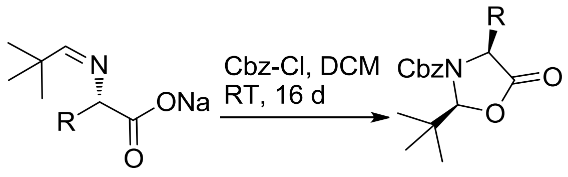

3a-3f **4a-4f**

2.2.1 (2S, 4S)-benzyl 2-(tert-butyl)-4-isobutyl-5-oxooxazolidine-3-carboxylate (4a)

Benzyl carbonochloridate (59 g, 344 mmol) was added into **3a** (50.84 g, 229 mmol) in DCM (500 mL) dropwise at 0 ^{o}C and the mixture was allowed to warm to room temperature and stirred for 16 days. The reaction mixture was stirred for additional 24 hrs after DMAP (4-dimethylaminopyridine, 60 mg) and water (500 mL) were added in. The DCM was removed *under vacuo* and the aqueous solution was extracted with EA (3 × 400 mL). The organic layer was washed with 10% $NaHSO_4$ (500 mL), saturated $NaHCO_3$ (500 mL) and saturated NaCl (500 mL), dried over Na_2SO_4, concentrated and purified by column chromatography (100:1 PE/EA) to yield **4a** (57.27 g, 50 % over two steps) as colorless oil.

^{1}H NMR (600 MHz, $CDCl_3$): δ 7.42-7.30 (m, 5H), 5.58 (s, 1H), 5.22-5.18 (dd, J = 16.44, 4.5 Hz, 2H), 4.37-4.35 (t, J = 13.56 Hz, 1H), 2.07-2.01 (m, 1H), 1.85-1.80 (m, 1H), 1.71-1.66 (m, 1H), 1.00-0.96 (m, 15H).

^{13}C NMR (150 MHz, $CDCl_3$): δ 173.0, 156.1, 135.3, 128.7 (3C), 128.5 (3C), 96.3, 68.4, 55.6, 42.5, 36.9, 29.7, 25.0 (3C), 25.0 (3C), 22.7, 22.0.

HR-Q-TOF-MS *m/z* calcd for $C_{19}H_{27}NO_4$ 333.1941; found $[M+H]^+$ 334.2014;$[M+Na]^+$ 356.1835.

2.2.2 (2S, 4S)-benzyl 2-(tert-butyl)-4-(2-(methylthio)ethyl)-5-oxooxazolidine-3-arboxylate (4b)

This compound was prepared following the procedure described for **4a** (42.36 g, 60 % over two steps) as colorless oil.

^{1}H NMR (600 MHz, $CDCl_3$): δ 7.38-7.37 (m, 5H), 5.58-5.57 (d, J = 6.2 Hz, 1H), 5.19-5.17

(m, 2H), 4.55-4.52 (t, J = 14.1, 1H), 2.76-2.73 (dd, J = 14.5, 7.9 Hz, 2H), 2.20-2.02 (m, 5H), 0.98- 0.97 (m, 9H).

^{13}C NMR (150 MHz, $CDCl_3$): δ 171.9, 155.4, 134.7, 128.2 (2C), 128.1 (2C), 95.9, 67.9, 55.3, 36.5, 31.8, 30.4, 30.1, 24.4 (2C), 24.3, 14.5.

HR-Q-TOF-MS ***m/z*** calcd for $C_{18}H_{25}NO_4S$ 351.1508; found $[M+H]^+$ 352.1581;$[M+Na]^+$ 374.1400.

2.2.3 (2S, 4S)-benzyl 4-(tert-butoxymethyl)-2-(tert-butyl)-5-oxooxazolidine-3-carboxylate (4c)

This compound was prepared following the procedure described for **4a** (13.51 g, 30 % over two steps) as colorless oil.

^{1}H NMR (600 MHz, $CDCl_3$): δ 7.41-7.38 (m, 5H), 5.69 (s, 1H), 5.32-5.30 (d, J = 12.1 Hz, 1H), 5.09-5.07 (d, J = 11.2 Hz, 1H), 4.14 (s, 1H), 3.64-3.62 (d, J = 8.5 Hz, 1H), 1.67 (s, 1H), 1.09 (s, 9H), 0.98 (s, 9H).

^{13}C NMR (150 MHz, $CDCl_3$): δ 172.3, 135.6, 128.6 (2C), 128.5 (3C), 95.7, 73.2, 67.5, 58.8, 39.1, 27.2 (4C), 24.6 (4C).

HR-Q-TOF-MS ***m/z*** calcd for $C_{20}H_{29}NO_5$ 363.2049; found $[M+Na]^+$ 386.1941; $[M+K]^+$ 402.1681.

2.2.4 (2S, 4S)-benzyl 4-(4-(tert-butoxy)benzyl)-2-(tert-butyl)-5-oxooxazolidine-3-carboxylate (4d)

This compound was prepared following the procedure described for **4a** (20.00 g, 54 % over two steps) as colorless oil.

^{1}H NMR (600 MHz, $CDCl_3$): δ 7.37-7.35 (m, 3H), 7.29-7.28 (m, 2H), 7.10-7.09 (d, J = 8.0 Hz, 2H), 6.86-6.84 (d, J = 8.4 Hz, 2H), 5.53 (s, 1H), 5.16-5.14 (d, J = 11.9 Hz, 1H), 4.96- 4.94 (d, J = 11.6 Hz, 1H), 4.46-4.44 (dd, J = 7.6, 2.5 Hz, 1H), 3.19-3.16 (m, 1H), 3.09-3,06 (m, 1H), 1.31 (s, 9H), 0.95 (s, 9H).

^{13}C NMR (150 MHz, $CDCl_3$): δ 172.1, 155.9, 154.4, 135.2, 131.6, 130.1 (2C), 128.7 (2C),

128.7, 128.6 (2C), 124.1 (2C), 96.2, 78.4, 68.3, 59.1, 38.5, 37.1, 28.9 (3C), 24.9 (3C).

HR-Q-TOF-MS ***m/z*** calcd for $C_{26}H_{33}NO_5$ 439.2362; found $[M+H]^+$ 440.2440; $[M+NH_4]^+$ 457.2702; $[M+Na]^+$ 462.2254.

2.2.5 (2S, 4S)-benzyl 4-(4-azidobutyl)-2-(tert-butyl)-5-oxooxazolidine-3-carboxylate (4e)

This compound was prepared following the procedure described for **4a** (36.70 g, 77 % over two steps) as colorless oil.

^{1}H NMR (600 MHz, $CDCl_3$): δ 7.43-7.38 (m, 5H), 5.60 (s, 1H), 5.23-5.19 (dd, *J* =17.0, 5.0 Hz 2H), 4.30-4.28 (t, *J* = 14.4 Hz, 1H), 3.26-3.24 (t, *J* = 11.2 Hz, 2H), 2.00-1.94 (m, 1H), 1.89-1.83 (m, 1H), 1.67-1.60 (m, 4H), 1.00 (s, 9H).

^{13}C NMR (150 MHz, $CDCl_3$): δ 172.6, 156.0, 135.2, 128.7 (3C), 128.6 (2C), 96.4, 68.5, 57.0, 51.0, 367.0, 32.8, 28.4, 24.9 (3C), 23.6.

HR-Q-TOF-MS ***m/z*** calcd for $C_{19}H_{26}N_4O_4$ 374.1959; found $[M+Na]^+$ 397.1851; $[M+K]^+$ 413.1590.

2.2.6 (2S, 4S)-benzyl 4-(3-azidopropyl)-2-(tert-butyl)-5-oxooxazolidine-3-carboxylate (4f)

This compound was prepared following the procedure described for **4a** (2.36 g, 25 % over two steps) as colorless oil.

^{1}H NMR (600 MHz, $CDCl_3$): δ 7.42-7.37 (m, 5H), 5.58 (s, 1H), 5.20 (s, 2H), 4.29-4.28 (m, 1H), 1.44 (s, 2H), 1.35-1.27 (m, 4H), 0.98 (s, 9H).

^{13}C NMR (150 MHz, $CDCl_3$): δ 173.8, 157.4, 136.6, 130.2 (2C), 130.0 (2C), 97.9, 70.00, 58.2, 52.2, 38.4, 31.9, 31.7, 27.2, 26.3 (3C).

HR-Q-TOF-MS ***m/z*** calcd for $C_{18}H_{24}N_4O_4$ 360.1807; found $[M+Na]^+$ 383.1700.

2.2.7 (2S, 4S)-benzyl 4-benzyl-5-oxo-2-phenyloxazolidine-3-carboxylate (4g)

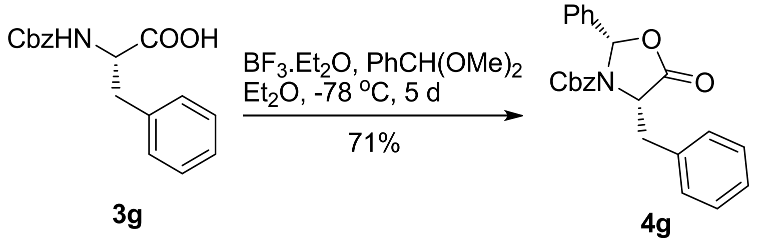

The commercially available **3g** (30 g, 100 mmol) and benzaldehyde dimethyl acetyl (14.4 mL, 96 mmol) were dissolved in anhydrate Et_2O (500 mL) and the solution was cooled to -78 °C, then the $BF_3{\cdot}Et_2O$ (60.2 mL, 489 mmol) was added. The mixture was then allowed to room temperature and stirred for 7 days. The saturated aqueous $NaHCO_3$ solution was added until strong bubbling ceased after the reaction mixture was cooled to 0 °C. The mixture was extracted with Et_2O (3 × 150 mL), dried over Na_2SO_4, concentrated and purified by column chromatography (20:1 PE/EA) to yield **4g** (24.3 g, 71 %) as white solid powder.

^{1}H-NMR (400 MHz, $CDCl_3$): δ 7.38-7.13 (m, 13H,), 6.72 (s, 2H), 6.48(s, 1H), 5.23-5.08 (dd, *J* = 12.1 Hz, 2H), 4.72-4.69 (m, 1H), 3.35-3.30 (m, 2H).

^{13}C NMR (150 MHz, $CDCl_3$): δ 171.1, 154.2, 136.3, 135.3, 130.3, 129.4, 128.8 (3C), 128.6, 128.5, 128.3 (3C), 128.2, 127.3, 126.7, 126.5, 89.3 (3C), 68.1, 58.3, 29.7.

HR-Q-TOF-MS *m/z* calcd for $C_{24}H_{21}NO_4$ 387.1475; found $[M+H]^+$ 388.1548; $[M+Na]^+$ 410.1365.

2.3 Synthesis of 5a-5g.

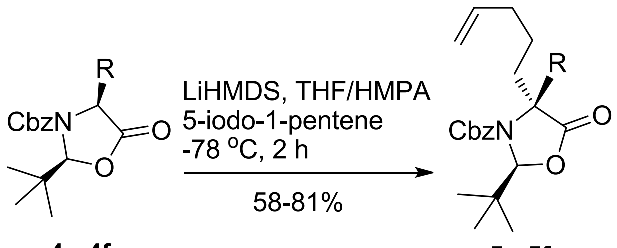

2.3.1 (2S, 4S)-benzyl 2-(tert-butyl)-4-isobutyl-5-oxo-4-(pent-4-en-1-yl)oxazolidine-3-carboxylate (5a)

A mixture of **4a** (4.33 g, 13.0 mmol) and 5-iodo-1-pentene (5.1 g, 26.0 mmol) were dissolved in dry THF/HMPA (4:1, 150 mL) at -78 °C under the protection of Ar. 1 M LiHMDS (26 mL, 26 mmol) in THF was slowly added into the reaction solution and stirred for 2 hrs. The reaction mixture was then quenched by saturated NH_4Cl solution (100 mL) and extracted with EA twice (2 × 100 mL). The organic layer was washed with saturated $NaHCO_3$ (2 × 50 mL), 10 % $Na_2S_2O_3$ (2 × 50 mL) and NaCl (2 × 50 mL), dried over Na_2SO_4, concentrated and

purified by column chromatography (40:1 PE/EA) to yield **5a** (4.01 g, 77%) as colorless oil.

^{1}H NMR (600 MHz, $CDCl_3$): *δ* 7.42-7.40 (m, 5H), 5.64-5.55 (m, 1H), 5.55 (s, 1H), 5.20 (s, 2H), 4.96-4.93 (m, 2H), 1.96-1.80 (m, 4H,), 1.37-1.29 (m, 2H), 1.12- 0.95 (m, 18H)

^{13}C NMR (150 MHz, $CDCl_3$): *δ* 174.8, 137.6, 135.3, 128.9 (2C), 128.7 (3C), 115.2, 95.2, 67.8, 67.1, 46.5, 38.1, 33.1, 29.7, 25.6 (4C), 24.9, 24.7, 23.6, 23.1.

HR-Q-TOF-MS *m/z* calcd for $C_{24}H_{35}NO_4$ 401.2573; found $[M+H]^+$ 402.2646; $[M+Na]^+$ 424.2459.

2.3.2 (2S, 4S)-benzyl 2-(tert-butyl)-4-(2-(methylthio)ethyl)-5-oxo-4-(pent-4-en-1-yl)oxazolidine-3-carboxylate (5b)

This compound was prepared following the procedure described for **5a** (9.41 g, 79 %) as colorless oil.

^{1}H NMR (600 MHz, $CDCl_3$): *δ* 7.34-7.33 (m, 5H), 5.55-5.50 (m, 2H), 5.13 (s, 2H), 4.89- 4.87 (d, *J* = 12.7 Hz, 2H), 2.75 (s, 1H), 2.52 (s, 1H), 2.29-2.10 (m, 3H), 2.02 (s, 3H), 1.81- 1.61 (m, 3H), 1.03-0.90 (m, 11H).

^{13}C NMR (150 MHz, $CDCl_3$): *δ* 173.6, 136.9, 134.7, 128.5 (2C), 128.3 (2C), 128.2 (2C), 114.9, 94.8, 67.4, 65.8, 37.6, 37.3, 32.4, 28.3, 25.0 (4C), 22.5, 14.9.

HR-Q-TOF-MS *m/z* calcd for $C_{23}H_{33}NO_4S$ 419.2132; found $[M+H]^+$ 420.2210; $[M+Na]^+$ 442.2024.

2.3.3 (2S, 4S)-benzyl 4-(4-azidobutyl)-2-(tert-butyl)-5-oxo-4-(pent-4-en-1-yl)oxazolidine-3-carboxylate (5c)

This compound was prepared following the procedure described for **5a** (3.79 g, 64 %) as colorless oil.

^{1}H NMR (600 MHz, $CDCl_3$): *δ* 7.38-7.35 (m, 5H), 5.59-5.47 (m, 2H), 5.26-5.14 (m, 2H), 4.91-4.88 (m, 2H), 3.77 (s, 1H), 3.58-3.53 (m, 1H), 2.00-1.70 (m, 3H), 1.54-1.49 (m, 1H), 1.27 (s, 9H), 1.02-1.01 (d, *J* = 2.2 Hz, 11H).

^{13}C NMR (150 MHz, $CDCl_3$): δ 173.1, 137.5, 135.4, 128.8 (2C), 128.7 (2C), 128.7, 115.4, 95.3, 73.7, 67.6, 64.2, 60.4, 33.0, 27.3 (3C), 26.1 (2C), 25.6, 24.5, 22.4, 14.0.

HR-Q-TOF-MS *m/z* calcd for $C_{25}H_{37}NO_5$ 431.2672; found $[M+H]^+$ 432.2749; $[M+Na]^+$ 454.2571.

2.3.4 (2S, 4S)-benzyl 4-(4-(tert-butoxy)benzyl)-2-(tert-butyl)-5-oxo-4-(pent-4-en-1-yl)oxazolidine-3-carboxylate (5d)

This compound was prepared following the procedure described for **5a** (9.41 g, 81 %) as colorless oil.

^{1}H NMR (600 MHz, $CDCl_3$): δ 7.43-7.39 (m, 5H), 7.10 (s, 2H), 6.87-6.85 (d, J = 8.3 Hz, 2H), 5.62-5.55 (m, 1H), 5.41-5.19 (m, 3H), 4.94-4.91 (m, 2H), 3.24-3.18 (m, 2H), 1.82-1.64 (m, 4H), 1.32 (s, 9H), 1.24-1.20 (m, 2H), 0.56 (s, 9H).

^{13}C NMR (150 MHz, $CDCl_3$): δ 175.3, 156.0, 138.9, 136.6, 133.0 (2C), 132.3, 130.6, 130.2 (2C), 125.7, 125.6 (2C), 116.8, 96.8, 79.8, 70.7, 69.4, 43.1, 39.2, 34.4, 30.3, 30.1 (4C), 26.5 (3C), 26.2, 24.3.

HR-Q-TOF-MS *m/z* calcd for $C_{31}H_{41}NO_5$ 507.2985; found $[M+H]^+$ 508.3065; $[M+NH_4]^+$ 525.3332; $[M+Na]^+$ 530.2883.

2.3.5 (2S, 4S)-benzyl 4-(4-azidobutyl)-2-(tert-butyl)-5-oxo-4-(pent-4-en-1-yl)oxazolidine-3-carboxylate (5e)

This compound was prepared following the procedure described for **5a** (2.78 g, 79 %) as colorless oil.

^{1}H NMR (600 MHz, $CDCl_3$): δ 7.38-7.37 (m, 5H), 5.61-5.53 (m, 2H), 5.17 (s, 2H), 4.93-4.90 (m, 2H), 3.25-3.23 (t, J = 13.1 Hz, 2H), 2.03-1.66 (m, 7H), 1.46 (s, 1H), 1.33-1.26 (m, 1H), 1.13-1.07 (m, 1H), 0.91- 0.87 (m, 11H).

^{13}C NMR (151 MHz, $CDCl_3$): δ 174.1, 154.6, 137.0, 134.8, 128.5 (2C), 128.2 (2C), 114.8, 94.7, 67.3, 66.1, 50.6, 37.6, 37.0, 32.5, 29.2, 28.7, 25.1 (3C), 22.6, 21.1, 13.7.

HR-Q-TOF-MS *m/z* calcd for $C_{24}H_{34}N_4O_4$ 442.26, found $[M+H]^+$ 443.2656; $[M+NH_4]^+$

460.2916, [M+Na]+ 465.2477.

2.3.6 (2S, 4S)-benzyl 4-(3-azidopropyl)-2-(tert-butyl)-5-oxo-4-(pent-4-en-1-yl) oxazolidine-3-carboxylate (5f)

This compound was prepared following the procedure described for **5a** (1.34 g, 58 %) as colorless oil.

^{1}H NMR (600 MHz, $CDCl_3$): *δ* 7.39-7.36 (m, 5H), 5.60-5.52 (m, 2H), 5.17 (s, 2H), 4.92-4.90 (d, *J* = 11.7 Hz, 2H), 2.13-1.64 (m, 8H), 1.29-1.26 (m, 1H), 1.13-1.07 (m, 1H), 0.98-0.92 (m, 11H).

^{13}C NMR (150 MHz, $CDCl_3$): *δ* 174.4, 155.5, 137.4, 135.2, 128.9 (2C), 128.8 (2C), 115.4, 95.3, 67.9, 66.3, 51.4, 51.4, 38.1, 35.1, 32.9, 29.5, 25.5 (3C), 23.9, 23.0.

HR-Q-TOF-MS *m/z* calcd for $C_{23}H_{32}N_4O_4$ 428.2424; found [M+Na]+ 451.2322.

2.3.7 (2S, 4S)-benzyl 4-benzyl-5-oxo-4-(pent-4-en-1-yl)-2-phenyloxazolidine-3-carboxylate (5g)

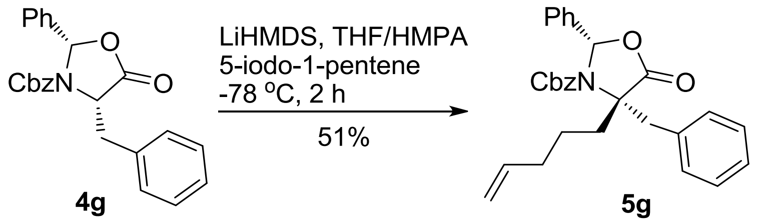

This compound was prepared following the procedure described for **5a** (3.00 g, 51 %) as colorless oil.

^{1}H-NMR (400 MHz, $CDCl_3$): *δ* 7.52-7.11 (m, 11H), 7.06-6.97 (m, H), 6.78-6.75 (d, *J* = 7.2 Hz, 1H), 6.33-6.13 (d, *J* = 7.2 Hz, 1H), 6.12 (s,1H), 5.86- 5.57 (m, 1H), 5.44-4.91 (d, *J* = 12.0 Hz, 1H), 5.20-4.94 (m, 2H), 3.65-3.32 (d, *J* = 13.2 Hz, 1H), 3.39-3.25 (m, 1H), 2.69-2.18 (m, 1H), 2.17- 2.07 (m, 1H), 2.05-1.80 (m, 2H), 1.50-1.15 (m, 2H).

^{13}C NMR (150 MHz, $CDCl_3$): *δ* 173.0, 151.6, 137.0, 136.8, 135.6, 135.6, 135.9, 134.9, 134.8, 130.2, 128.9, 128.9, 128.6, 128.4, 128.4, 127.8, 127.6, 127.4, 127.1, 126.9, 126.7, 89.4, 68.6, 66.8, 40.7, 35.3, 32.6, 23.0, 22.8.

HR-Q-TOF-MS ***m/z*** calcd for $C_{29}H_{29}NO_4$ 455.2102; found $[M+Na]^+$ 478.1994.

2.4 Synthesis of 6a-6g.

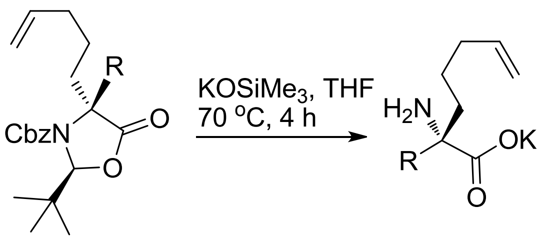

5a-5f **6a-6f**

2.4.1 potassium (S)-2-amino-2-isobutylhept-6-enoate (6a)

The mixture of **5a** (1 g, 2.49 mmol) and $KOSiMe_3$ (90 % pure, 1.06 g, 7.47 mmol) in dry THF (55 mL) was stirred for 4 hrs at 70 °C. MeOH (40 mL) was added and the reaction mixture was concentrated *in vacuo* to yield **6a** as yellow oil, which was used without further purification.

2.4.2 potassium (S)-2-amino-2-(2-(methylthio)ethyl)hept-6-enoate (6b)

This compound was prepared following the procedure described for **6a** as yellow oil.

2.4.3 potassium (S)-2-amino-2-(tert-butoxymethyl)hept-6-enoate (6c)

This compound was prepared following the procedure described for **6a** as yellow oil.

2.4.4 potassium (S)-2-amino-2-(4-(tert-butoxy)benzyl)hept-6-enoate (6d)

This compound was prepared following the procedure described for **6a** as yellow oil.

2.4.5 potassium (S)-2-amino-2-(4-azidobutyl)hept-6-enoate (6e)

This compound was prepared following the procedure described for **6a** as yellow oil.

2.4.6 potassium (S)-2-amino-2-(3-azidopropyl)hept-6-enoate (6f)

This compound was prepared following the procedure described for **6a** as yellow oil.

2.4.7 potassium (S)-2-amino-2-benzylhept-6-enoate (6g)

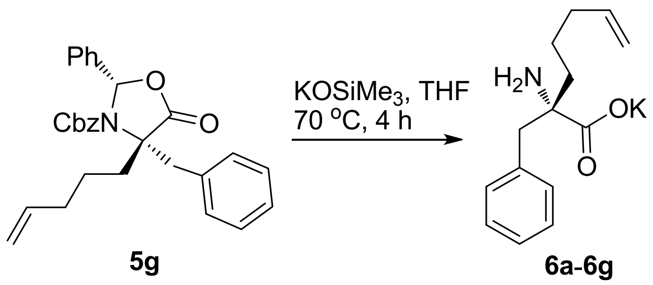

5g **6a-6g**

This compound was prepared following the procedure described for **6a** as yellow oil.

2.5 Synthesis of 1a-1g.

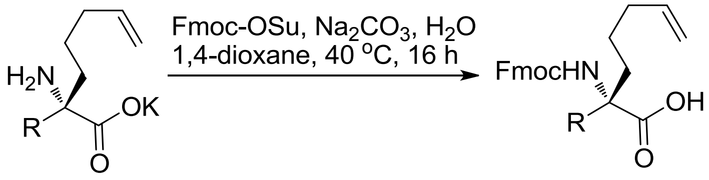

6a-6g **1a-e** and **1g**

2.5.1 (S)-2-((((9H-fluoren-9-yl)methoxy)carbonyl)amino)-2-isobutylhept-6-enoic acid (1a)

The **6a** was suspended in a mixture of 10 % aqueous Na_2CO_3 (1.85g, 17.43 mmol, 20.66 mL) and 1,4-dioxane (4.43 mL) and cooled in an ice bath. Fmoc-OSu (4.20 g, 12.45 mmol) was dissolved in 1,4-dioxane (15.43 mL) by gentle heating and the solution was added over 30 mins *via* a dropping funnel with efficient stirring. The reaction mixture was stirred for 16 hrs at 40 °C and the solvent was removed *in vacuo*. The dioxane was evaporated and water suspension was diluted with H_2O (200 mL), washed with Et_2O (2 × 100 mL) and the aqueous phase was acidified with citric acid to pH 2~3 (pH paper). The aqueous phase was extracted with EA (3 × 100mL), the organic phases were combined, dried (Na_2SO_4), filtered, the solvent was removed *in vacuo* and purified by column chromatography (100:1 DCM/MeOH) to yield **1a** (0.31 g, 30% over two steps) as white solid powder. $[\alpha]_D^{29}$= -16.1 (*c* 0.33, $CHCl_3$)

^{1}H NMR (600 MHz, MeOD): *δ* 7.85-7.80 (m, 2H), 7.66-7.59 (m, 2H), 7.41-7.30 (m, 5H), 7.23-7.12 (m, 1H), 5.80-5.73 (m, 1H), 5.00- 4.97 (m, 1H), 4.38-4.37 (m, 2H), 4.24-4.21 (dd, *J* = 15.3, 6.8 Hz, 1H), 2.22-2.16 (m, 2H), 2.03-1.99 (m, 2H), 1.77-1.54 (m, 3H), 1.35-1.29 (m, 2H), 0.89-0.85 (m, 6H).

^{13}C NMR (150 MHz, MeOD): *δ* 177.2, 155.9, 145.3, 145.3, 142.6, 139.5, 134.1, 130.7, 129.9,

129.2, 128.8, 128.2, 128.2, 126.1, 121.0, 115.2, 67.2, 64.0, 44.9, 44.8, 36.7, 34.6, 25.8, 24.4, 24.3, 23.4.

HR-Q-TOF-MS ***m/z*** calcd for $C_{26}H_{31}NO_4$ 421.2258; found $[M+H]^+$ 422.2330; $[M+Na]^+$ 444.2152; $[M+K]^+$ 460.1885.

2.5.2 (S)-2-((((9H-fluoren-9-yl)methoxy)carbonyl)amino)-2-(2-(methylthio)ethyl)hept-6-enoic acid (1b)

This compound was prepared following the procedure described for **1a** (5.02 g, 75% over two steps) as white solid powder. $[\alpha]_D^{29}$= +3.3 (*c* 0.33, $CHCl_3$)

^{1}H NMR (600 MHz, MeOD): *δ* 7.82-7.81 (d, *J* = 7.6 Hz, 2H), 7.67-7.66 (d, *J* = 7.4 Hz, 2H), 7.42-7.31 (m, 4H), 5.80-5.75 (m, 1H), 5.01-4.93 (m, 2H), 4.40-4.39 (d, *J* = 6.7 Hz, 2H), 4.24-4.22 (t, *J* = 13.3 Hz, 1H), 2.41-2.36 (m, 1H), 2.27-2.19 (m, 1H), 2.14-2.00 (m, 6H), 1.37-1.21 (m, 5H).

^{13}C NMR (150 MHz, MeOD): *δ* 176.3, 156.2, 145.3, 142.6, 139.4, 129.9, 129.2, 128.8 (3C), 128.2 (2C), 126.3, 126.1 (2C), 121.0 (2C), 115.3, 67.2, 35.8, 35.8, 34.6, 29.5, 24.3, 15.4.

HR-Q-TOF-MS ***m/z*** calcd for $C_{25}H_{29}NO_4S$ 439.1821; found $[M+H]^+$ 440.1894; $[M+Na]^+$ 462.1714.

2.5.3 (S)-2-((((9H-fluoren-9-yl)methoxy)carbonyl)amino)-2-(tert-butoxymethyl)hept-6-enoic acid (1c)

This compound was prepared following the procedure described for **1a** (1.01 g, 20% over two steps) as white solid powder. $[\alpha]_D^{29}$= -0.7 (*c* 0.33, $CHCl_3$)

^{1}H NMR (600 MHz, MeOD): *δ* 7.80-7.79 (d, *J* = 7.7 Hz, 2H), 7.66-7.64 (t, *J* = 12.1 Hz, 2H), 7.40-7.29 (m, 4H), 5.79-5.75 (m, 1H), 5.00-4.92 (m, 2H), 4.33-4.21 (m, 3H), 3.87-3.84 (m, 1H), 3.65-3.63 (d, *J* = 8.9 Hz, 1H), 1.82-1.78 (m, 1H), 1.38-1.22 (m, 4H), 1.12-0.88 (m, 10H).

^{13}C NMR (150 MHz, MeOD): *δ* 174.0, 155.1, 144.0, 143.8, 141.2, 138.1, 132.6, 129.3, 128.1, 127.4 (2C), 126.8, 124.9, 124.8, 119.5, 113.8, 72.7, 66.2, 63.4, 62.5, 33.3, 31.0, 26.4 (3C), 22.7 (2C).

HR-Q-TOF-MS ***m/z*** calcd for $C_{27}H_{33}NO_5$ 451.2358; found $[M+H]^+$ 452.2436; $[M+Na]^+$ 474.2250.

2.5.4 (S)-2-((((9H-fluoren-9-yl)methoxy)carbonyl)amino)-2-(4-(tert-butoxy)benzyl)hept-6-enoic acid (1d)

This compound was prepared following the procedure described for **1a** (0.03 g, 3% over two steps) as white solid powder.

^{1}H NMR (600 MHz, $CDCl_3$): δ 7.81-7.80 (d, J = 7.4 Hz, 2H), 7.61-7.59 (m, 2H), 7.45-7.43 (t, J = 14.9 Hz, 2H), 7.36-7.33 (t, J = 15 Hz, 2H), 7.08-6.95 (m, 4H), 5.85-5.78 (m, 1H), 5.14-5.06 (m, 3H), 4.98-4.39 (m, 2H), 3.22-2.87 (m, 2H), 2.27-2.22 (m, 3H), 2.02-1.97 (m, 3H), 1.36 (s, 9H).

^{13}C NMR (150 MHz, $CDCl_3$): δ 175.6, 155.8, 154.5, 141.3, 136.7, 129.8 (2C), 127.8 (2C), 127.1 (2C), 125.1, 124.3 (2C), 120.0 (2C), 115.9 (2C), 67.1, 54.6, 47.2 (2C), 37.1, 33.1 (2C), 32.0 (2C), 31.8 (2C), 28.8 (4C).

HR-Q-TOF-MS ***m/z*** calcd for $C_{33}H_{37}NO_5$ 527.65; found $[M+H]^+$ 528.2654; $[M+Na]^+$ 550.2471.

2.5.5 (S)-2-((((9H-fluoren-9-yl)methoxy)carbonyl)amino)-2-(4-azidobutyl)hept-6-enoic acid (1e)

This compound was prepared following the procedure described for **1a** (0.75 g, 26% over two steps) as white solid powder. $[\alpha]_D^{29}$= -1.9 (*c* 0.33, $CHCl_3$)

^{1}H NMR (600 MHz, MeOD): δ 7.80-7.79 (m, 2H), 7.65-7.63 (m, 2H), 7.39-7.29 (m, 4H), 5.79-5.72 (m, 1H), 4.99-4.91 (m, 2H), 4.37-4.36 (d, J = 6.7 Hz, 2H), 4.22-4.20 (t, J = 13.4 Hz, 1H), 2.19-1.76 (m, 5H), 1.60-1.50 (m, 2H), 1.33-1.10 (m, 6H), 0.90-0.88 (m, 1H).

^{13}C NMR (151 MHz, MeOD): δ 176.6, 156.2, 145.3, 145.3, 142.6, 139.5, 128.8 (2C), 128.2 (2C), 126.2 (2C), 121.0 (2C), 115.3, 67.2, 64.2, 52.2, 35.6, 35.4, 34.7, 30.8, 29.8, 29.1, 24.37, 22.2.

HR-Q-TOF-MS ***m/z*** calcd for $C_{26}H_{30}N_4O_4$ 462.2271; found $[M+H]^+$ 463.2344; $[M+Na]^+$

485.2166.

2.5.6 (S)-2-((((9H-fluoren-9-yl)methoxy)carbonyl)amino)-2-(3-azidopropyl)hept-6-enoic acid (7f)

This compound was prepared following the procedure described for **1a** (0.31 g, 30% over two steps) as white solid powder.

^{1}H NMR (600 MHz, MeOD): *δ* 7.80-7.78 (m, 2H), 7.64-7.63 (d, *J* = 7.4 Hz, 2H), 7.39-7.30 (m, 4H), 5.78-5.73 (m, 1H), 4.99-4.91 (m, 3H), 4.39-4.38 (d, *J* = 6.5 Hz, 1H), 4.21-4.21 (m, 1H), 2.14-1.46 (m, 6H), 1.47-1.20 (m, 6H).

^{13}C NMR (150 MHz, MeOD): *δ* 175.0, 174.6, 173.4, 154.8, 143.9, 141.3, 138.0, 127.4, 126.8, 124.7, 123.9, 119.5, 113.9, 65.8, 62.6, 50.9, 50.8, 34.2, 33.2, 31.7, 29.3, 28.4, 28.3, 23.2, 23.0.

HR-Q-TOF-MS ***m/z*** calcd for $C_{25}H_{28}N_4O_4$ 448.51; found $[M+H]^+$ 449.2097; $[M+Na]^+$ 471.1916.

2.5.7 (S)-2-((((9H-fluoren-9-yl)methoxy)carbonyl)amino)-2-benzylhept-6-enoic acid (1g)

This compound was prepared following the procedure described for **1a** (1.75 g, 33% over two steps, 95.82:4.18 er) as white solid powder. $[\alpha]_D^{29}$= +23.8 (*c* 0.33, $CHCl_3$)

^{1}H-NMR (400 MHz, $CDCl_3$): *δ* 7.85-7.75 (d, *J* = 7.6 Hz, 2H,), 7.66-7.56 (m, 2H), 7.50-7.39 (t, *J* = 7.2 Hz, 2H,), 7.40-7.30 (m, 2H), 7.27-7.16 (m, 3H), 5.90-5.70 (m, 1H), 5.57 (s, 1H), 5.13-4.87 (m, 2H), 4.66-4.49 (m, 1H), 4.50-4.34 (m, 1H), 4.33-4.23 (t, *J* = 6.4 Hz, 1H), 3.67-3.56 (d, *J* = 13.2 Hz, 1H), 3.26-3.07 (d, *J* = 13.2 Hz, 1H), 2.62-2.40 (m, 1H), 2.20-2.02 (m, 2H), 2.01-1.88 (t, *J* = 11.2 Hz, 1H), 1.57-1.41 (m, 1H), 1.40-1.14 (m, 3H).

^{13}C-NMR (400 MHz, $CDCl_3$): *δ* 177.9, 154.3, 143.9, 143.8, 141.4, 138.0, 136.0, 129.8, 128.4, 127.7, 127.1, 127.0, 125.1, 125.0, 120.0, 115.1, 66.4, 65.0, 47.3, 40.8, 35.2, 33.4, 23.4.

HR-Q-TOF-MS ***m/z*** calcd for $C_{29}H_{29}NO_4$ 455.2; found $[M+H]^+$ 456.2093; $[M+Na]^+$ 478.1914.

2.5.8 (S)-2-((((9H-fluoren-9-yl)methoxy)carbonyl)amino)-2-(3-(2,3-bis(tert-butoxy carbonyl)guanidino)propyl)hept-6-enoic acid (1f)

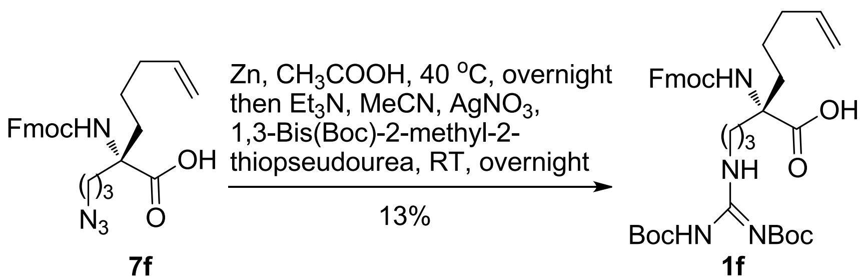

Zn (1.3 g, 20.16 mmol) was added into a solution of **7f** (502 mg, 1.12 mmol) in CH_3COOH (20 mL) at 40 °C. The reaction mixture was stirred at 40 °C overnight and the reaction mixture was concentrated *in vacuo*. The residue was suspended in a mixture of 1,3-Bis(tert-butoxycarbonyl)-2-methyl-2-thiopseudour-ea (511 mg, 1.76 mmol), Et_3N (193 mg, 1.9 mmol) and MeCN (15 mL), $AgNO_3$(273 mg, 1.61 mmol) were dissolved in MeCN (8 mL) and the solution was added with efficient stirring at room temperature overnight in the dark, the resultant precipitate was filtered off and the filtrate was concentrated to dryness. The residue was dissolved in 20 mL of EA and then washed with brine. The organic layer was dried and concentrated. The residue was purified by column chromatography eluting with DCM/MeOH (100:1) to yield **1f** (96 mg, 12.85 %) as white solid powder.

^{1}H NMR (600 MHz, $CDCl_3$): δ 7.76-7.74 (t, J = 12.4 Hz, 2H), 7.60-7.58 (m, 2H), 7.39-7.38 (m, 2H), 7.32-7.30 (m, 2H), 5.77-5.67 (m, 1H), 5.02-4.94 (m, 2H), 4.45-4.34 (m, 2H), 4.22-4.20 (m, 1H), 3.43-3.20 (m, 2H), 2.34-2.10 (m, 2H), 2.05-1.98 (m, 4H), 1.63-1.62 (m, 1H), 1.53-1.18 (m, 22H).

^{13}C NMR (150 MHz, $CDCl_3$): δ 144.0, 144.0, 143.9, 143.8, 141.4, 141.3, 138.0, 137.8, 127.7, 127.6, 127.1, 127.1, 124.9, 120.0, 119.9, 115.3, 115.2, 83.1, 82.8, 66.4, 50.0, 49.9, 49.7, 47.2, 47.2, 36.4, 33.6, 33.2, 30.8, 29.7, 28.0, 28.0, 24.0, 22.8, 22.7, 22.6.

HR-Q-TOF-MS *m/z* calcd for $C_{36}H_{48}N_4O_8$ 664.3472; found $[M+Na]^+$ 687.3367; $[M+K]^+$ 703.3092.

2.5.9 (R)-2-((((9H-fluoren-9-yl)methoxy)carbonyl)amino)-2-benzylhept-6-enoic acid (1g`)

This compound was prepared following the procedure described for **1g** (0.134 g, 29% over two steps) as white solid powder.

3. Synthesis and characterization of *α*-helix stapled, side chains retention peptides

3.1 Reduction of azides on the peptide-resin

After two rounds of RCM, the peptide-resin was washed with DMF (5 times) and DCM (5 times). The peptide-resin were dissolved in solution (72 mL/g) of DCM/EtOH (1:1), then the $NaBH_4$ (2 eq.) and $NiNO_2{\cdot}6H_2O$ (0.02 eq.) were added, the reaction mixture was stirred for 2 hrs at 40 °C, After 2 hrs, the resin was washed with DMF (5 times) and DCM (5 times). The next operation was prepared following the procedure described for section **1.6**.

3.2 HR-Q-TOF-MS of peptides

Table S1. Electrospray MS data for peptides (positive mode)

Peptide	Exact Mass	Found Mass	Yield
Axin(469-482)	1720.89	$[M+2H]^{2+}$ = 861.4559; $[M+3H]^{3+}$ = 574.6406	139 mg, 72.12%
WNT-1a	1770.98	$[M+2H]^{2+}$ = 887.0020; $[M+3H]^{3+}$ = 591.6709	12 mg, 6.05 mg
WNT-2d	1828.99	$[M+2H]^{2+}$ = 916.0041; $[M+3H]^{3+}$ = 611.0062	6 mg, 2.93%
WNT-3a	1800.96	$[M+2H]^{2+}$ = 901.4898; $[M+3H]^{3+}$ = 601.3290	31 mg, 15.37%
WNT-3b	1740.95	$[M+2H]^{2+}$ = 871.4861; $[M+3H]^{3+}$ = 581.3269	47 mg, 24.10%
WNT-3c	1740.95	$[M+2H]^{2+}$ = 871.4864; $[M+3H]^{3+}$ = 581.3271	91 mg, 46.67%
WNT-3d	1828.99	$[M+2H]^{2+}$ = 915.5015; $[M+3H]^{3+}$ = 610.6702	64 mg, 41.66%
WNT-4a	1771.97	$[M+2H]^{2+}$ = 887.4937; $[M+3H]^{3+}$ = 591.9993	4 mg, 2.02%
WNT-5a	1785.00	$[M+2H]^{2+}$ = 894.0078; $[M+3H]^{3+}$ = 596.0075	34 mg, 17.01%
WNT-5b	1769.00	$[M+2H]^{2+}$ = 886.0122; $[M+3H]^{3+}$ = 591.0111	56 mg, 28.26%
WNT-6a	1762.97	$[M+2H]^{2+}$ = 882.4918	17 mg, 8.61%
WNT-6b	1720.92	$[M+2H]^{2+}$ = 861.4689	23 mg, 11.93%

WNT-7d	1800.00	$[M+2H]^{2+}$ = 901.5114; $[M+3H]^{3+}$ = 601.3442	5 mg, 2.48%
WNT-8a	1743.92	$[M+2H]^{2+}$ = 872.9700	4 mg, 2.05%
FITC-β-Ala-Axin (469-482)	2138.95	$[M+2H]^{2+}$ = 1070.9874; $[M+3H]^{3+}$ = 714.3288	86 mg, 35.90%
FITC-β-Ala-WNT-3a	2219.02	$[M+2H]^{2+}$ = 1111.0195; $[M+3H]^{3+}$ = 741.016; $[M+4H]^{4+}$ = 556.0150	19 mg, 7.64%
FITC-β-Ala-WNT-3c	2159.02	$[M+2H]^{2+}$ = 1081.0177; $[M+3H]^{3+}$ = 721.0143	75 mg, 31.02%

3.3 Coupling efficiency of N-Fmoc-α-pentene amino acids and the subsequent amino acid

As a typical example, 100 mg Rink Amide MBHA resin was swelled with DCM (2 mL) for 20 mins. Then the resin was treated with 20% piperidine/DMF twice (10 and 5 mins), followed by washing with DMF (5 times), DCM (5 times) and DMF (5 times). For coupling of the first amino acid, Fmoc-Lys (Boc)-OH (1 mmol), HCTU (0.9 mmol), DIEA (2 mmol) and DMF (6 mL) were mixed for 2 mins and then added to the resin. After 2 hrs, the resin was washed with DMF (5 times), DCM (5 times), and DMF (5 times). Then the resin was treated with 20% piperidine/DMF twice (10 and 5 mins), followed by washing with DMF (5 times), DCM (5 times) and DMF (5 times). About a quarter of resin was cleavaged by treatment with reagent K for 1 hr at room temperature to yeild **WNT-3d-1**. The coupling of Fmoc-Met-OH* (**1b**) was carried out over 2 hrs, and then one third of resin was cleavage to produce **WNT-3d-2**. Then the resin was treated with 20% piperidine/DMF twice (10 and 5 mins), followed by washing with DMF (5 times), DCM (5 times) and DMF (5 times). **WNT-3d-3 was obtained by the cleavage of** the half of resultant resin. In the end, the couplings of Fmoc-Val-OH was performed over 1 hr, and **WNT-3d-4** was cleaved from resin. The four samples were monitored by HPLC to determine the coupling efficiency of N-Fmoc-α-pentene amino acids and the subsequent amino acid (Figure S1, A linear gradient of 1% to 1% B over 3 mins, then a linear gradient of 1% to 40% B over 30 mins.), **ESI-MS** of **WNT-3d-1** ***m/z*** calcd for $C_6H_{15}N_3O$

145.12; found $[M+H]^+$ = 146.18, **ESI-MS** of **WNT-3d-2** ***m/z*** calcd for $C_{31}H_{42}N_4OS$ 556.29; found $[M+H]^+$ = 567.25, **ESI-MS** of **WNT-3d-3** ***m/z*** calcd for $C_{16}H_{32}N_4O_2S$ 344.22; found $[M+H]^+$ = 345.21, **ESI-MS** of **WNT-3d-4** ***m/z*** calcd for $C_{36}H_{51}N_5O_5S$ 665.36; found $[M+H]^+$ = 666.42.

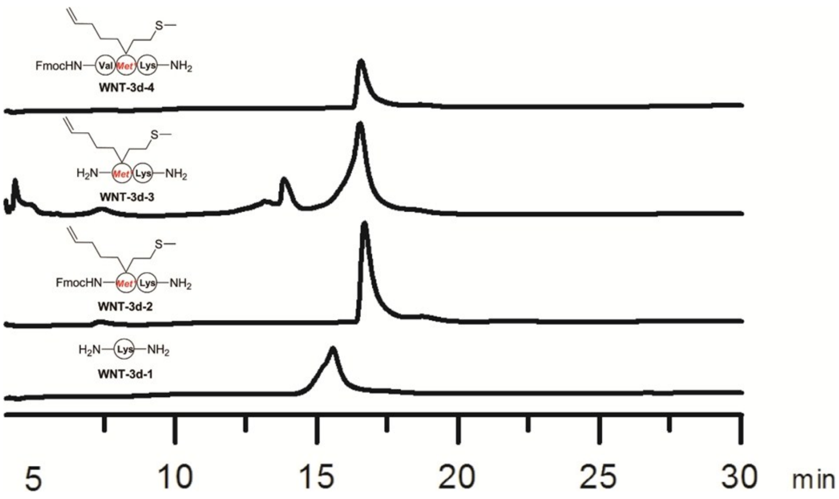

Figure S1. The analytical HPLC (λ=214 nm) of **WNT-3d-1~4**

3.4 Topflash reporter assay

We performed the Topflash reporter assay in which the firefly luciferase is transcriptionally activated by β-catenin[2].

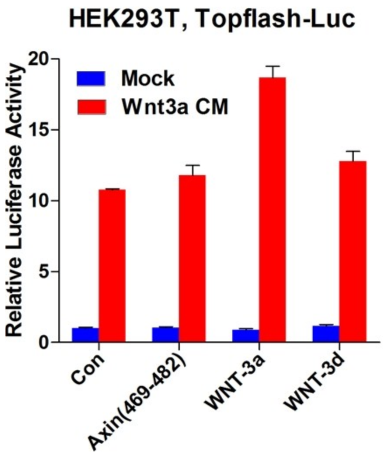

Figure S2. HEK293T cells transfected with Topflash-luciferase were treated with Wnt3a conditioned medium (CM) and target peptides (Axin (469-482), WNT-**3a**, WNT-**3d**, 40 μM) at the indicated concentrations for 12 h and then harvested for luciferase measurement.

3.5 Circular Dichroism

The linear and stapled peptides were dissolved in the phosphate buffer solutions (10 mM, pH = 7.4) to a final concentration of 50 μM, respectively. The CD spectra were obtained with 1 mm quartz cuvette on Jasco-715 spectropolarimeter at 25 ^{o}C. The measurement parameters were set up as follows: wavelength, 185-260 nm; step resolution, 0.5 nm; speed, 20 nm min^{-1}; accumulation, 3. All spectra were converted to a uniform scale of molar ellipticity after background subtraction. The curves were smoothed using standard parameters. In addition, according to the ellipticity of the peptide's spectrum at 222 nm and the number of amino acids in the peptide sequence, we calculated the helicity of each peptide using the literature equation[3].

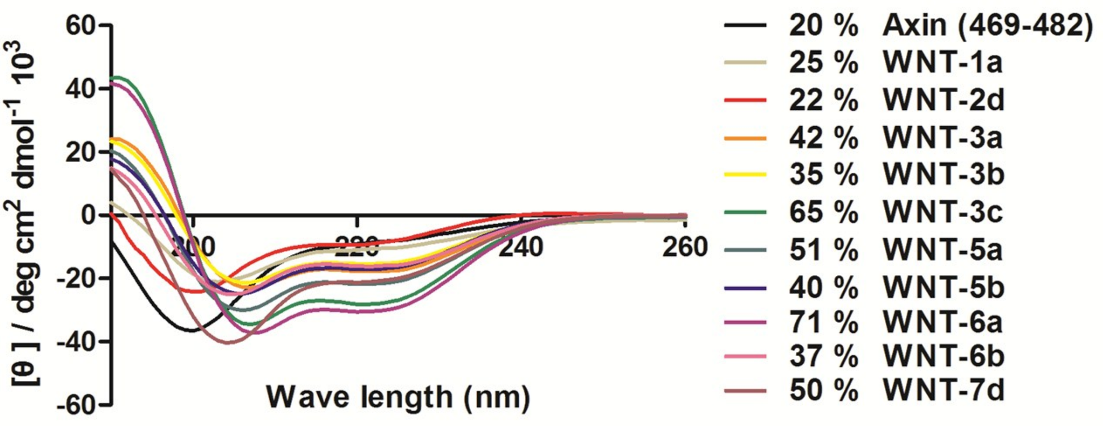

Figure S3. CD spectra of all peptides. The peptides were dissolved in the PBS buffer at a final concentration of 50 μM. The percent helicity was calculated based on the $[\theta]_{222}$ value.

4. Cell permeability

HEK293T Cells were incubated with FITC-β-Ala-Axin (469-482), FITC-β-Ala -WNT-3a and FITC-β-Ala-WNT-3c (10 μM) for 16 hrs and then washed with PBS twice before fixation for confocal microscopy.

5. Protease stability

The Axin(469-482) and WNT-3a were dissolved in PBS buffer solutions (50 mM, pH = 7.4) to a final concentration of 1mM, respectively. α-Chymotrypsin was dissolved in PBS buffer (50 mM, containing 2 mM of $CaCl_2$, pH = 7.4) to a final concentration of 0.5 ng/μL. Then the peptide solutions (100 μL) were incubated with α-Chymotrypsin solution (1 mL) at room temperature. 100 μL of digestion mixture was taken at the 0, 5, 10, 20, 30, 40, and 60

minute marks, then quenched with 20 μL of hydrochloric acid (1 M). The solution of the α-Chymotrypsin peptide fragments was monitored by HPLC at different times to determine the fraction of protease degradation at 214 nm. Each experiment was performed in duplicate.

6. Spectrum

^{1}H-NMR of compound 2e-2 (MeOD)

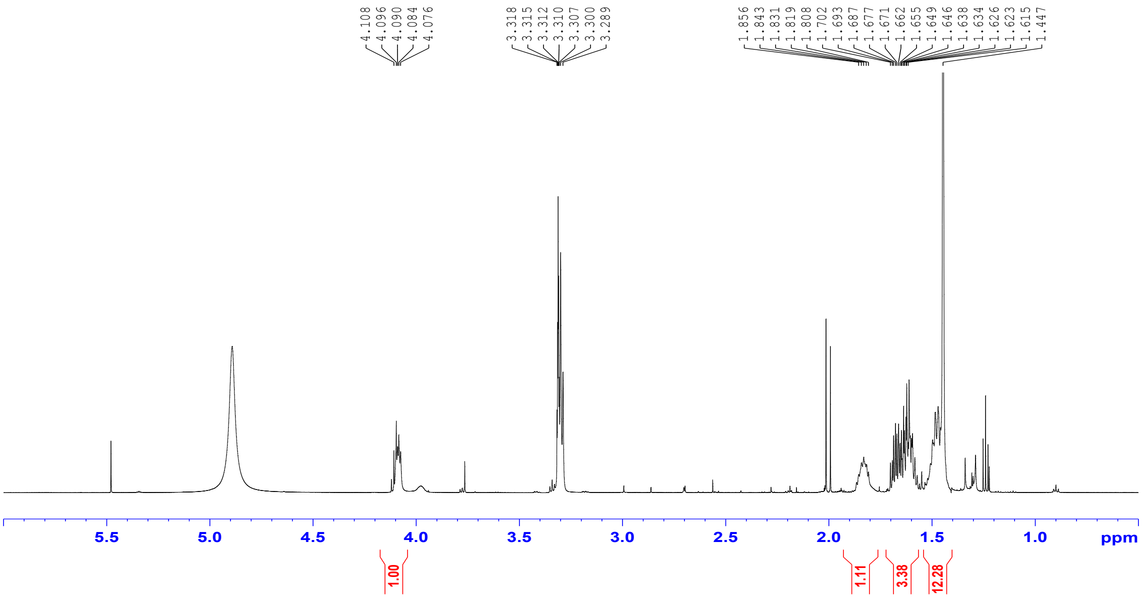

^{13}C-NMR of compound 2e-2 (MeOD)

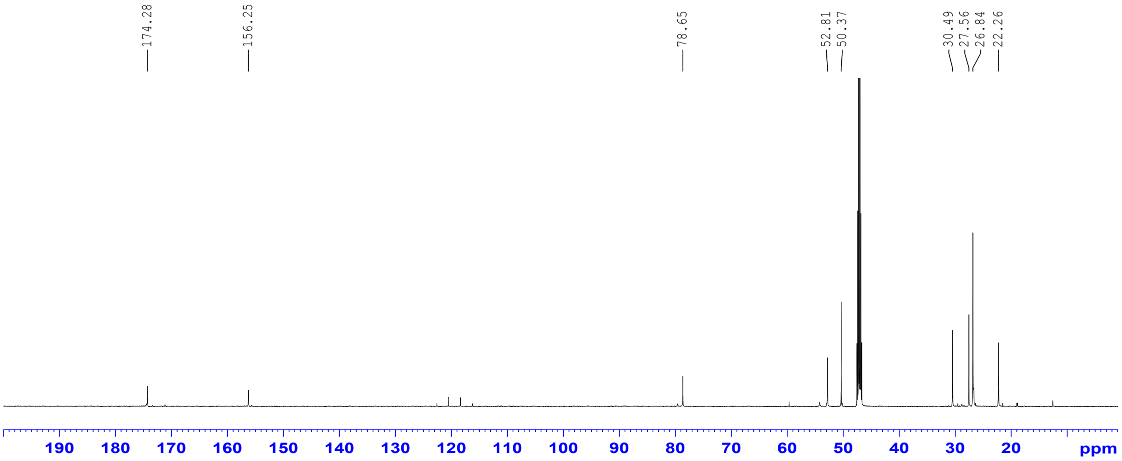

HR-Q-TOF-MS of compound 2e-2

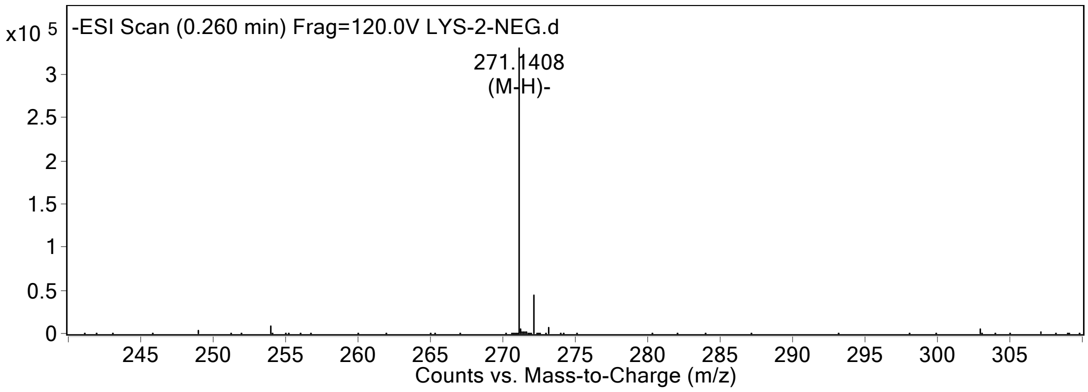

^{1}H-NMR of compound 2f-2 (MeOD)

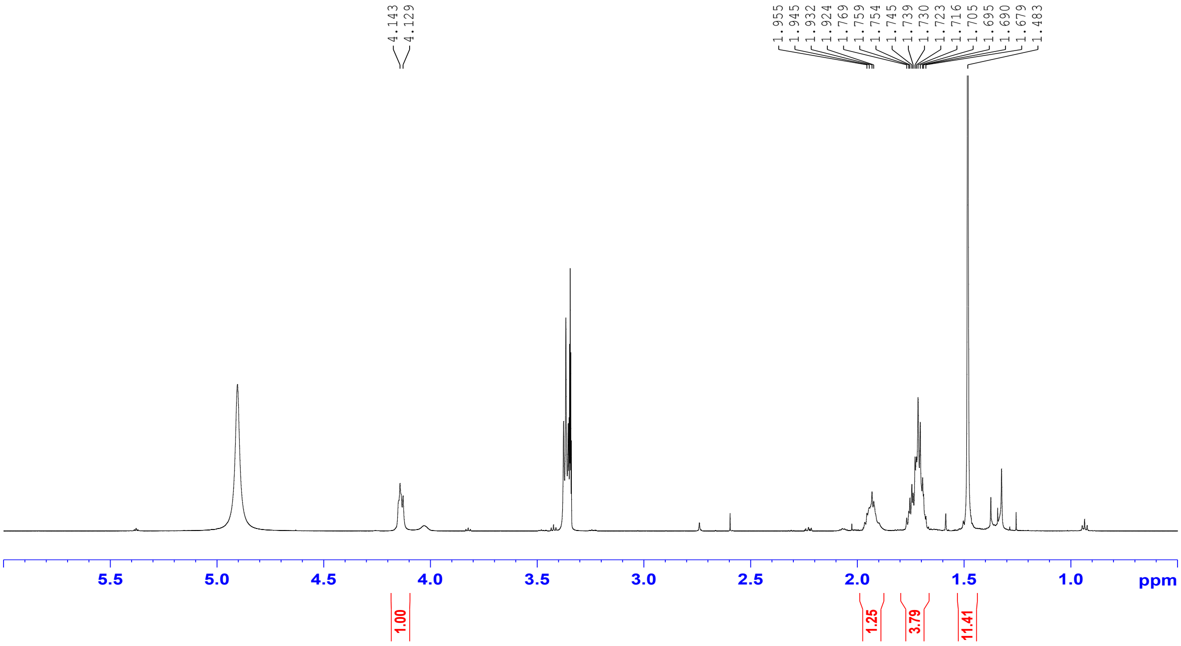

^{13}C-NMR of compound 2f-2 (MeOD)

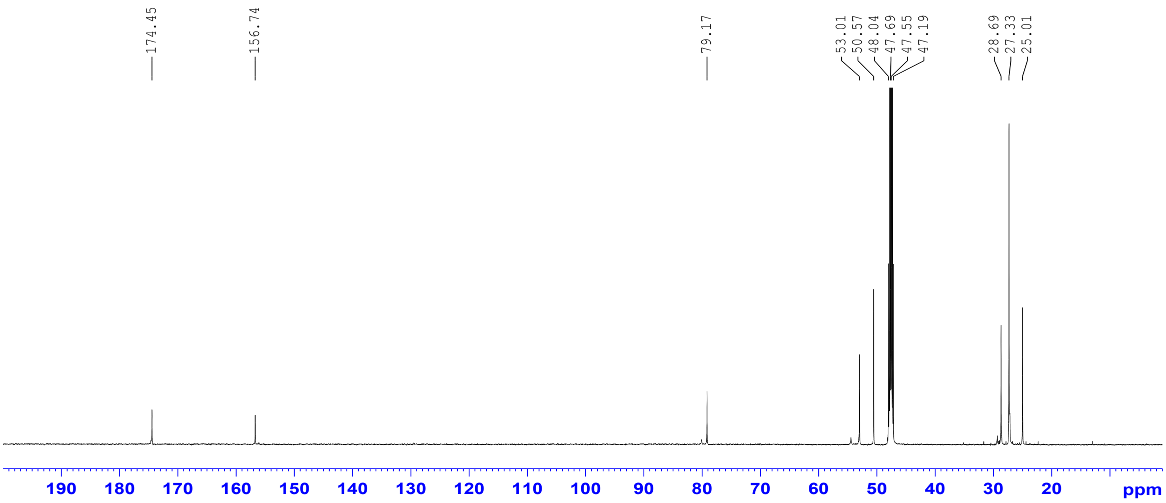

HR-Q-TOF-MS of compound 2f-2

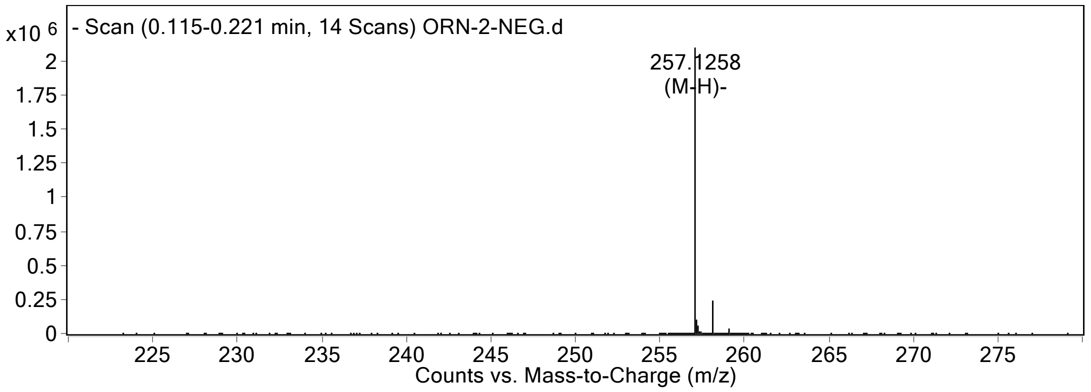

IR of compound 3a (KBr)

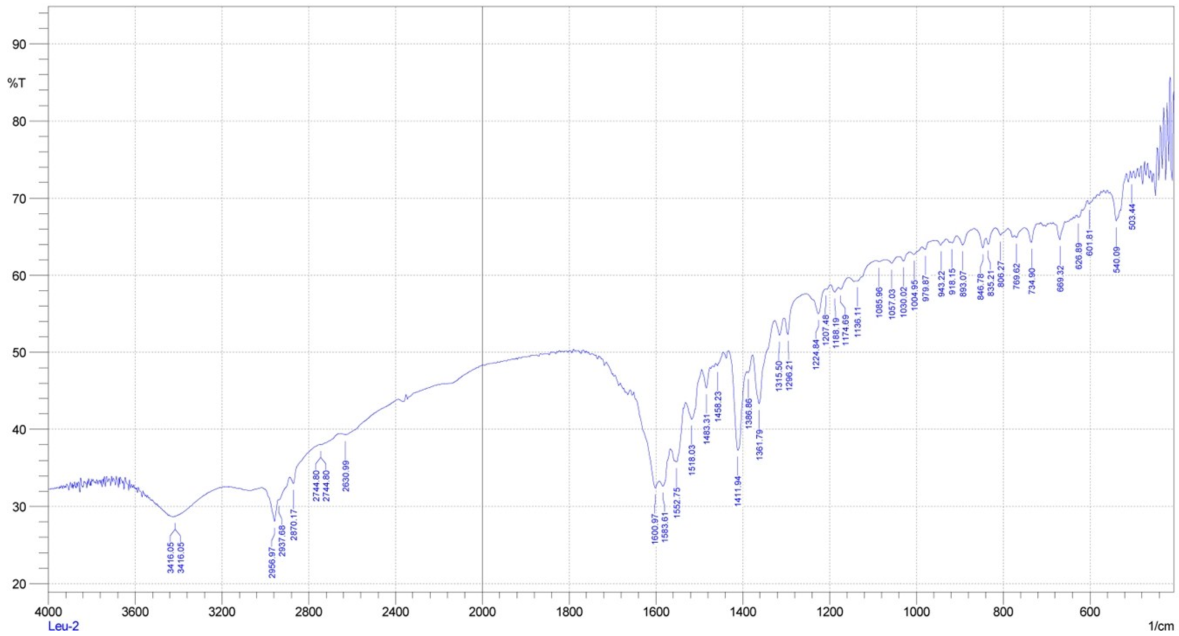

IR of compound 3b (KBr)

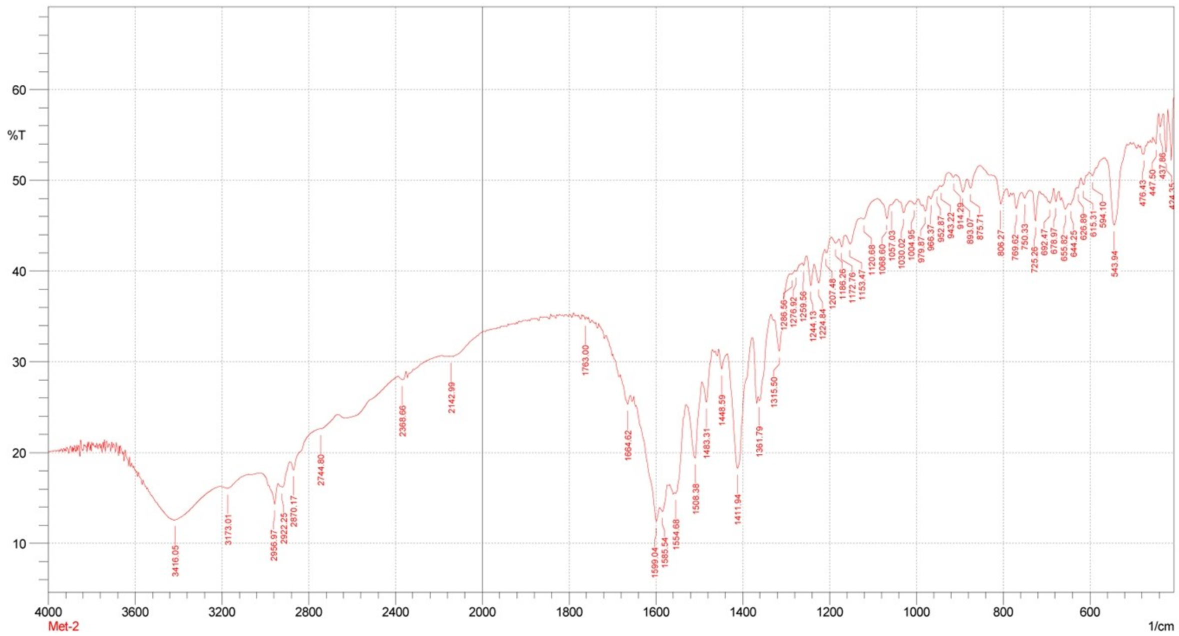

^{1}H-NMR of compound 4a ($CDCl_3$)

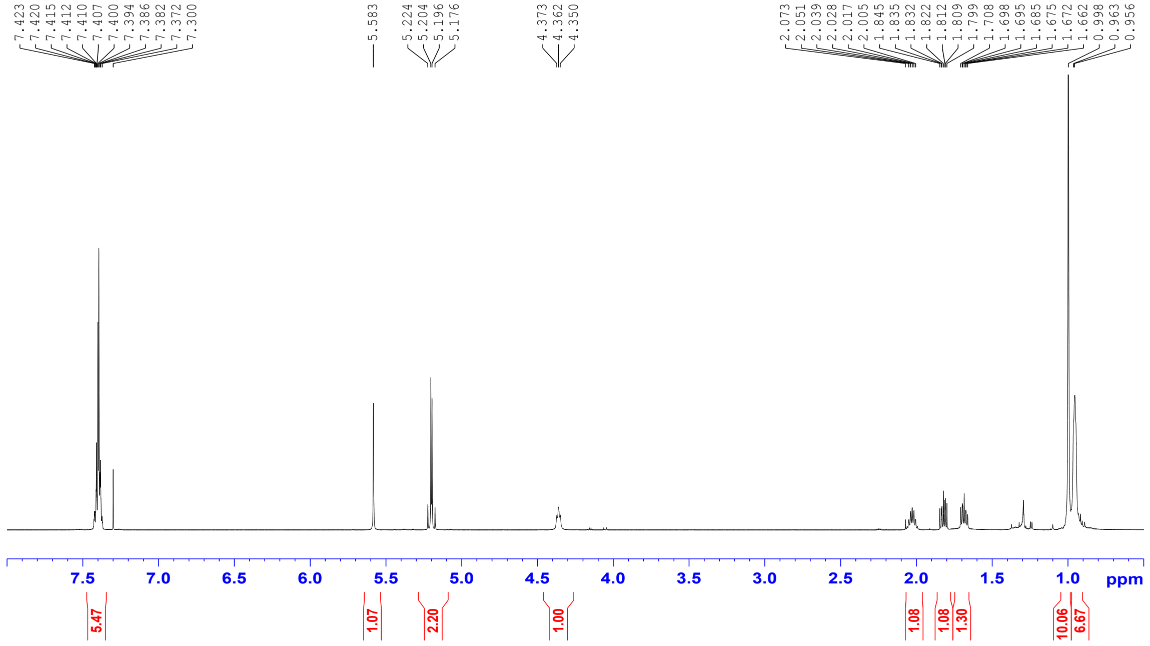

^{13}C-NMR of compound 4a ($CDCl_3$)

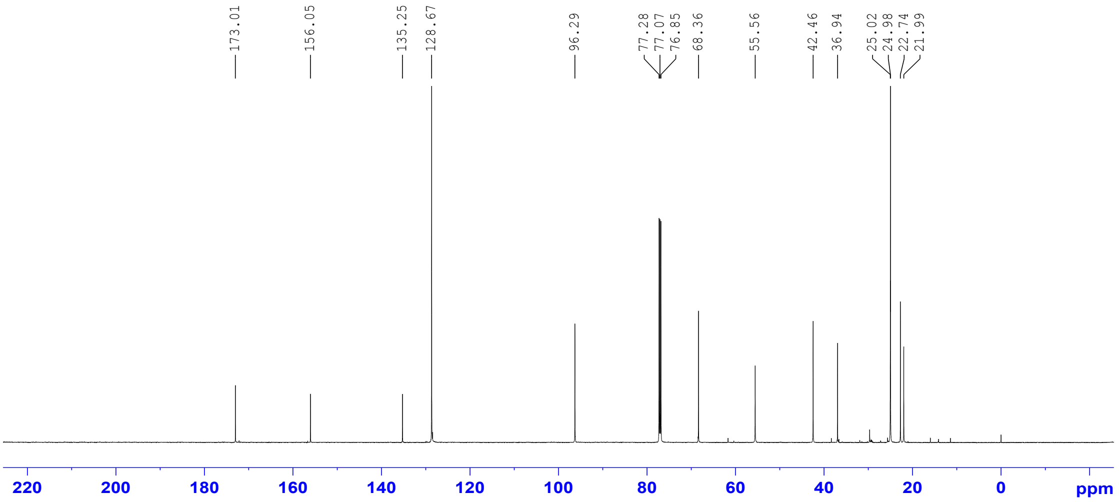

HR-Q-TOF-MS of compound 4a

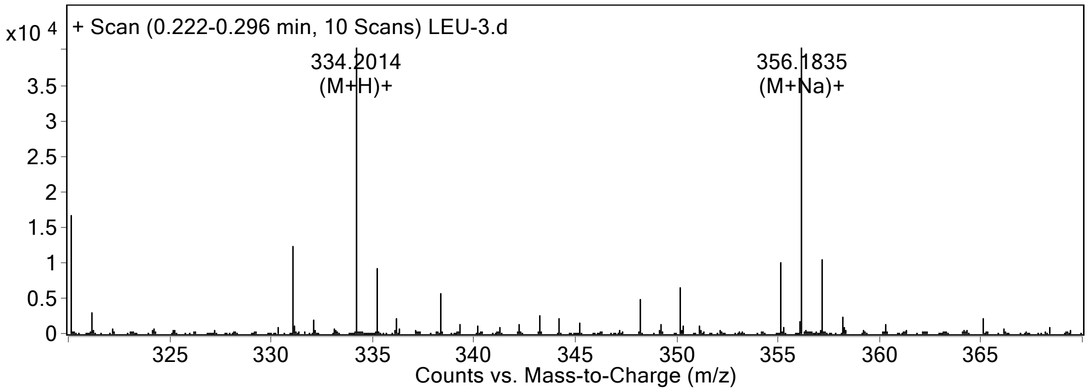

^{1}H-NMR of compound 4b ($CDCl_3$)

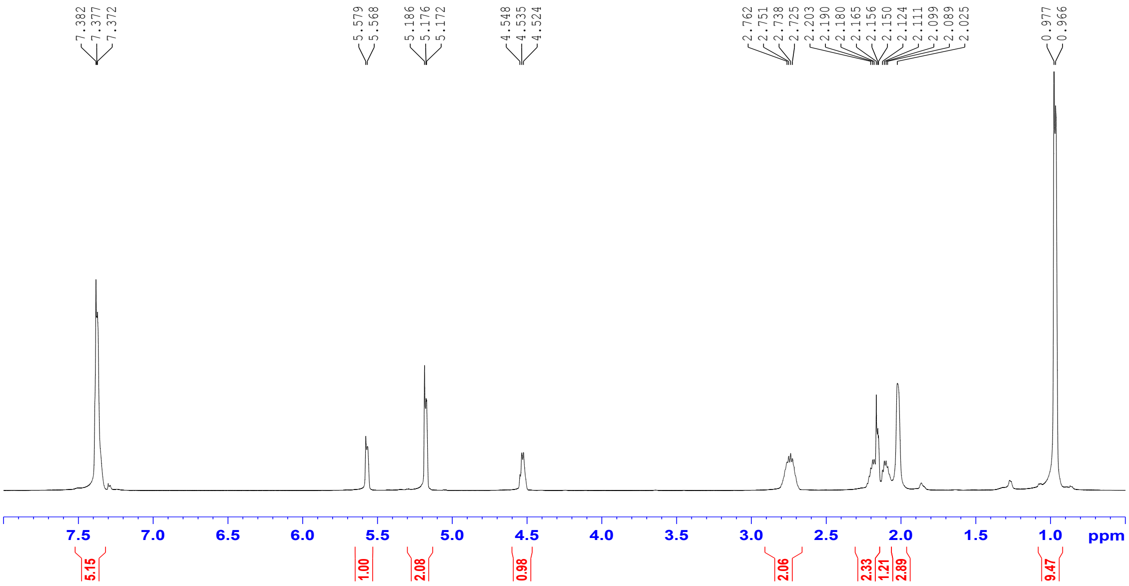

^{13}C-NMR of compound 4b ($CDCl_3$)

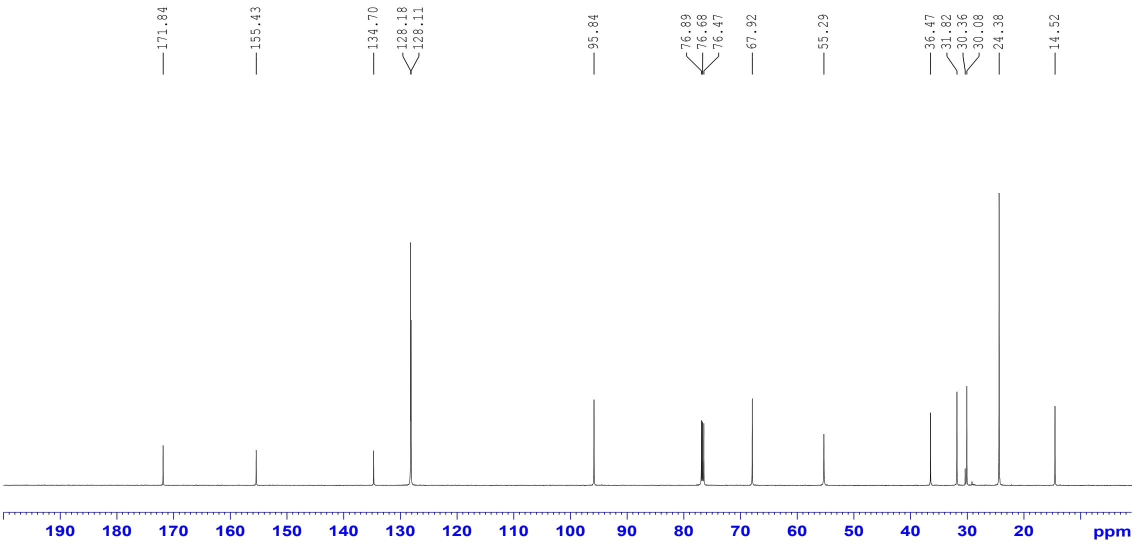

HR-Q-TOF-MS of compound 4b

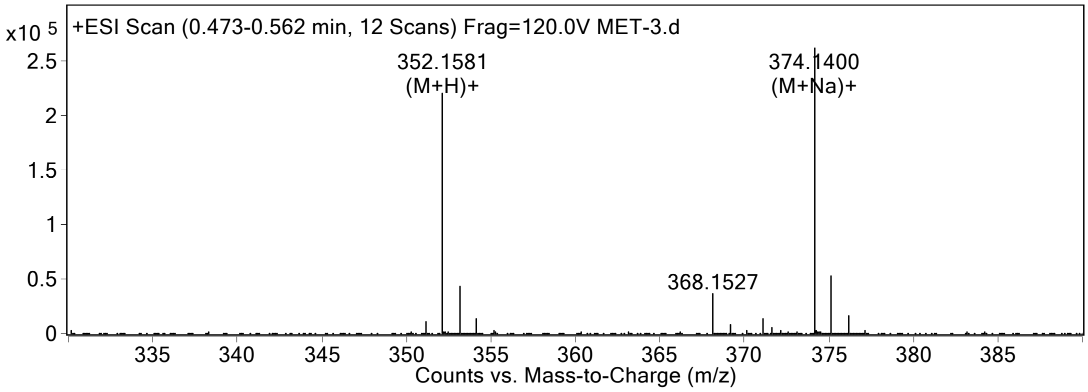

NOEs of compound 4b

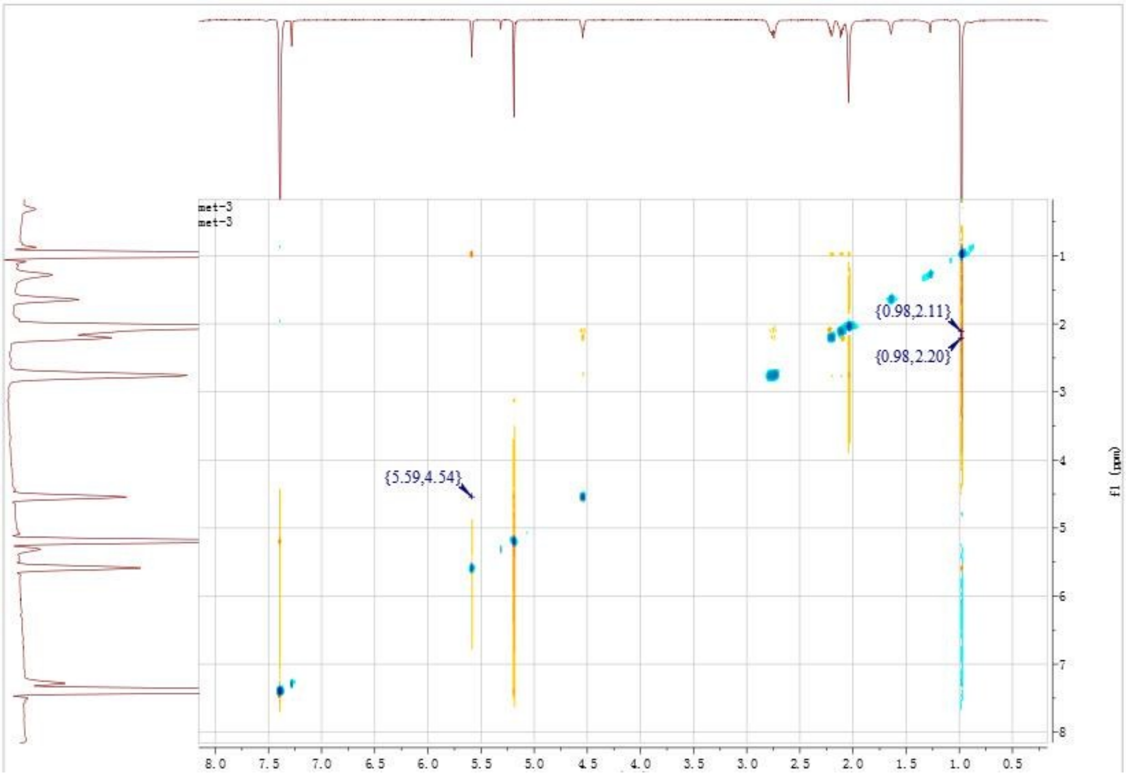

^{1}H-NMR of compound 4c ($CDCl_3$)

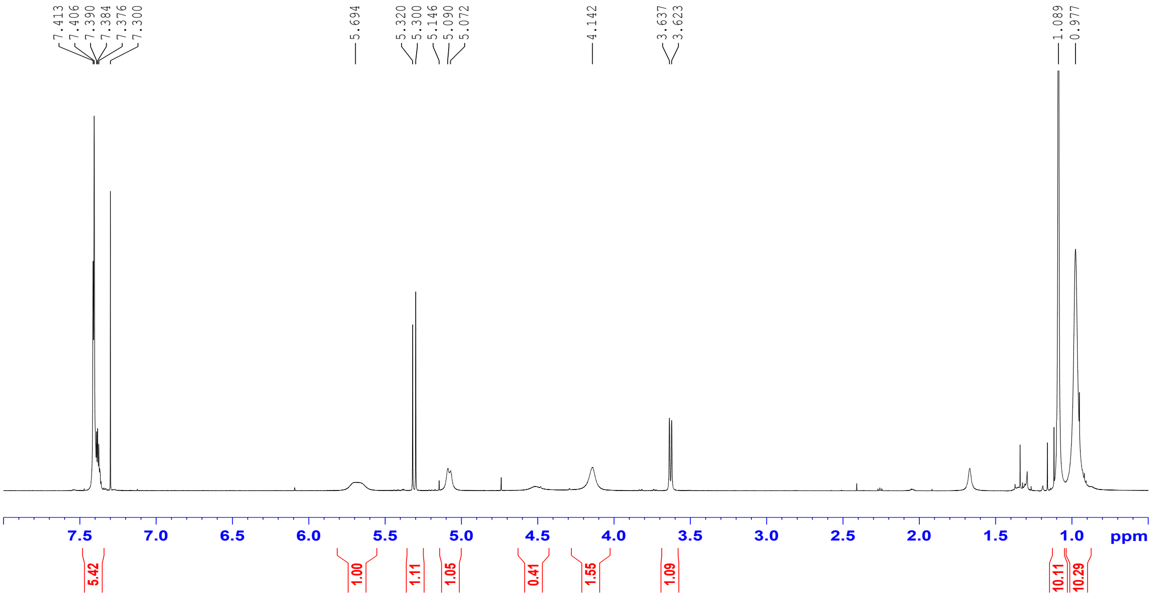

^{13}C-NMR of compound 4c ($CDCl_3$)

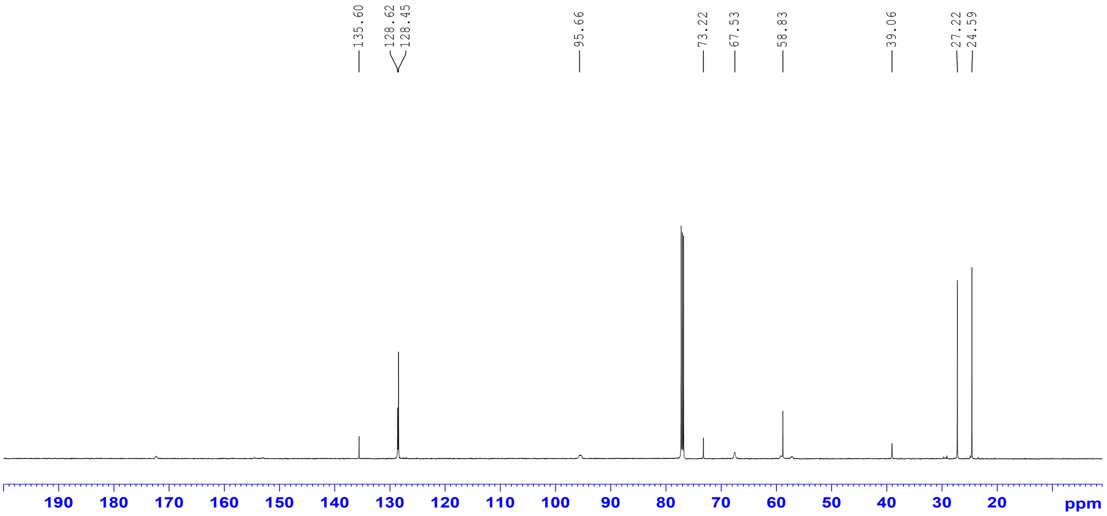

HR-Q-TOF-MS of compound 4c

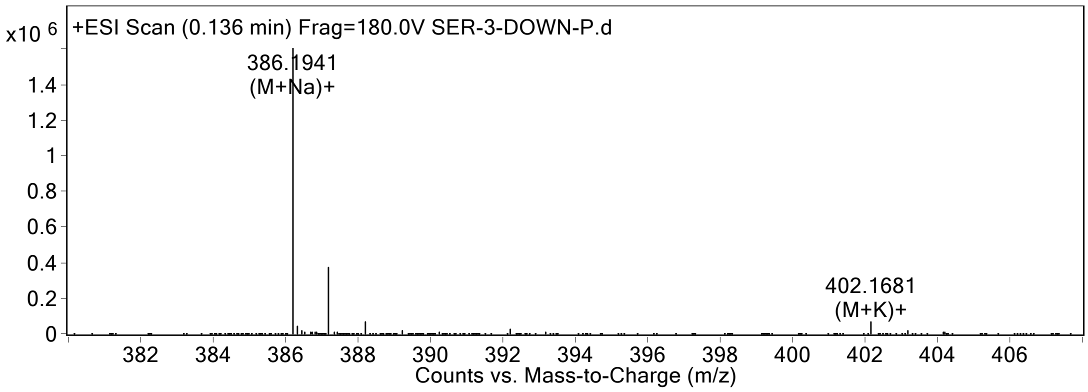

^{1}H-NMR of compound 4d ($CDCl_3$)

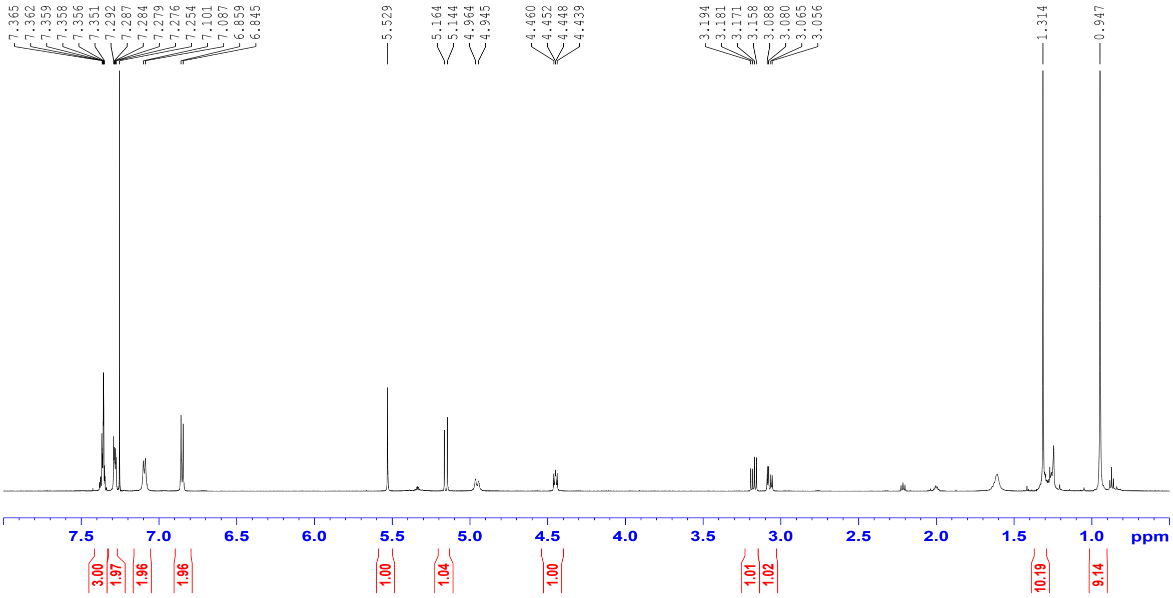

^{13}C-NMR of compound 4d ($CDCl_3$)

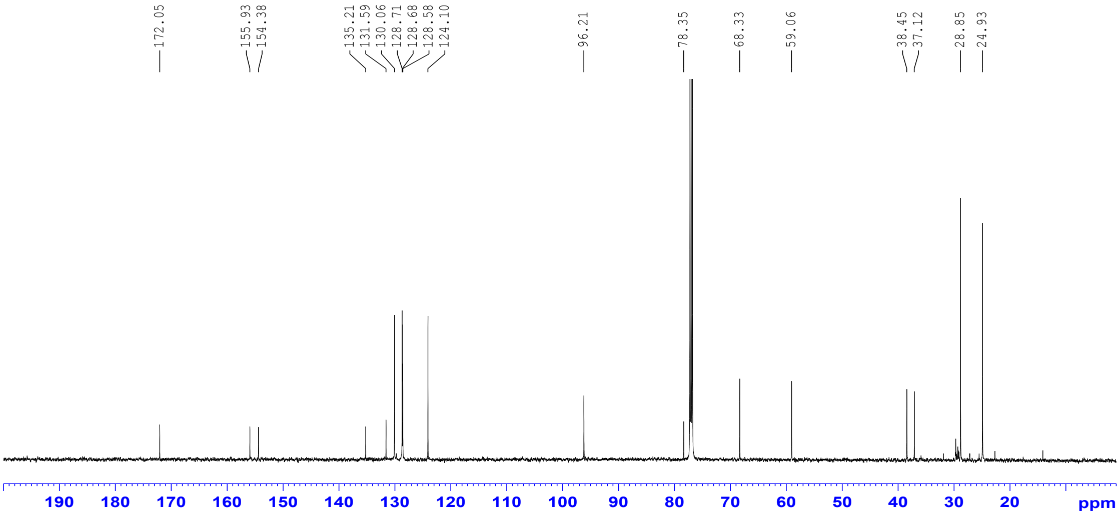

HR-Q-TOF-MS of compound 4d

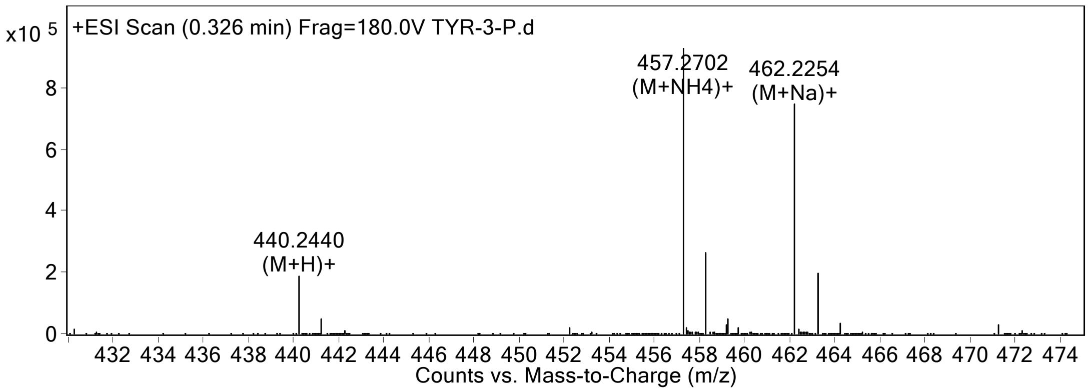

^{1}H-NMR of compound 4e ($CDCl_3$)

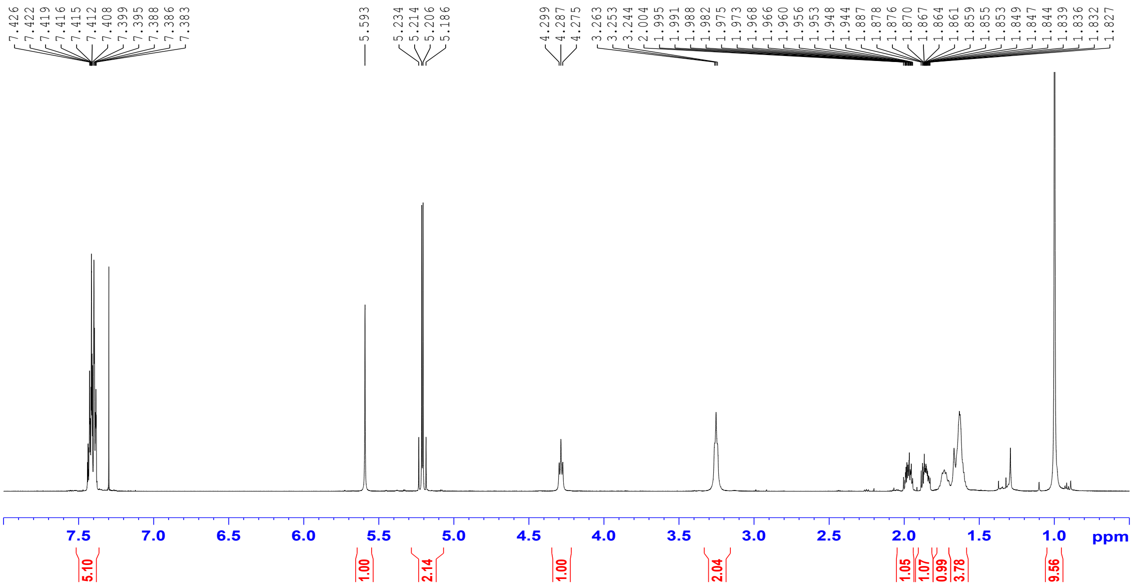

^{13}C-NMR of compound 4e ($CDCl_3$)

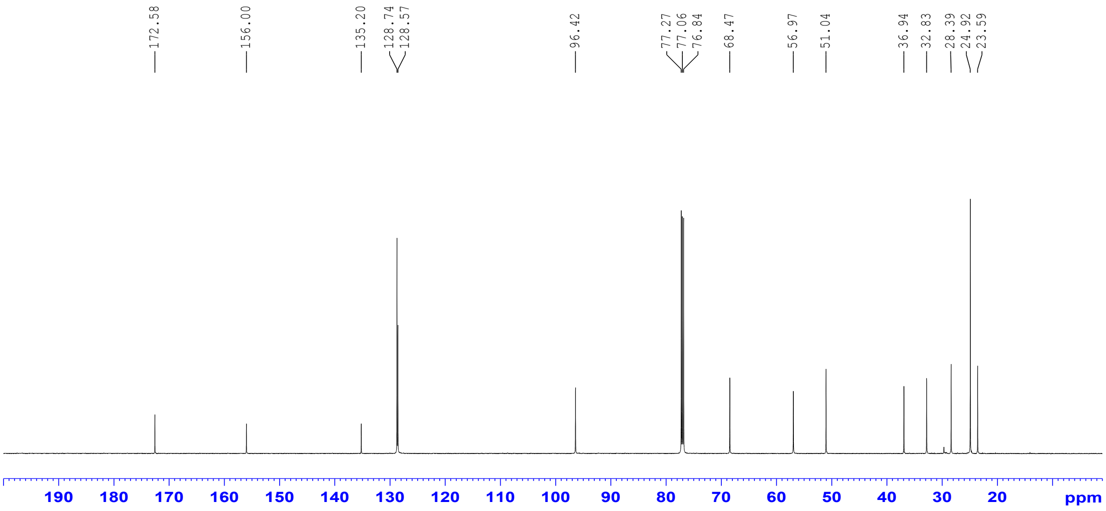

HR-Q-TOF-MS of compound 4e

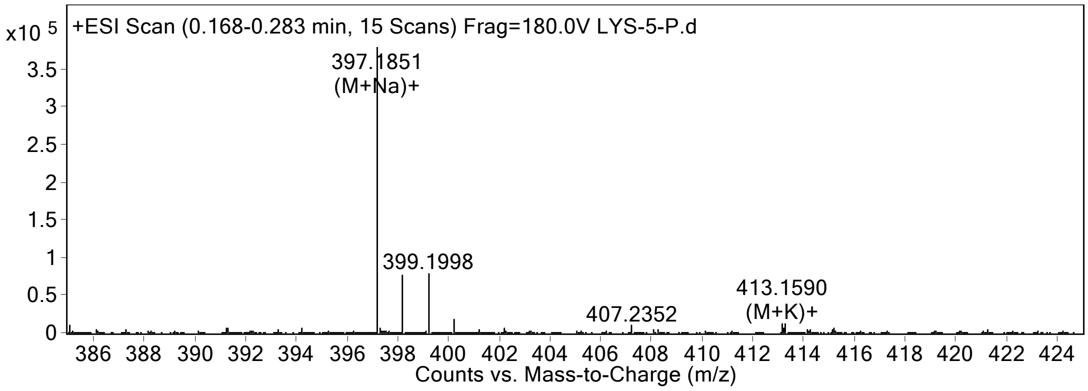

^{1}H-NMR of compound 4f ($CDCl_3$)

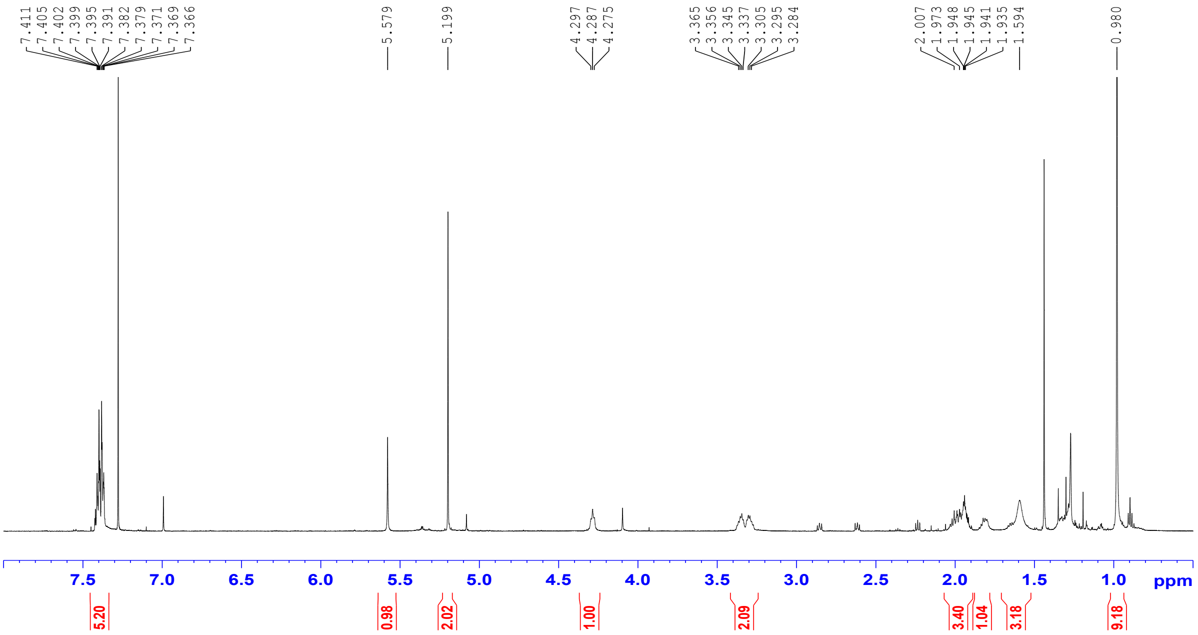

^{13}C-NMR of compound 4f ($CDCl_3$)

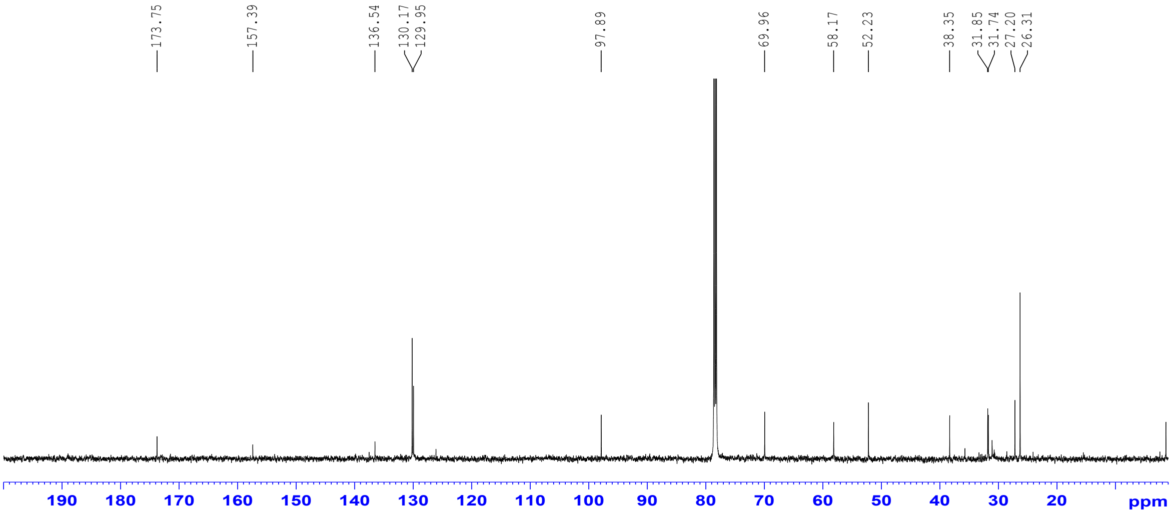

HR-Q-TOF-MS of compound 4f

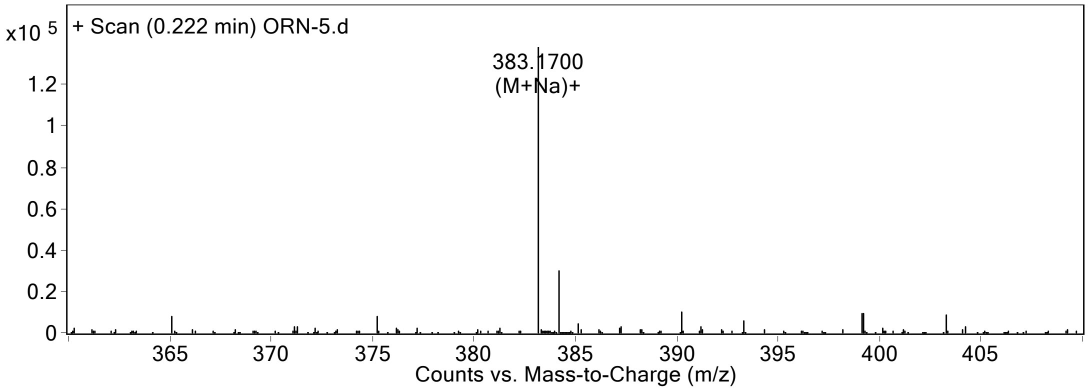

^{1}H-NMR of compound 4g ($CDCl_3$)

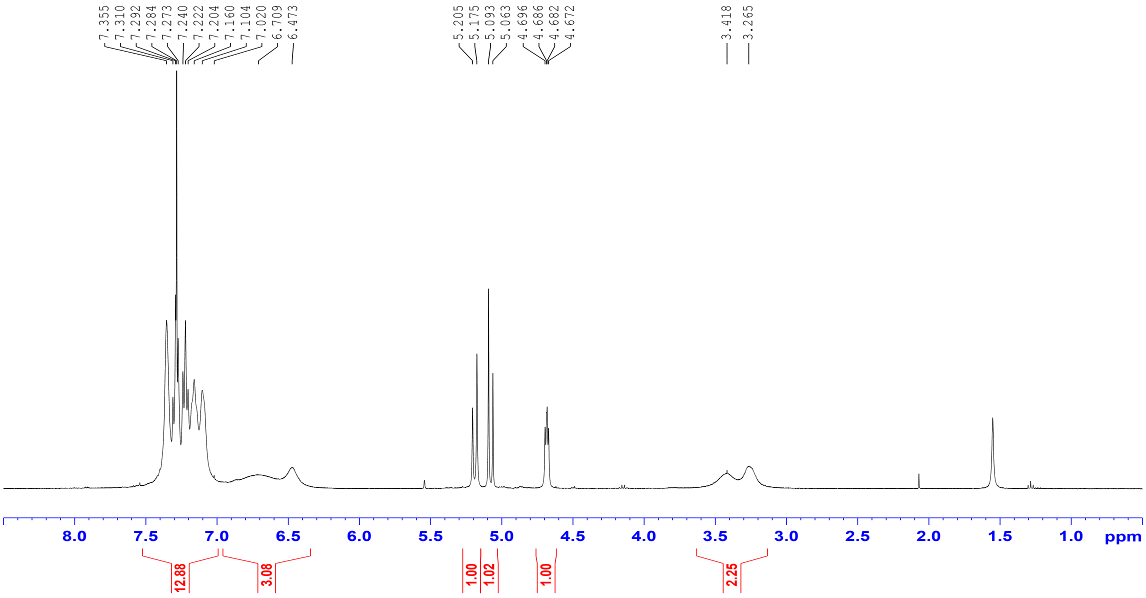

^{13}C-NMR of compound 4g ($CDCl_3$)

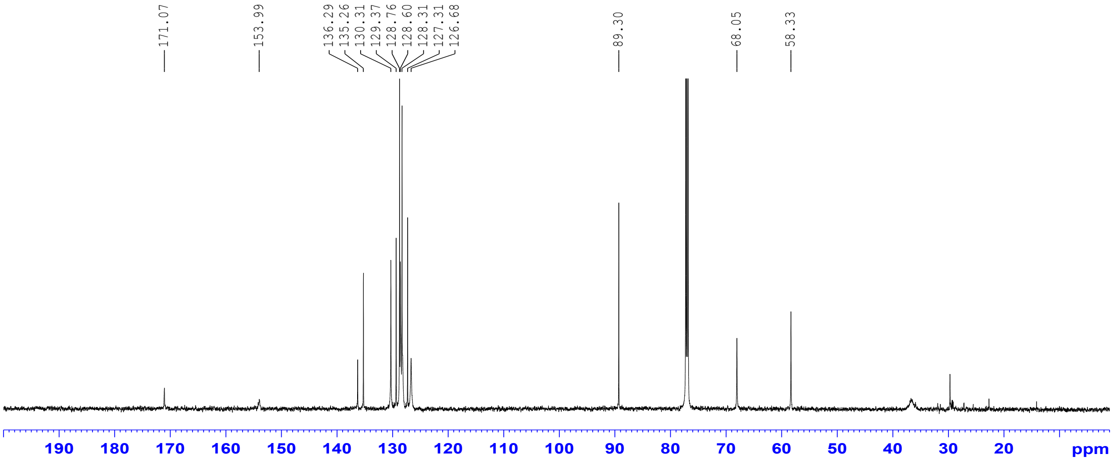

HR-Q-TOF-MS of compound 4g

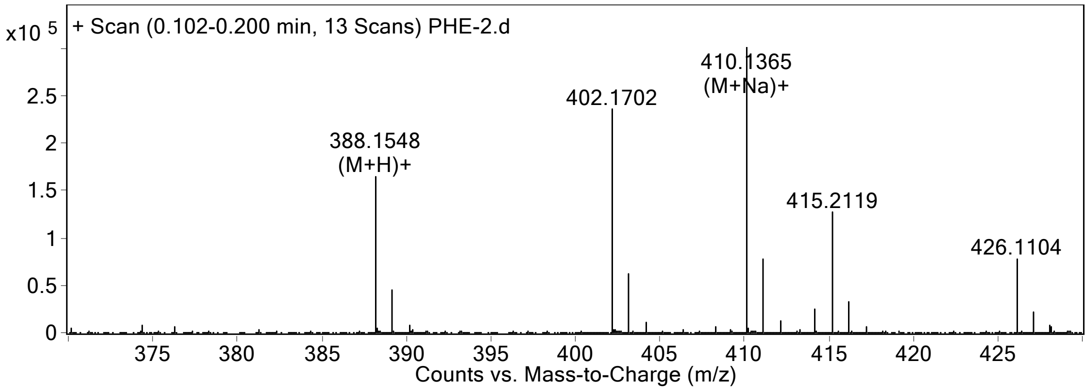

NOEs of compound 4g

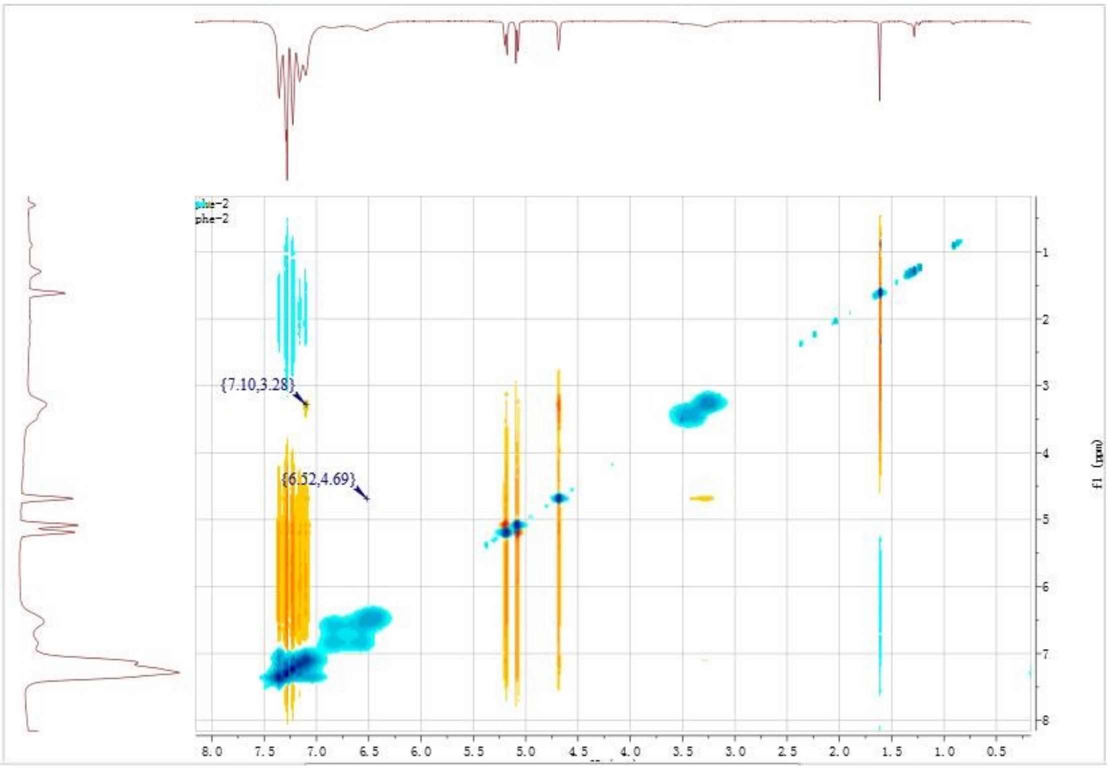

^{1}H-NMR of compound 5a ($CDCl_3$)

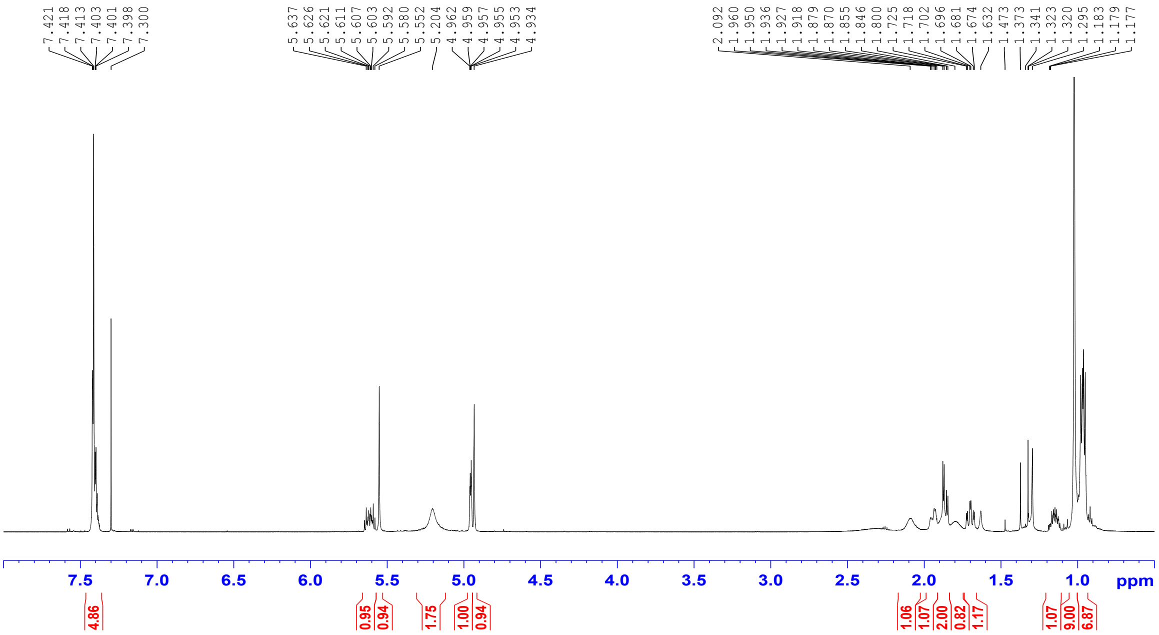

^{13}C-NMR of compound 5a ($CDCl_3$)

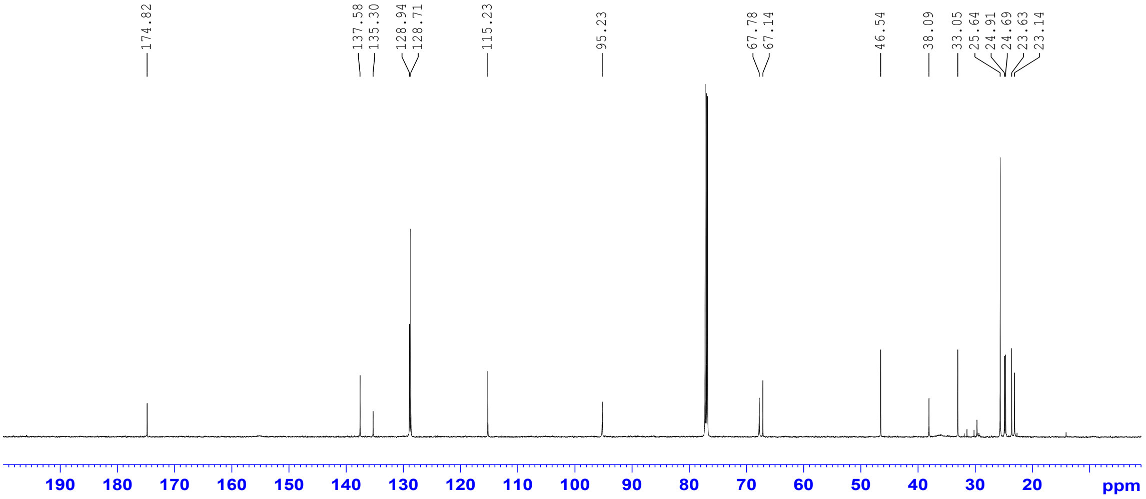

HR-Q-TOF-MS of compound 5a

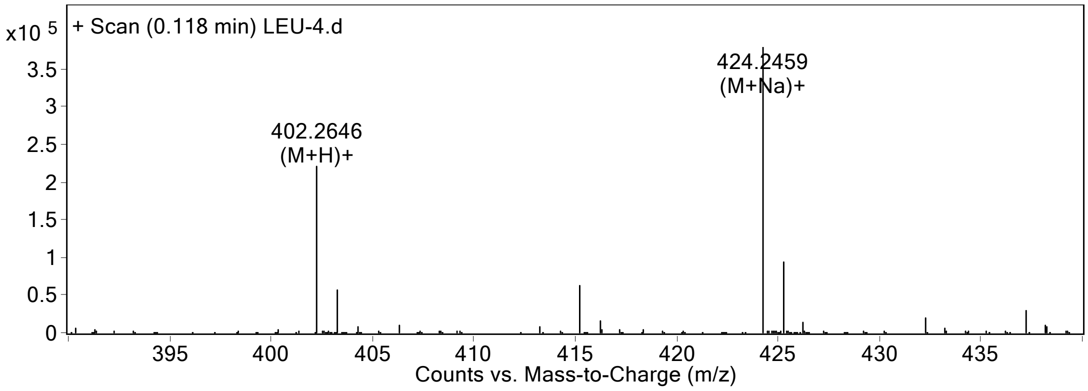

^{1}H-NMR of compound 5b ($CDCl_3$)

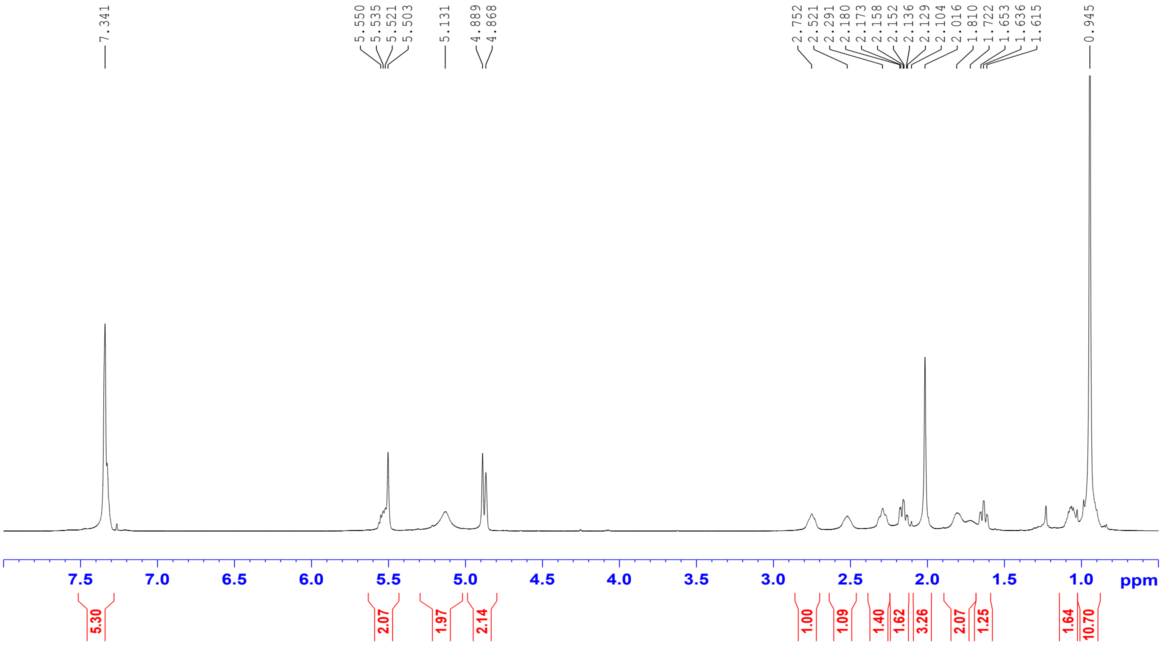

^{13}C-NMR of compound 5b ($CDCl_3$)

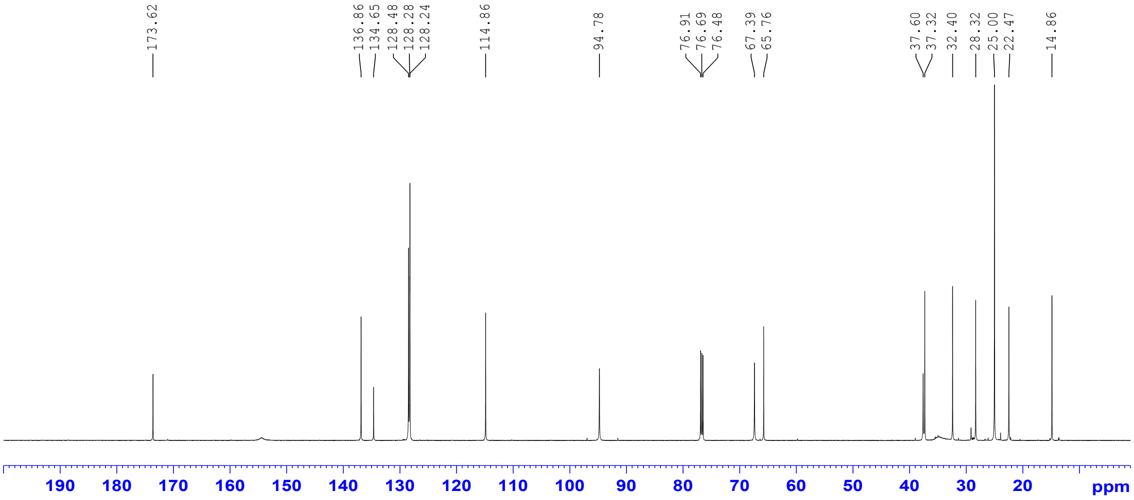

HR-Q-TOF-MS of compound 5b

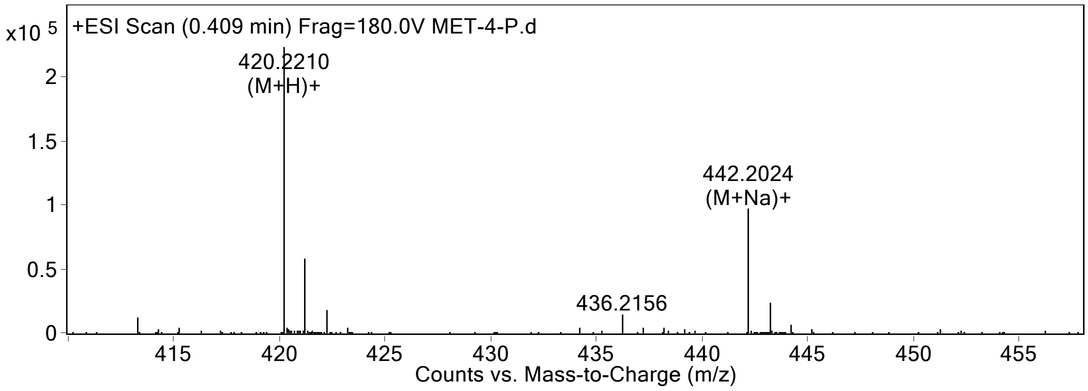

NOEs of compound 5b

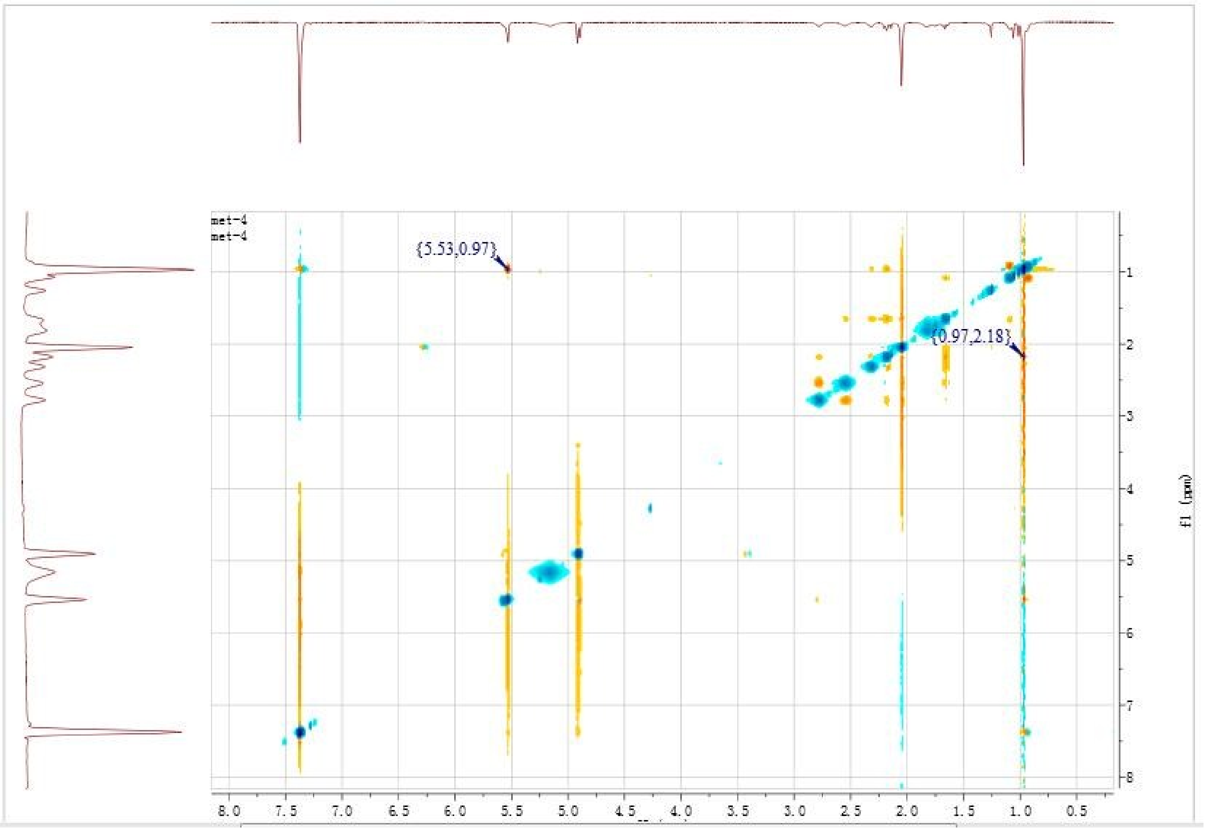

Partial NOEs of compound 4b and 5b

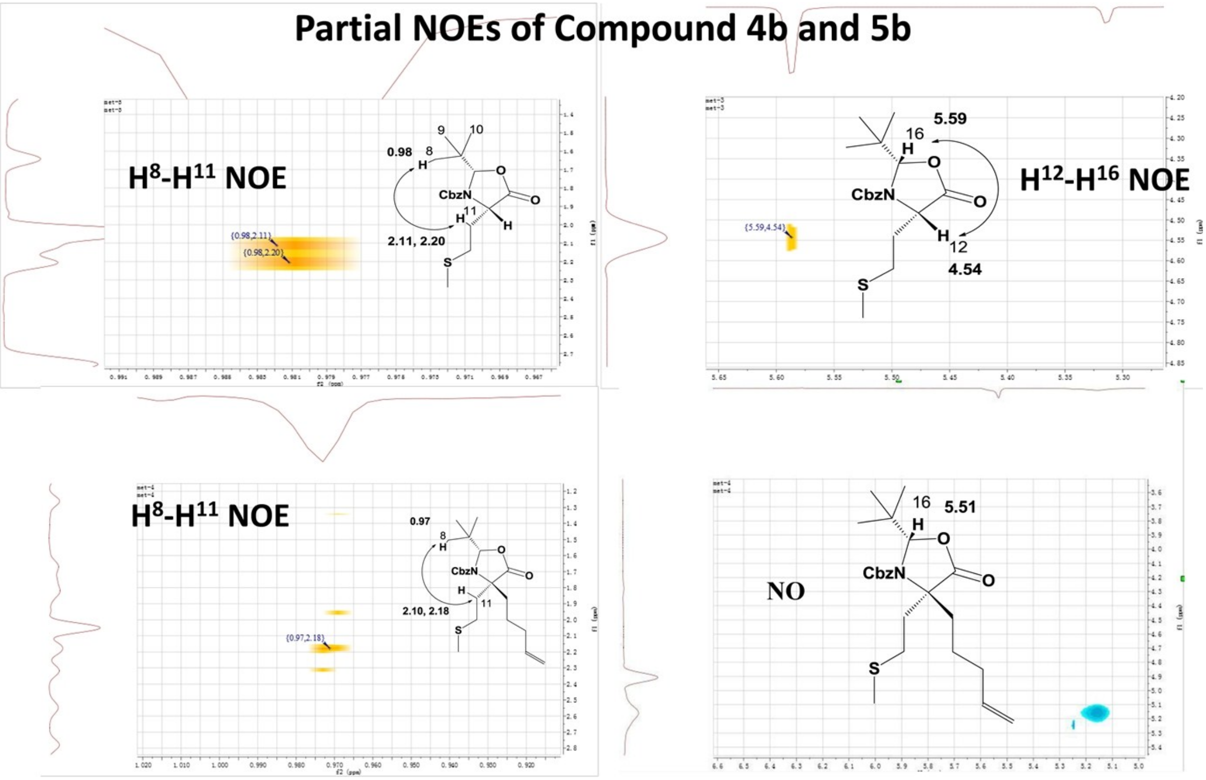

^{1}H-NMR of compound 5c ($CDCl_3$)

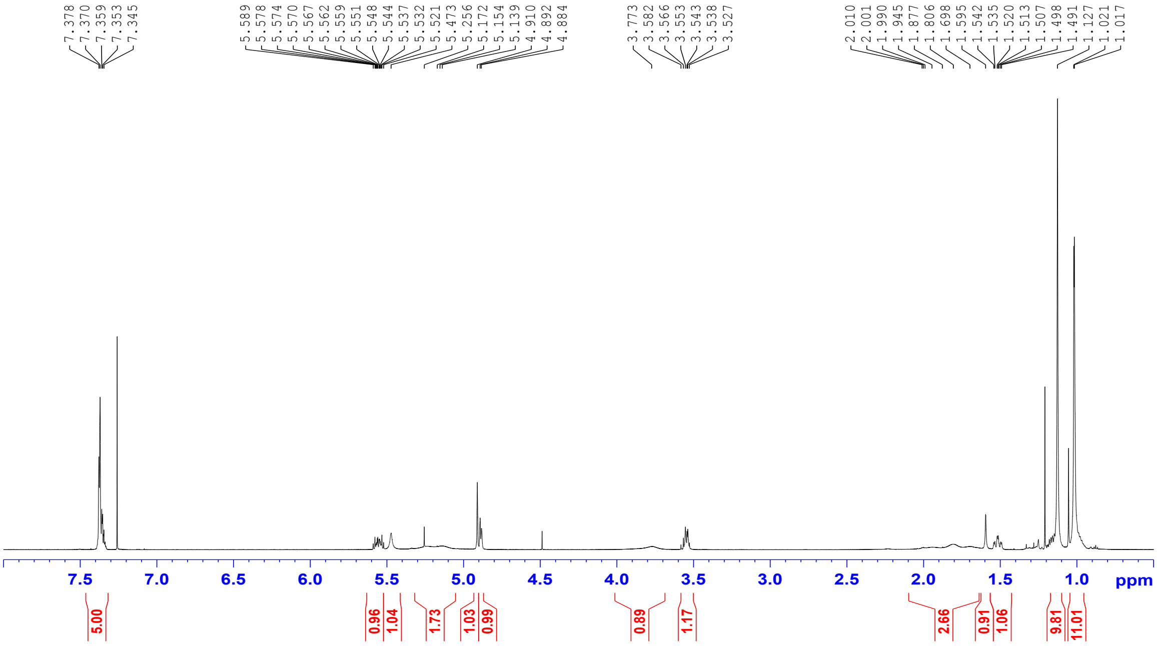

^{13}C-NMR of compound 5c ($CDCl_3$)

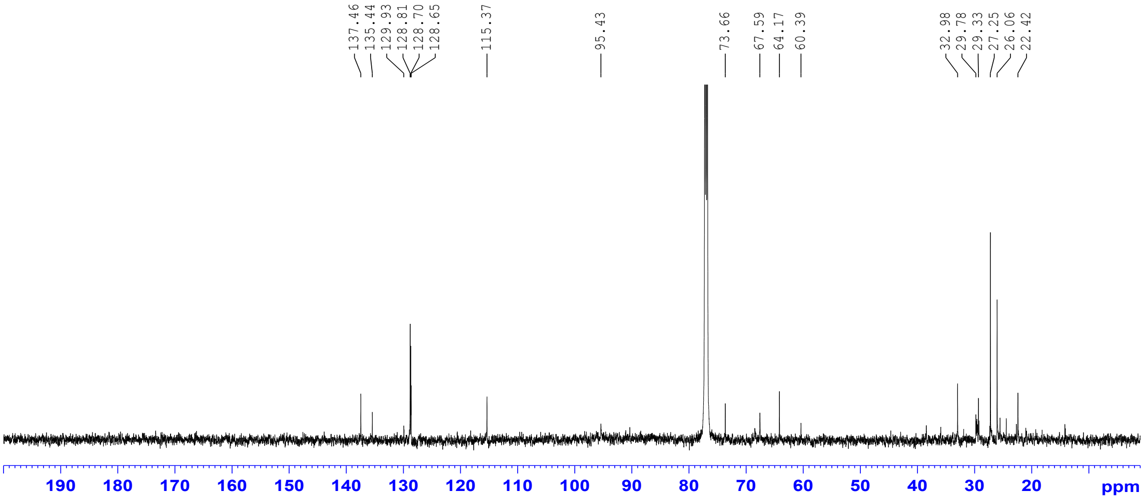

HR-Q-TOF-MS of compound 5c

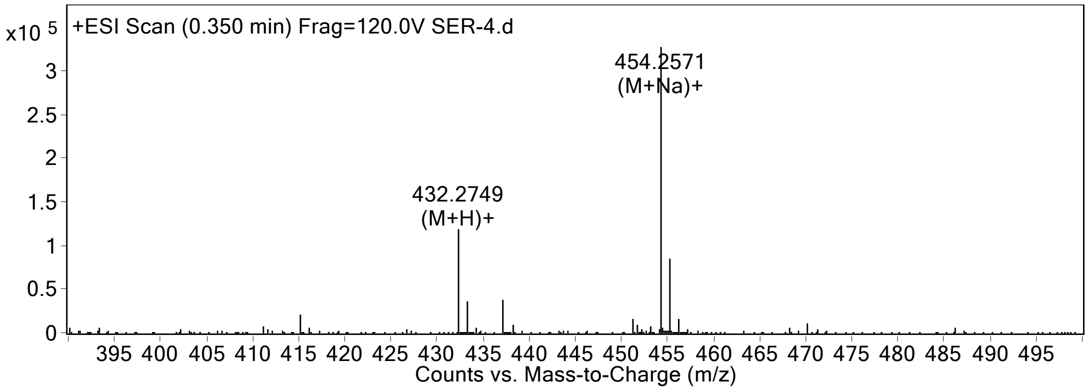

^{1}H-NMR of compound 5d ($CDCl_3$)

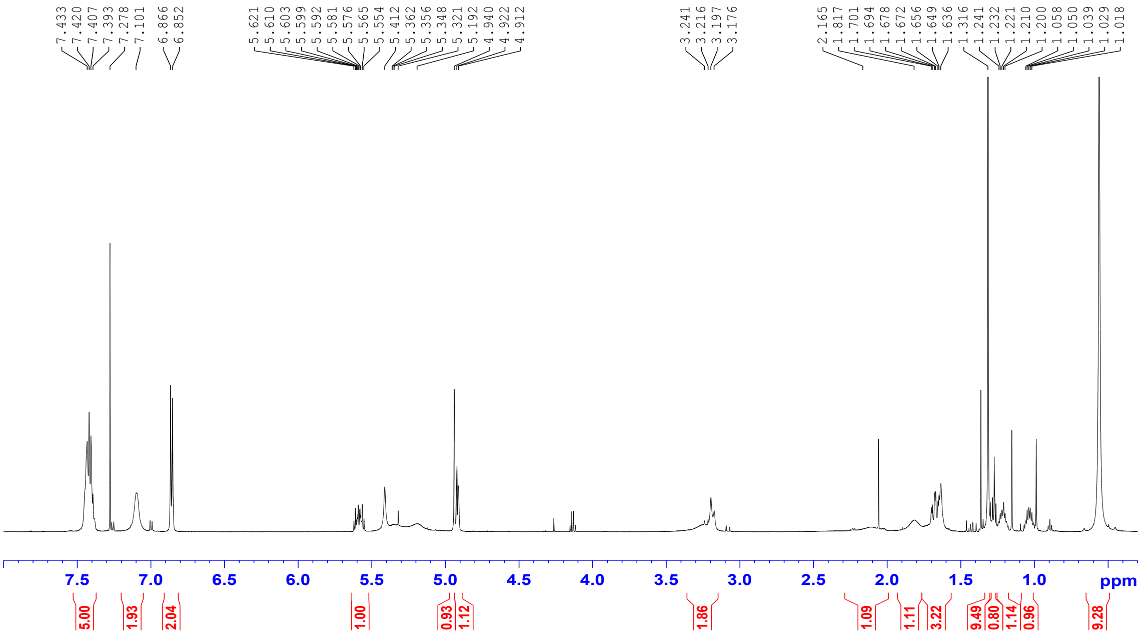

^{13}C-NMR of compound 5d ($CDCl_3$)

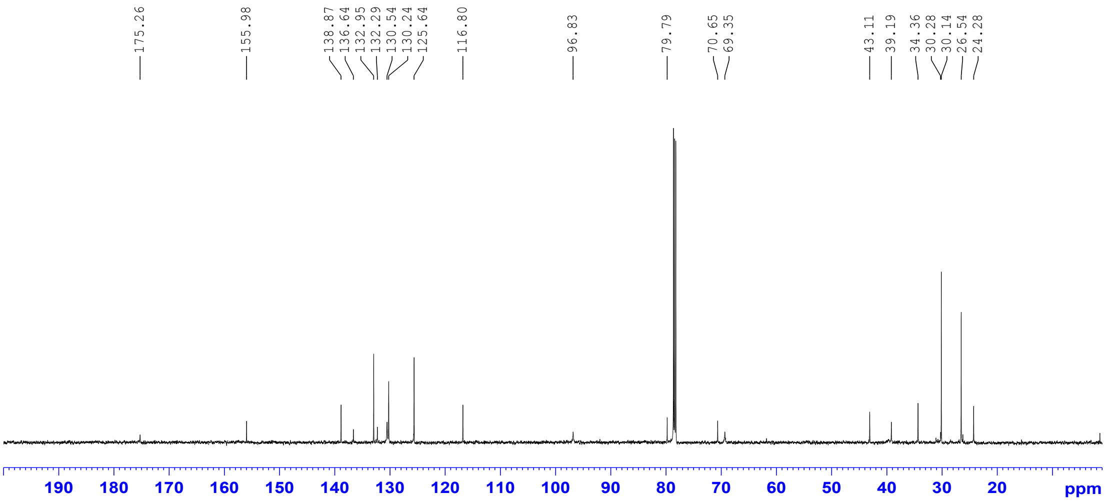

HR-Q-TOF-MS of compound 5d

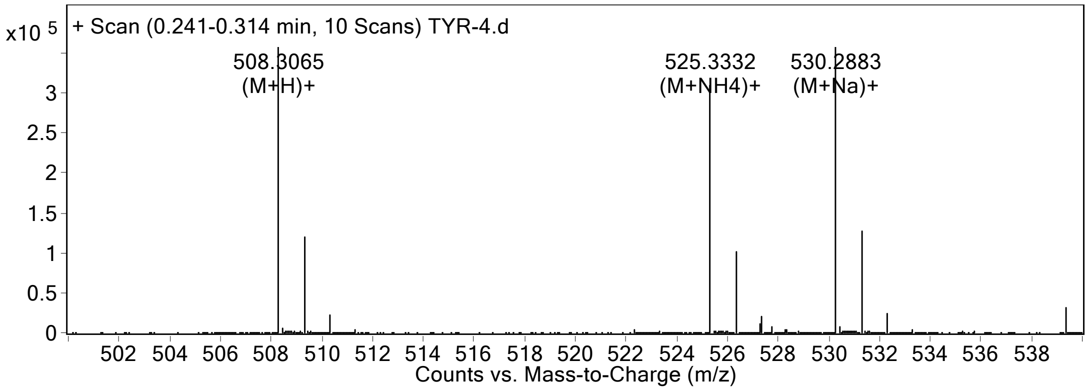

^{1}H-NMR of compound 5e ($CDCl_3$)

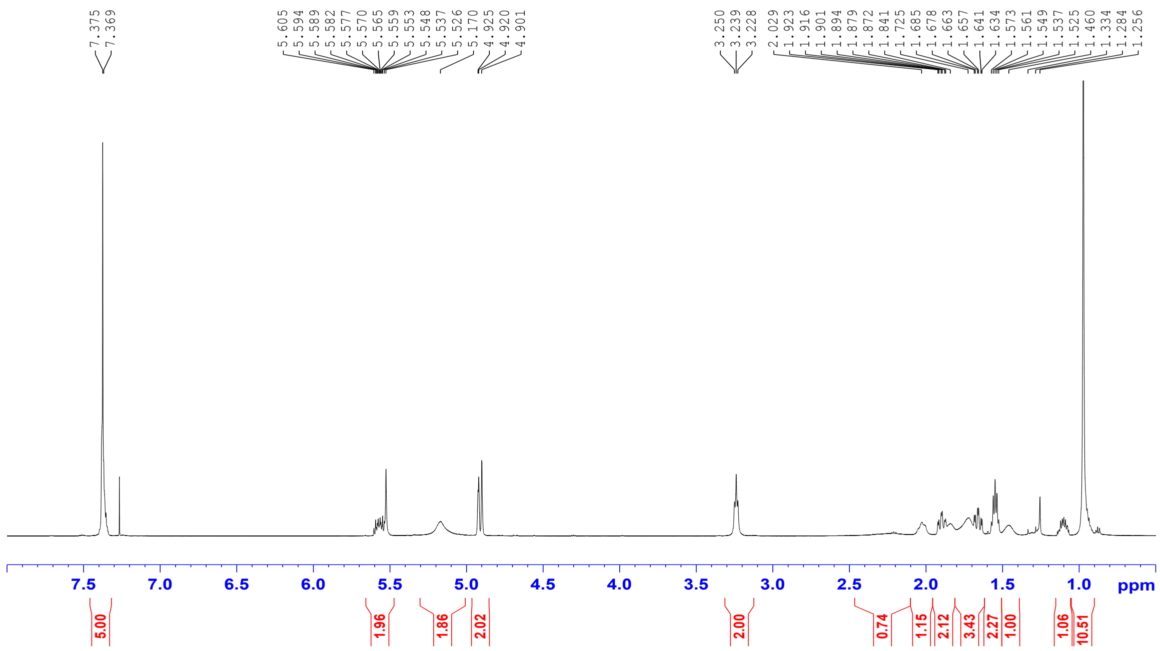

^{13}C-NMR of compound 5e ($CDCl_3$)

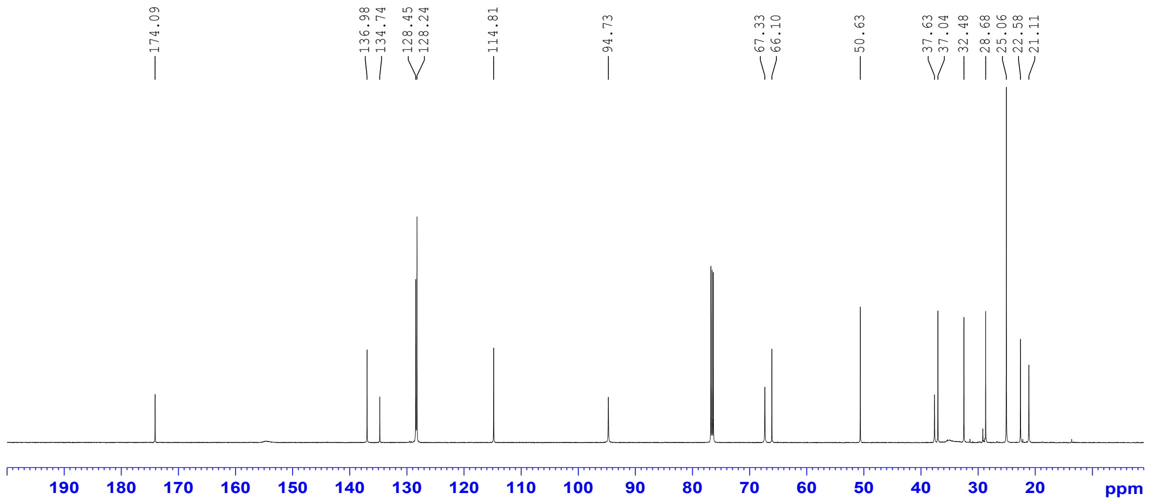

HR-Q-TOF-MS of compound 5e

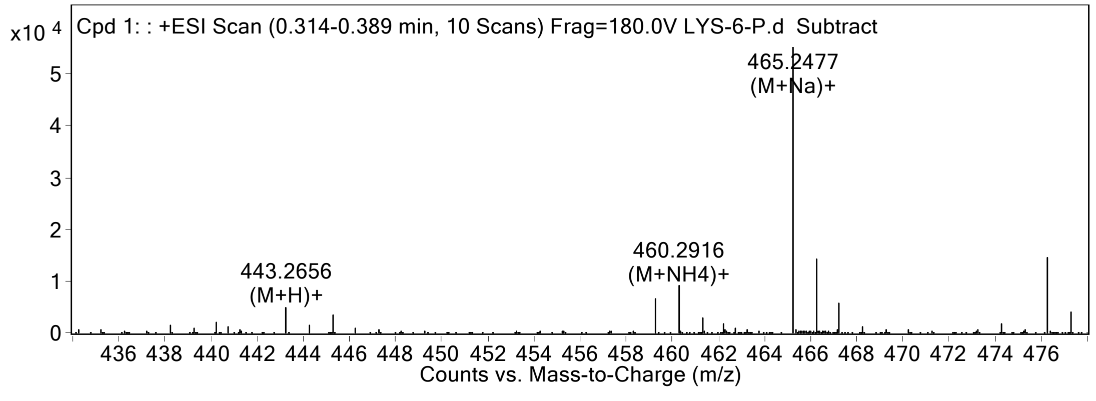

^{1}H-NMR of compound 5f ($CDCl_3$)

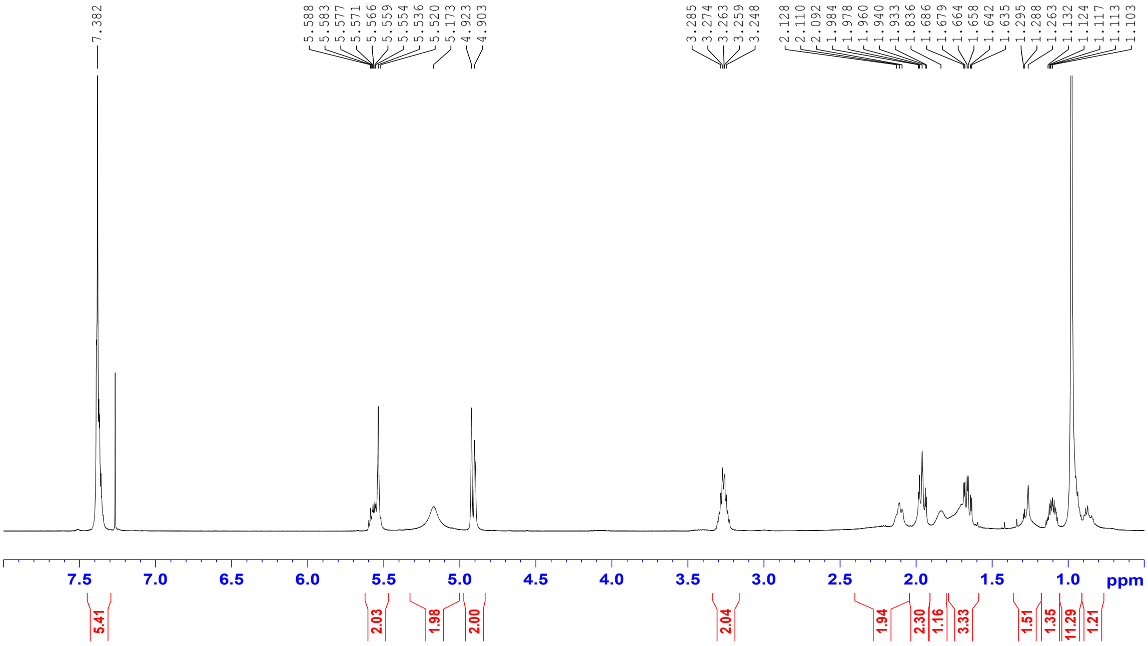

^{13}C-NMR of compound 5f ($CDCl_3$)

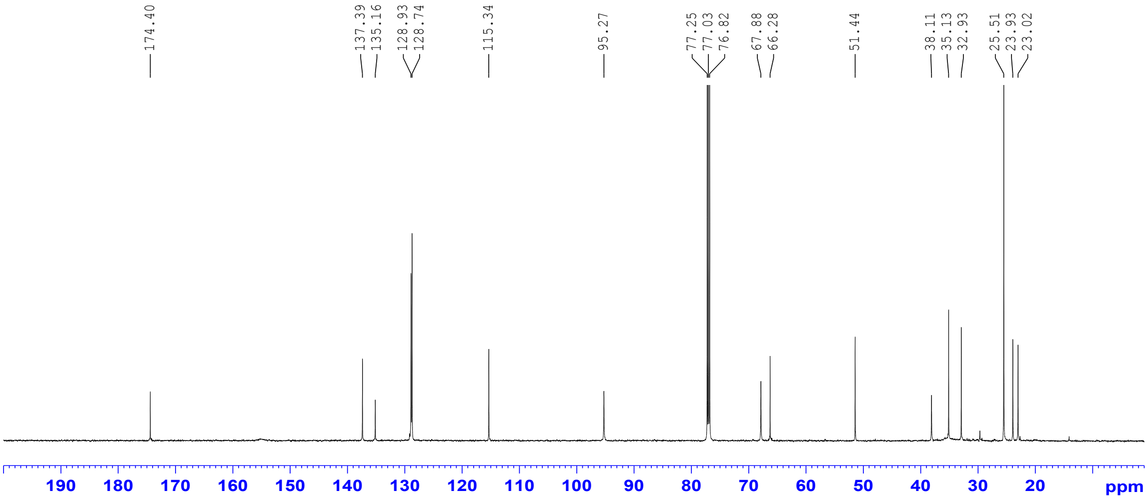

HR-Q-TOF-MS of compound 5f

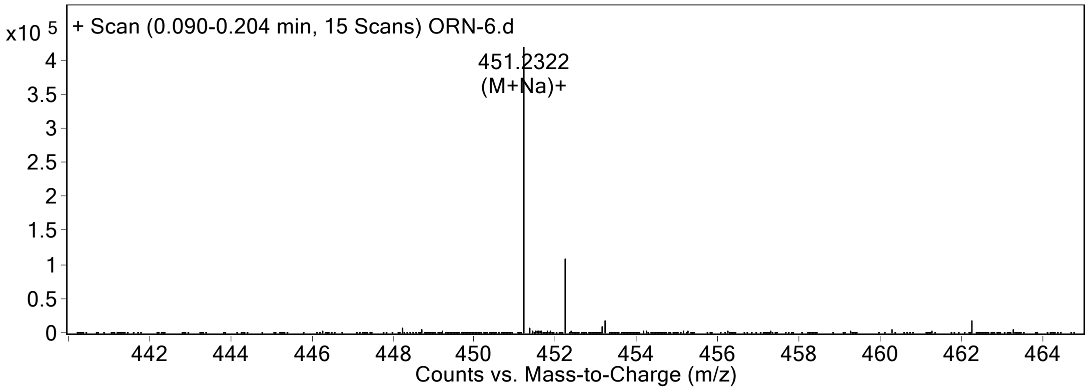

^{1}H-NMR of compound 5g ($CDCl_3$)

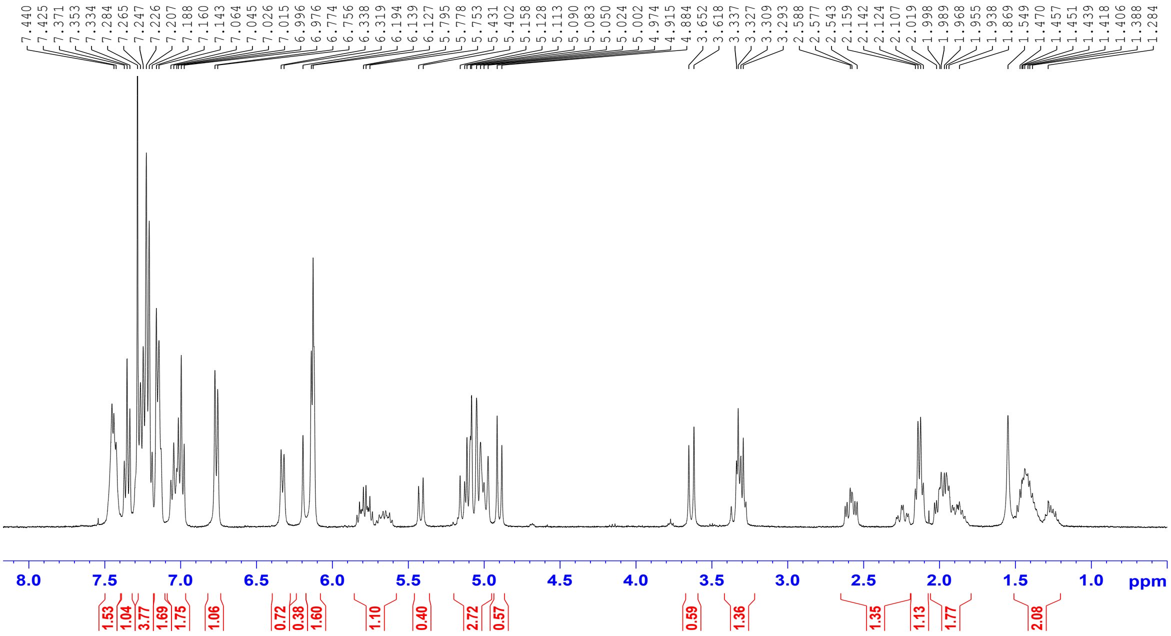

^{13}C-NMR of compound 5g ($CDCl_3$)

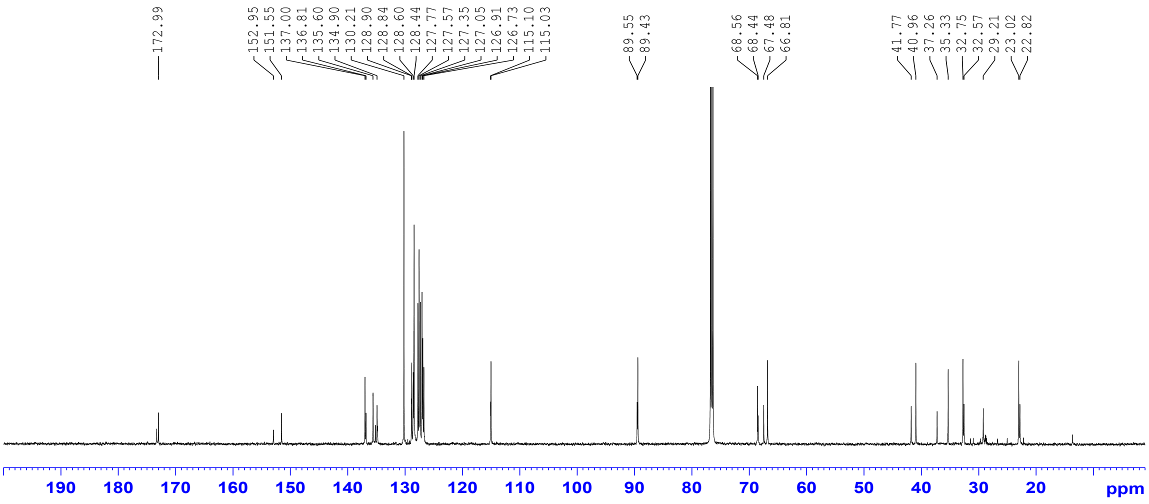

HR-Q-TOF-MS of compound 5g

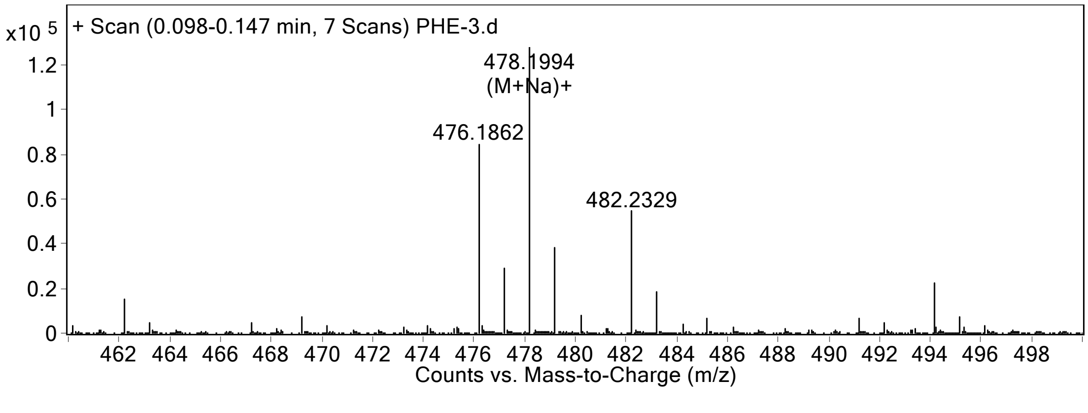

NOEs of compound 5g

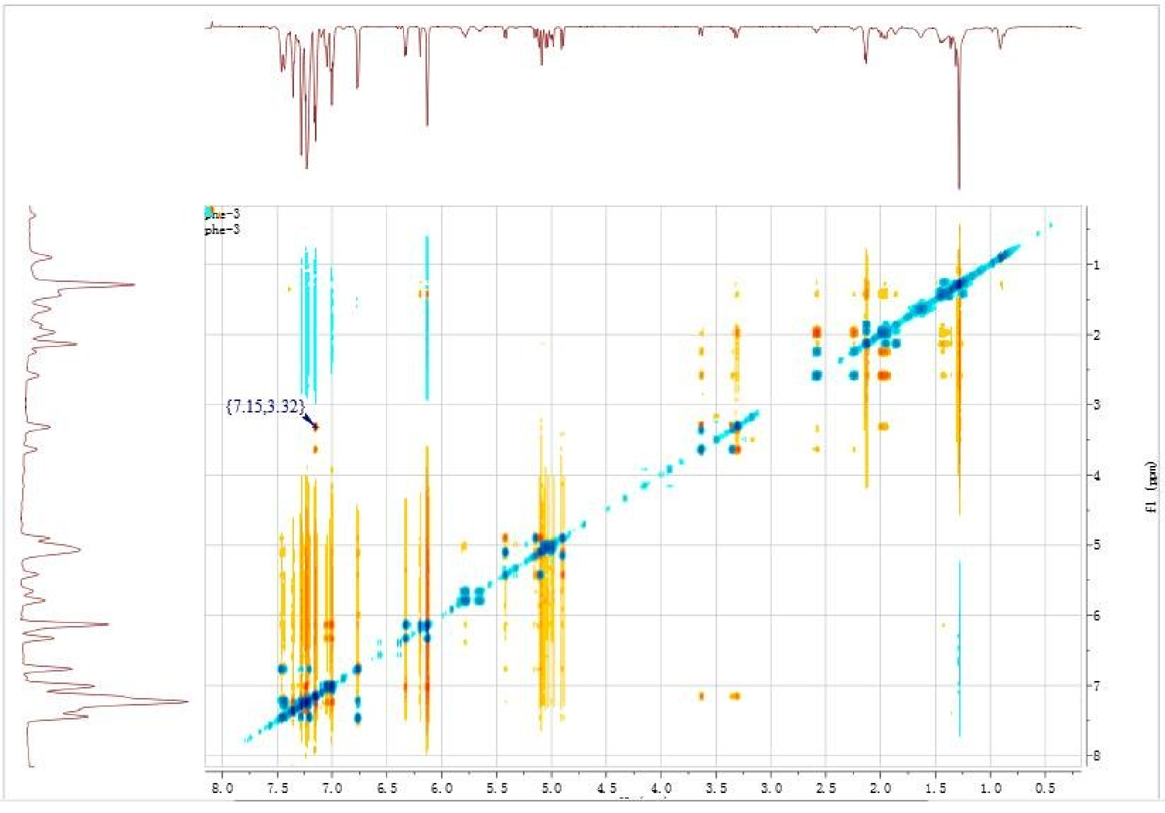

Partial NOEs of compound 4g and 5g

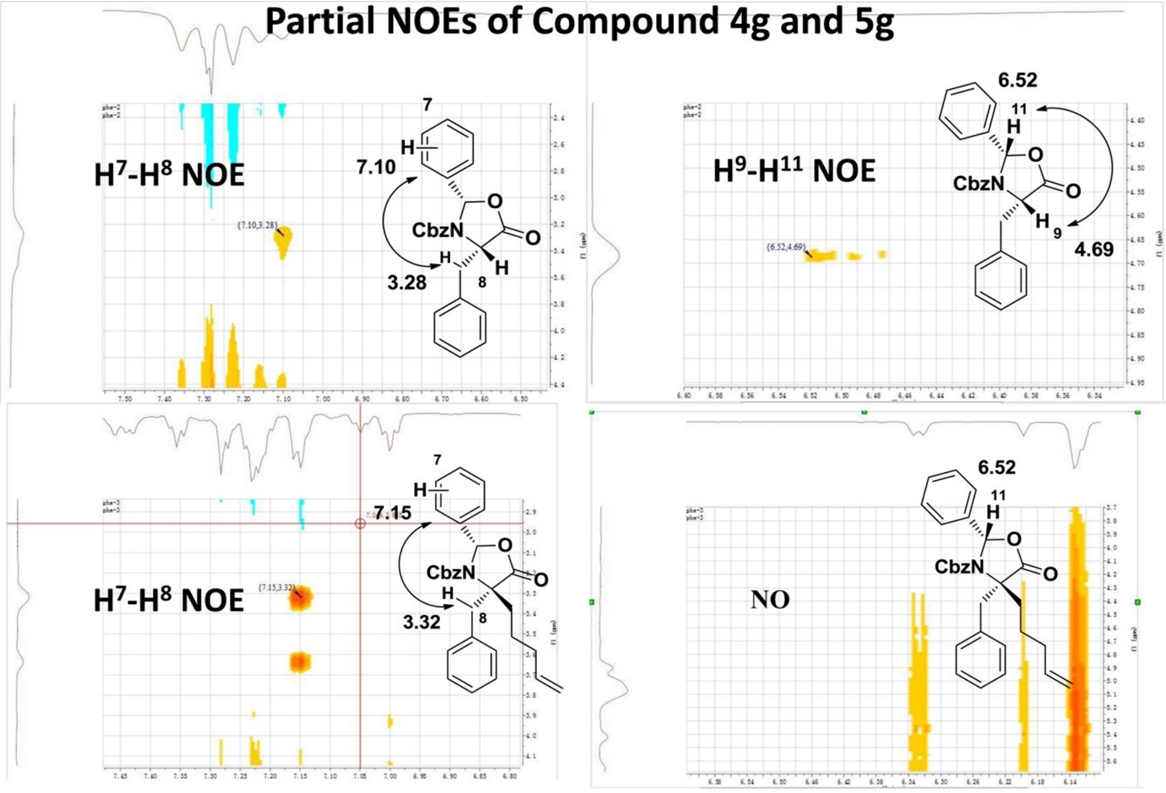

^{1}H-NMR of compound 1a (MeOD)

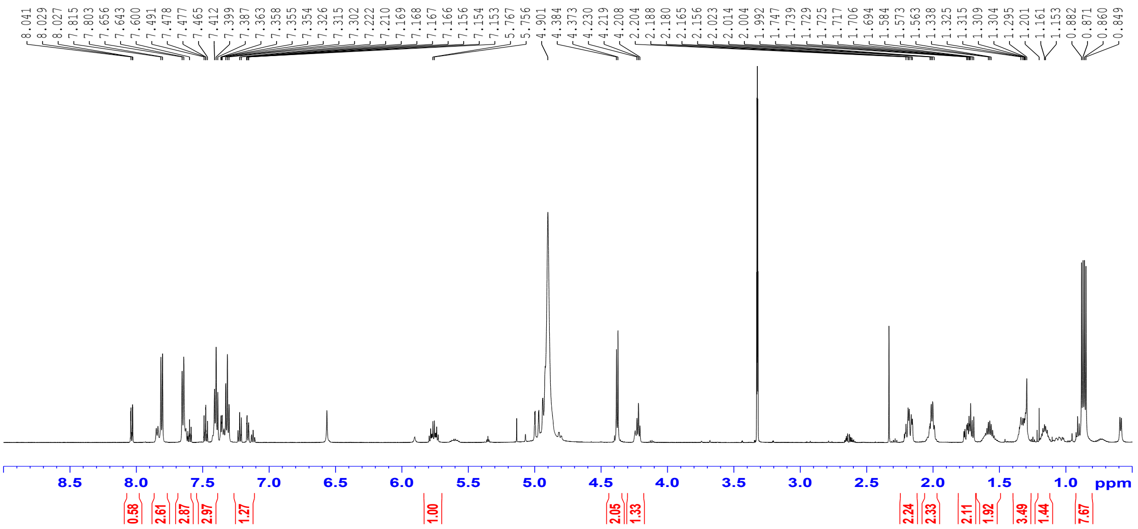

^{13}C-NMR of compound 1a (MeOD)

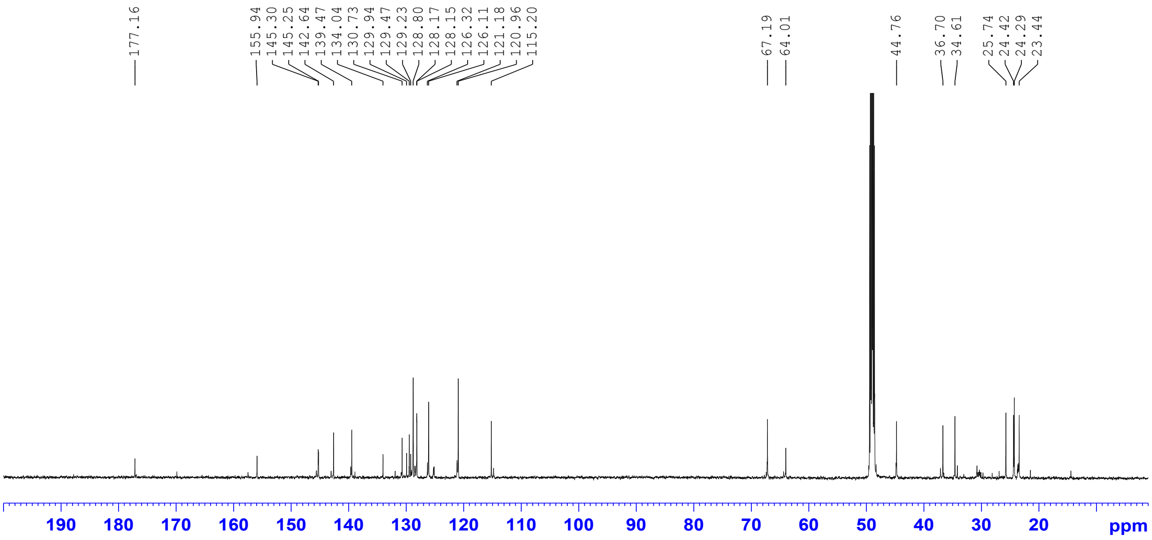

HR-Q-TOF-MS of compound 1a

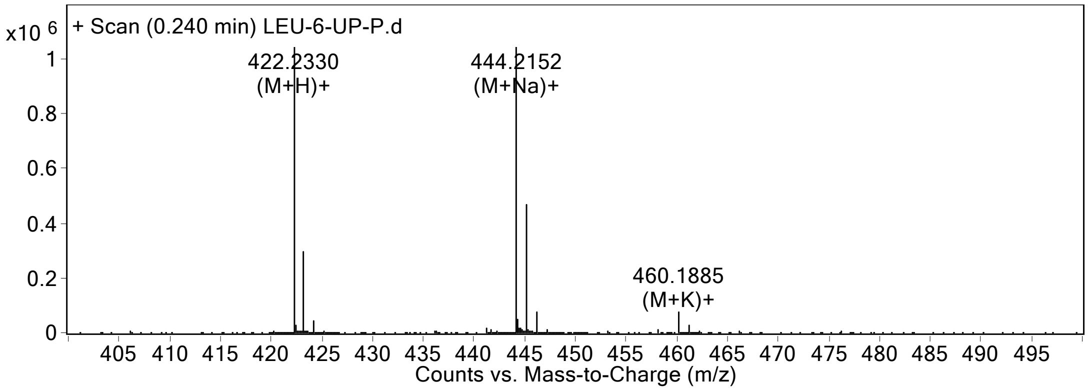

^{1}H-NMR of compound 1b (MeOD)

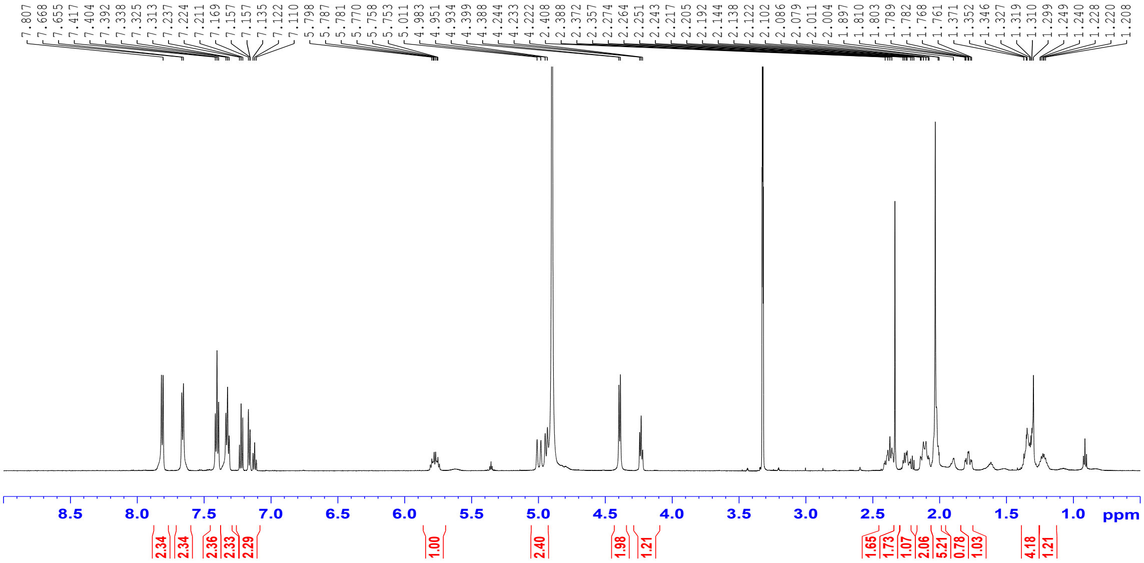

^{13}C-NMR of compound 1b (MeOD)

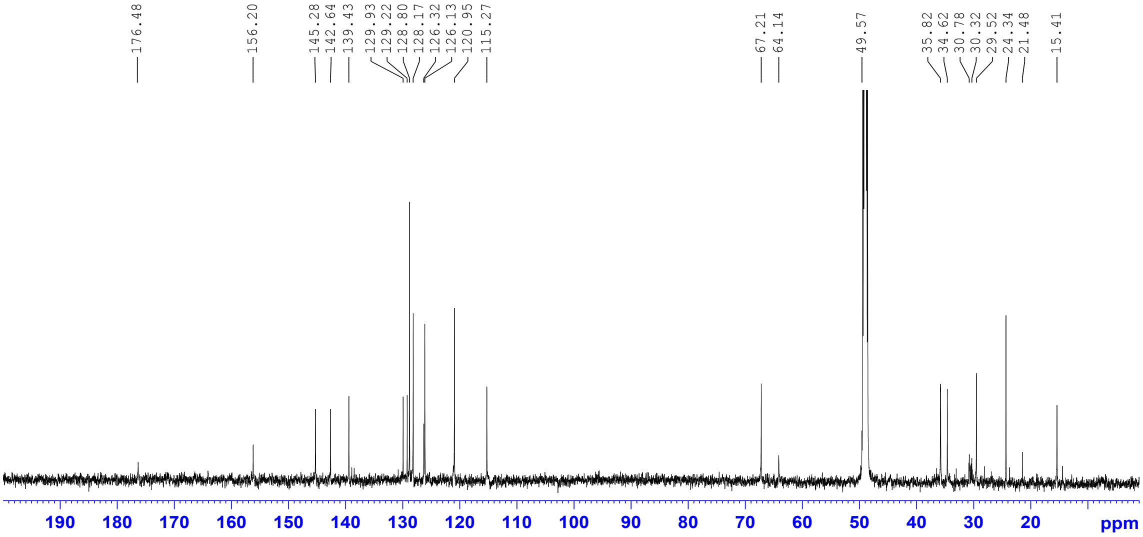

HR-Q-TOF-MS of compound 1b

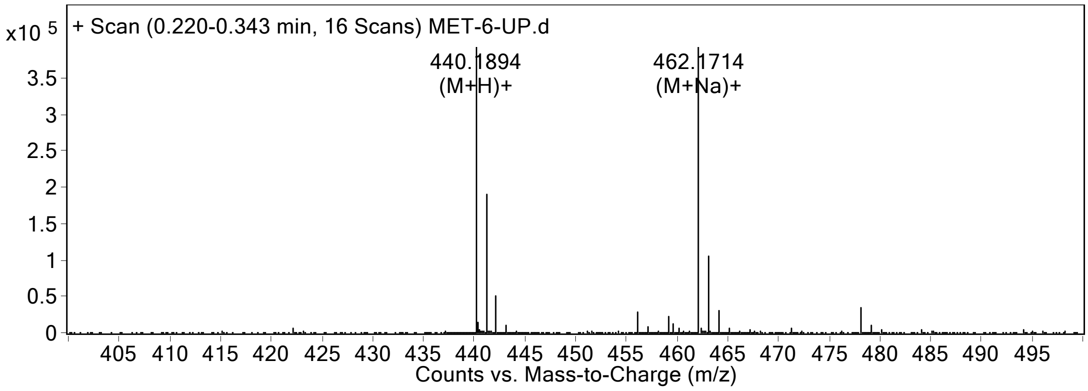

^{1}H-NMR of compound 1c (MeOD)

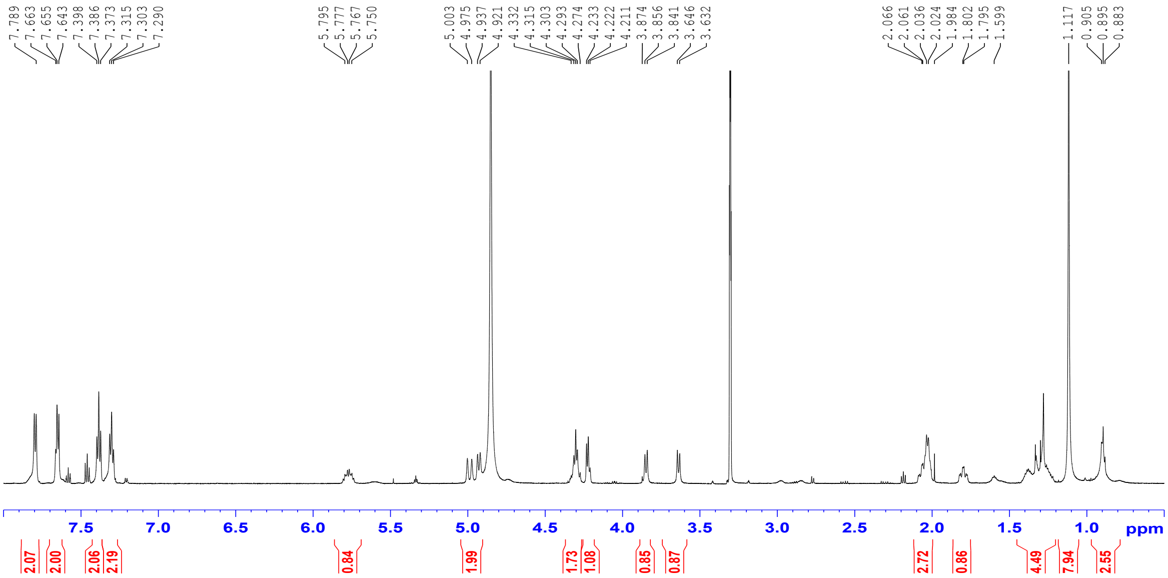

^{13}C-NMR of compound 1c (MeOD)

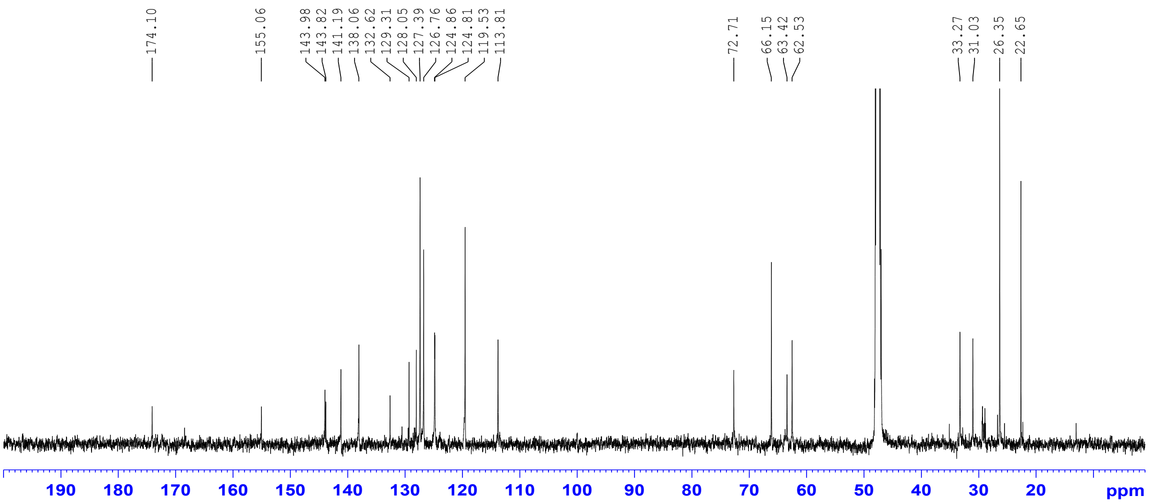

HR-Q-TOF-MS of compound 1c

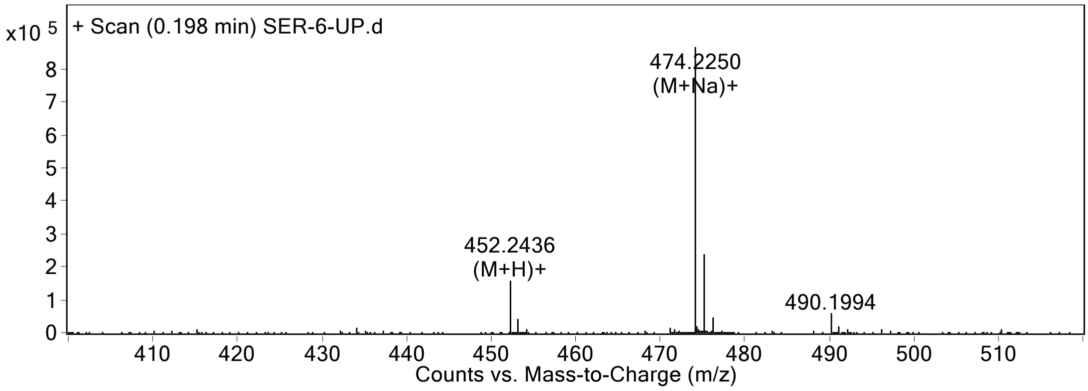

^{1}H-NMR of compound 1d ($CDCl_3$)

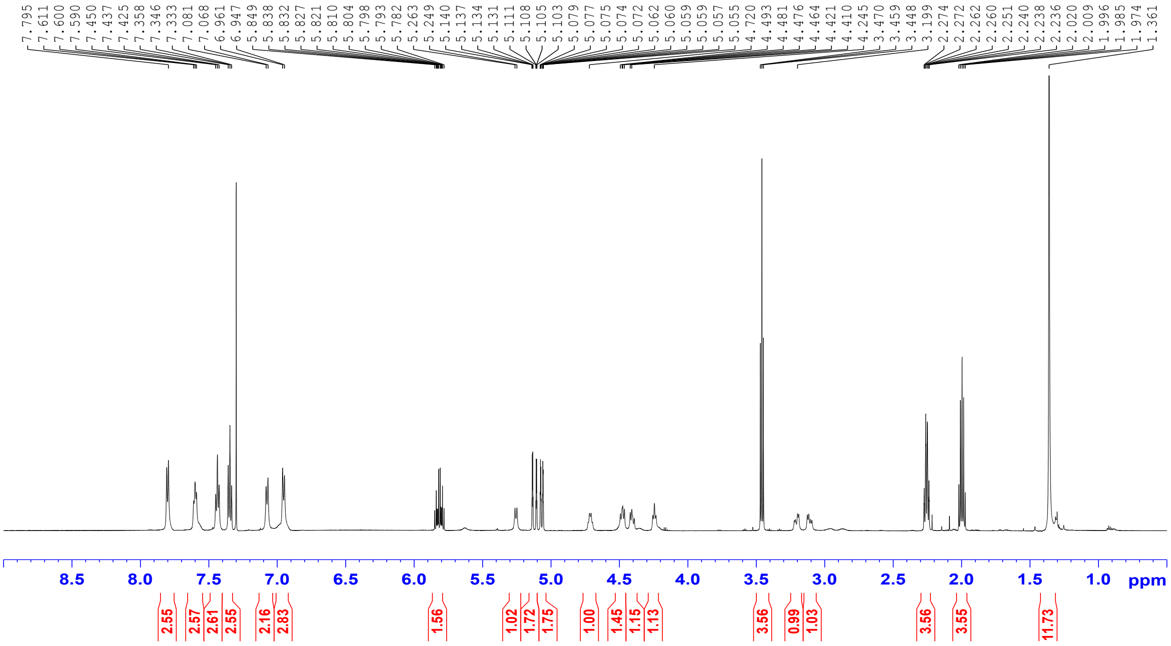

^{13}C-NMR of compound 1d ($CDCl_3$)

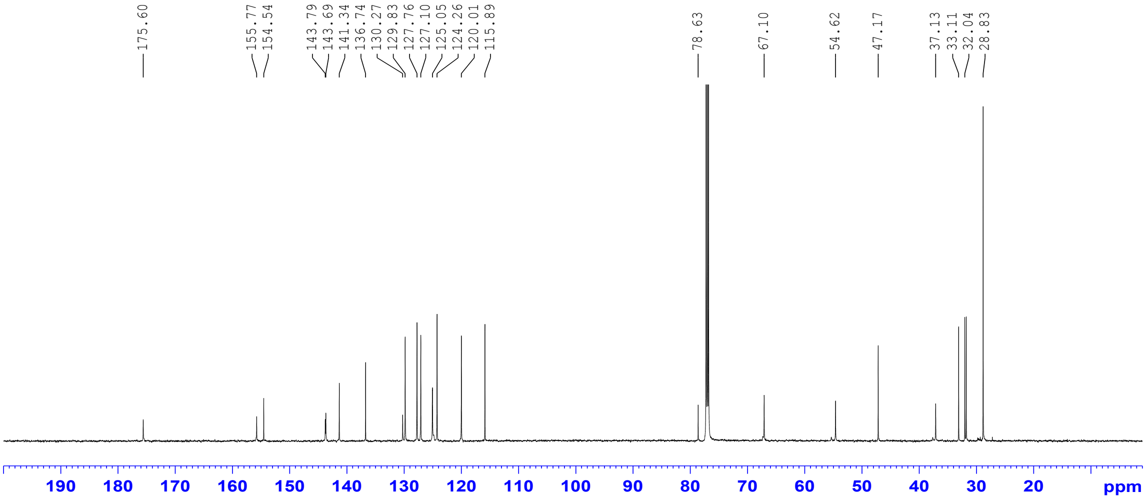

HR-Q-TOF-MS of compound 1d

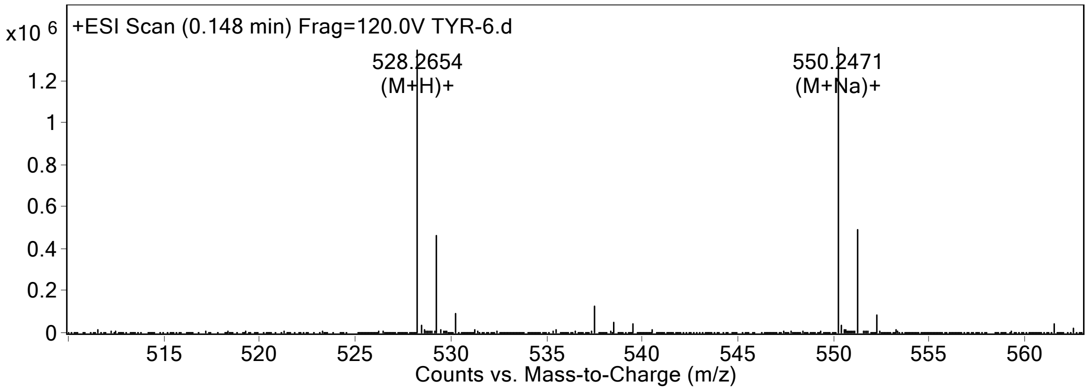

^{1}H-NMR of compound 1e (MeOD)

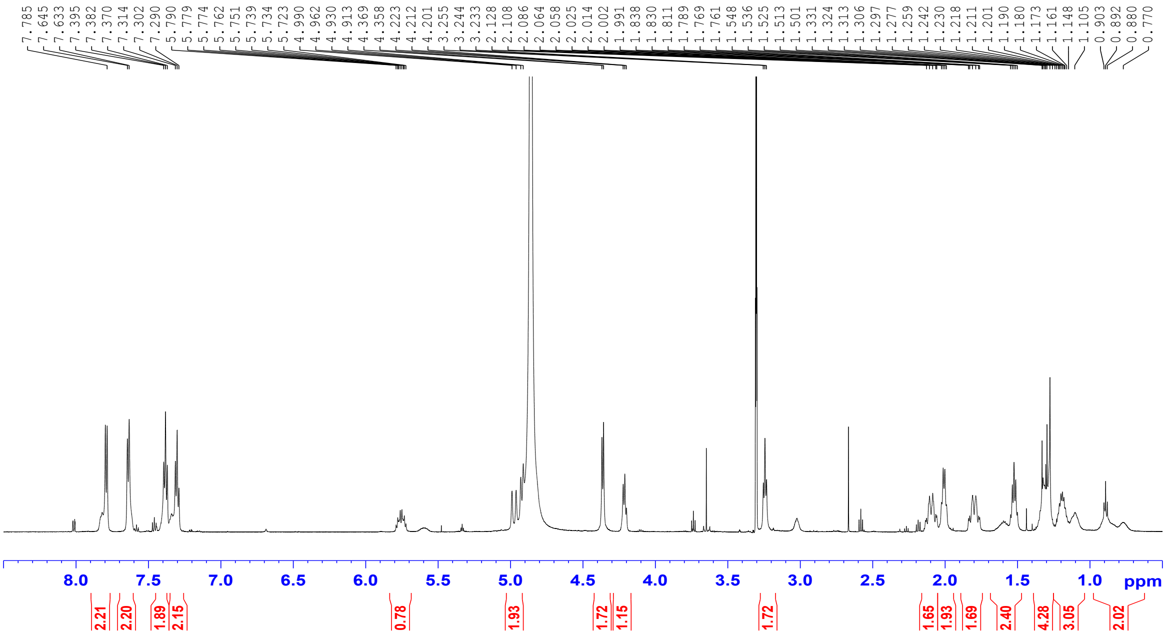

^{13}C-NMR of compound 1e (MeOD)

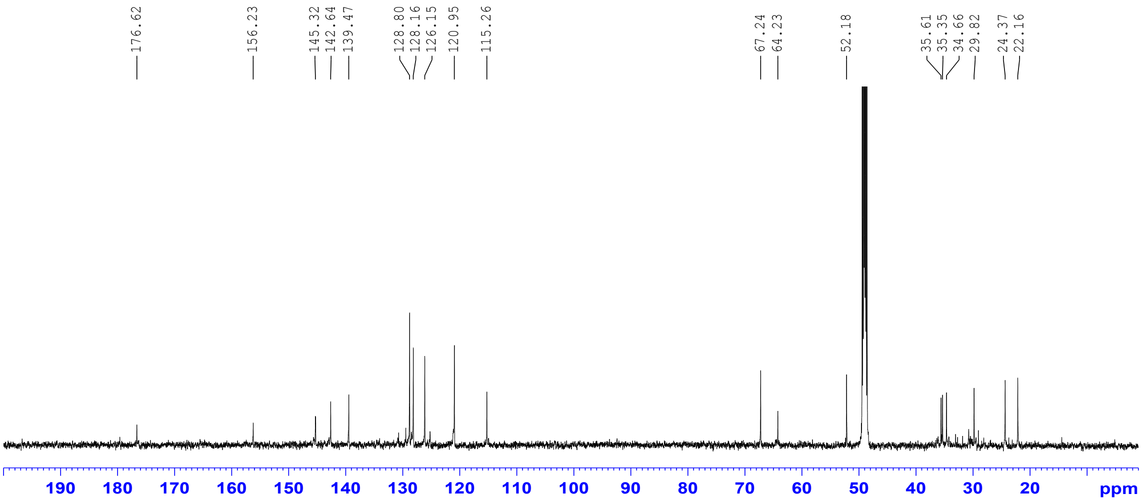

HR-Q-TOF-MS of compound 1e

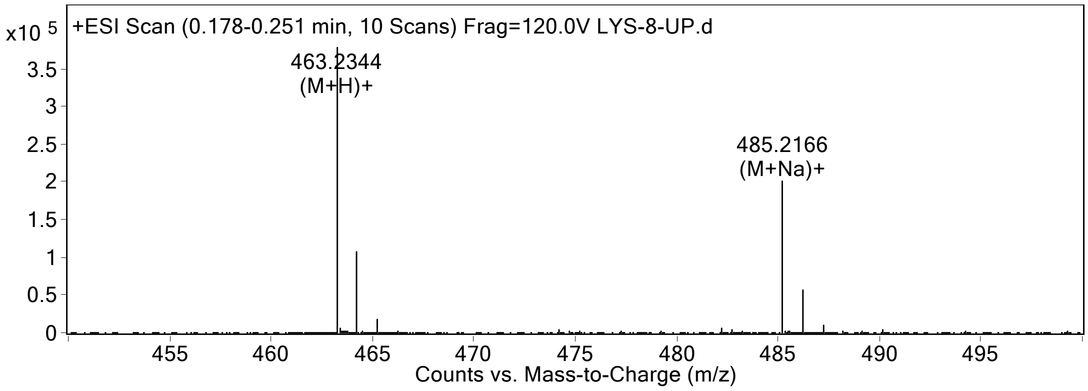

^{1}H-NMR of compound 7f (MeOD)

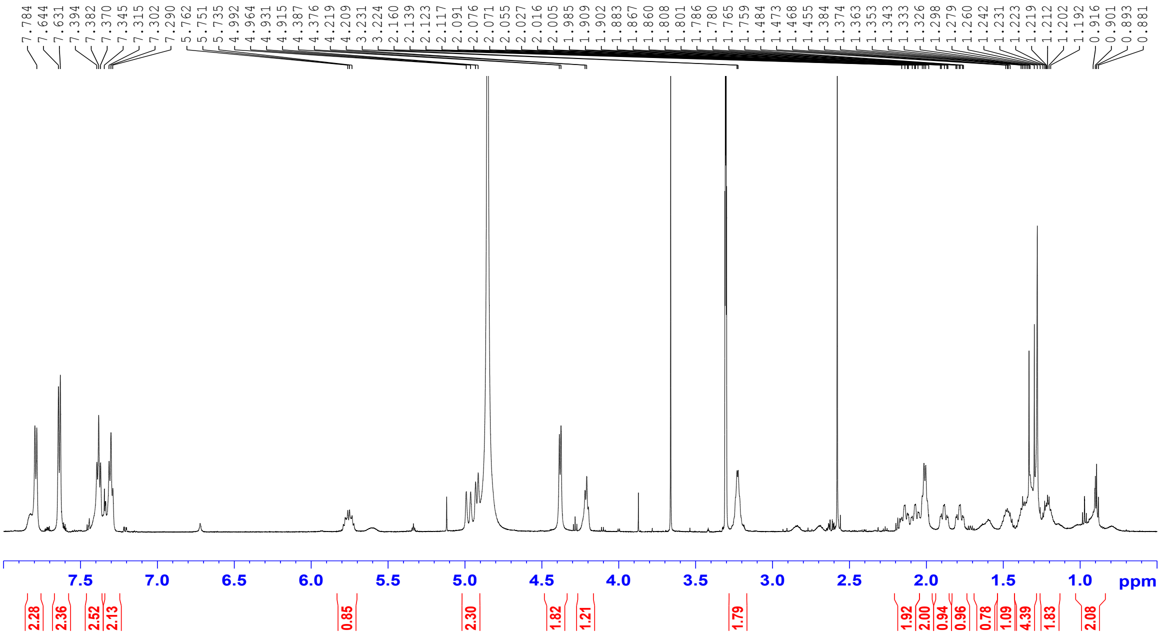

^{13}C-NMR of compound 7f (MeOD)

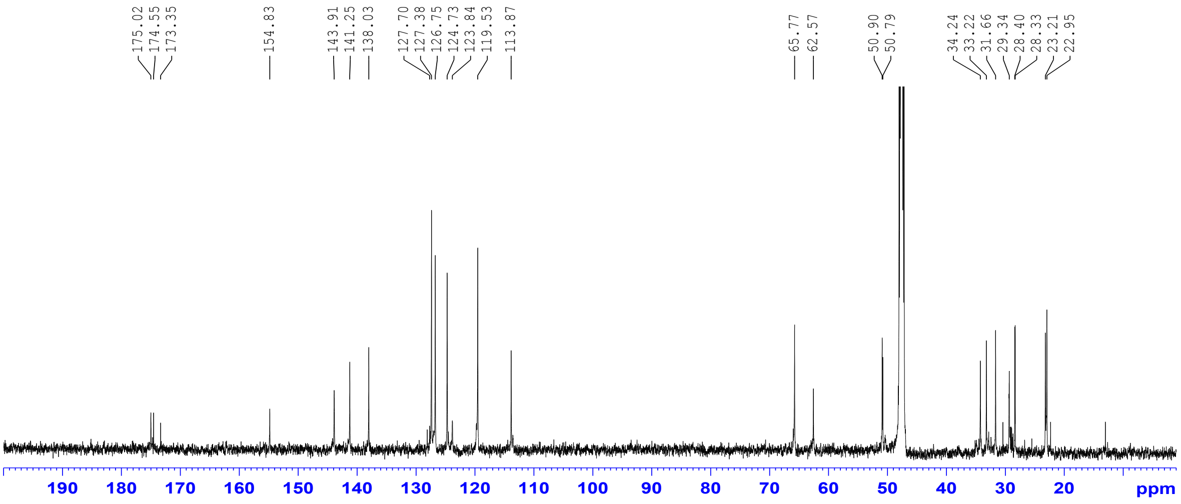

HR-Q-TOF-MS of compound 7f

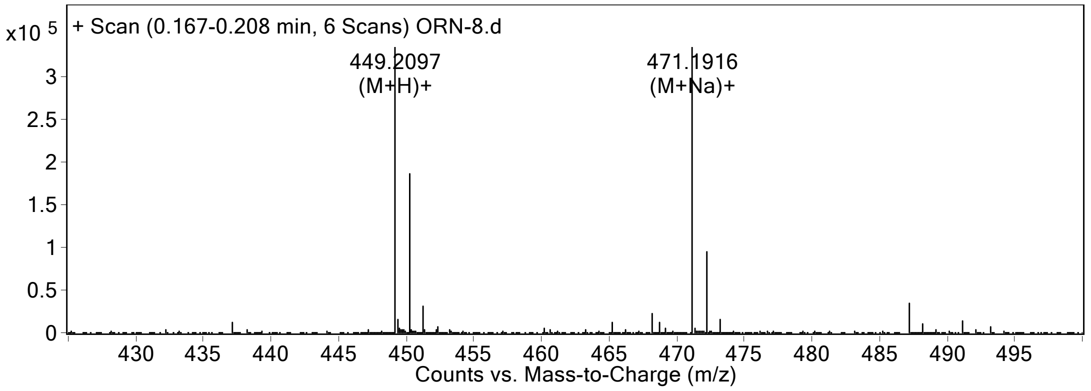

^{1}H-NMR of compound 1g ($CDCl_3$)

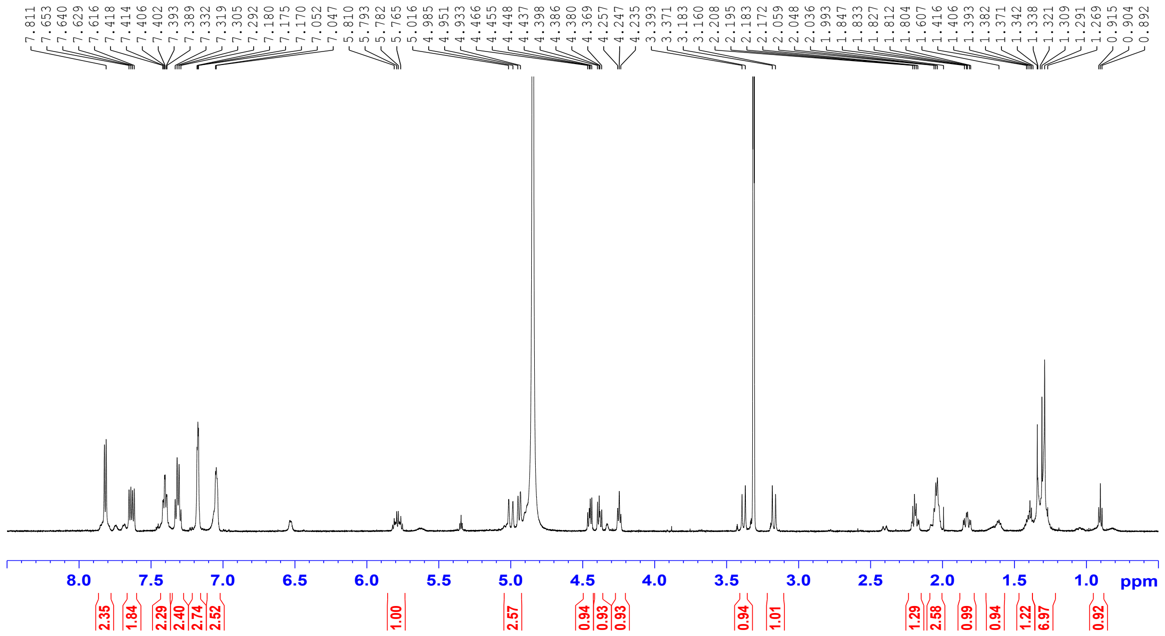

^{13}C-NMR of compound 1g ($CDCl_3$)

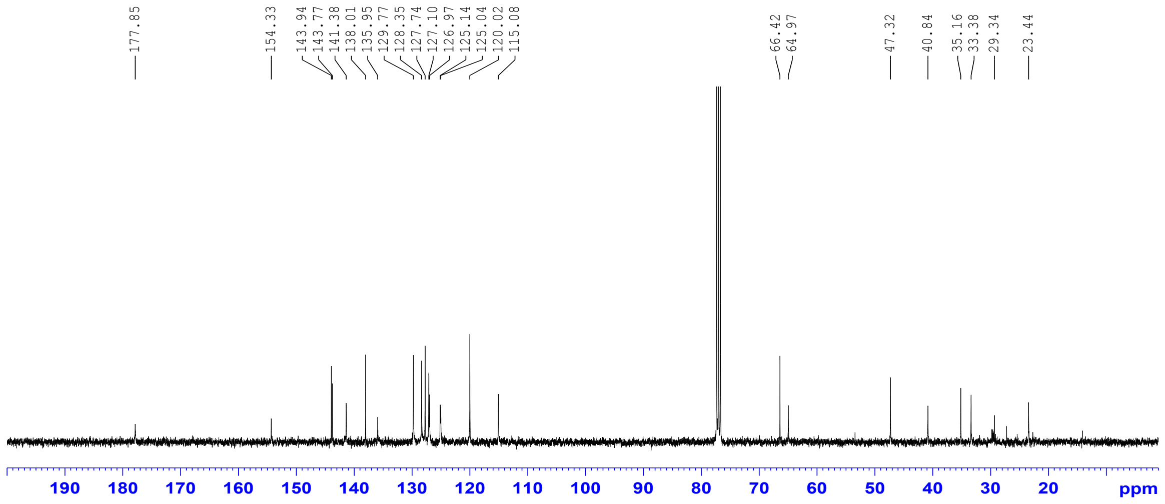

HR-Q-TOF-MS of compound 1g

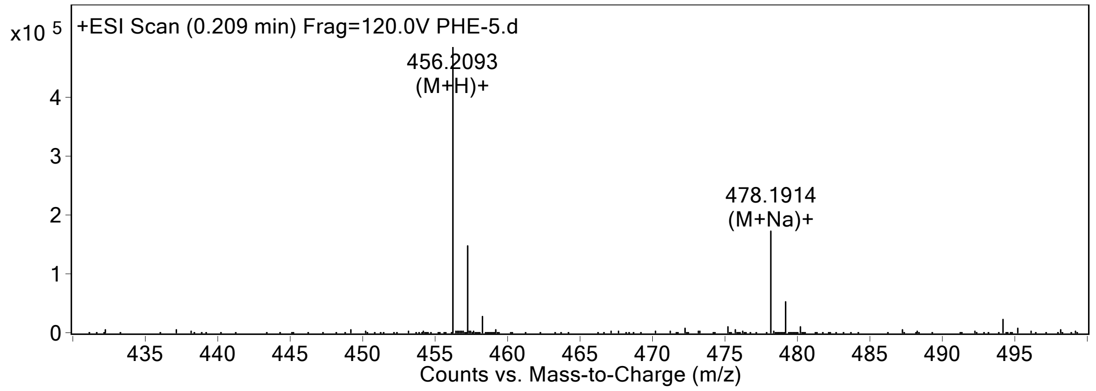

Chiral HPLC of compound 1g

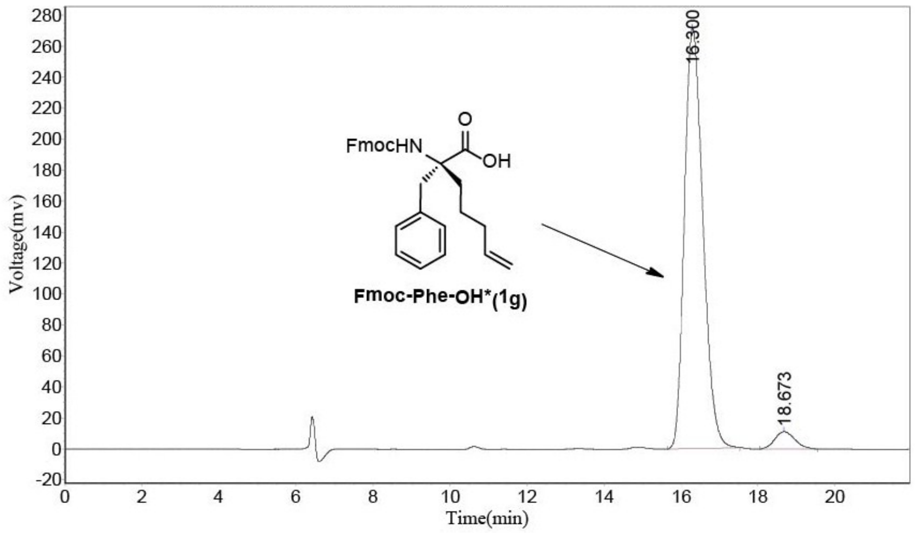

Results

Peak No.	Peak ID	Ret Time	Height	Area	Conc.
1		16.300	271376.906	9081347.000	95.8248
2		18.673	11084.659	395686.688	4.1752
Total			282461.565	9477033.688	100.0000

Chiral HPLC of the mixture of compound 1g and 1g`

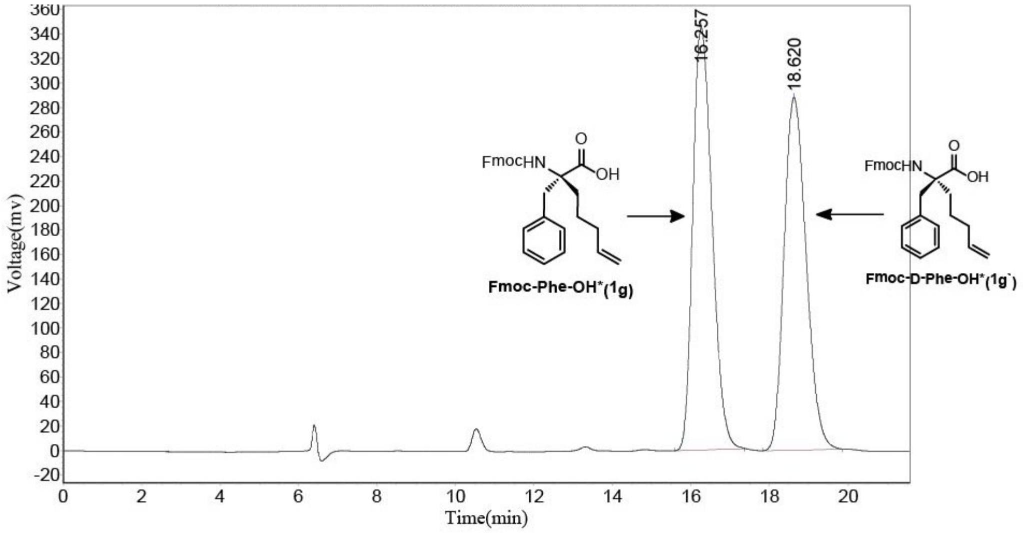

Results

Peak No.	Peak ID	Ret Time	Height	Area	Conc.
1		16.257	344795.563	11772904.000	51.1794
2		18.620	287733.844	11230326.000	48.8206
Total			632529.406	23003230.000	100.0000

^{1}H-NMR of compound 1f ($CDCl_3$)

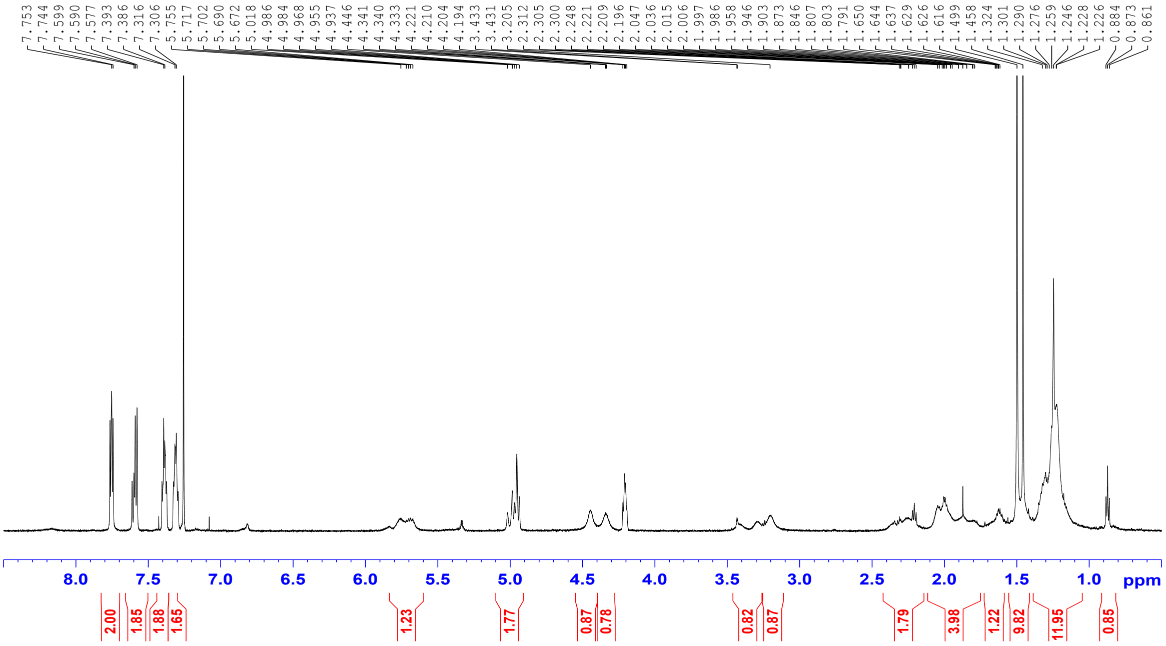

^{13}C-NMR of compound 1f ($CDCl_3$)

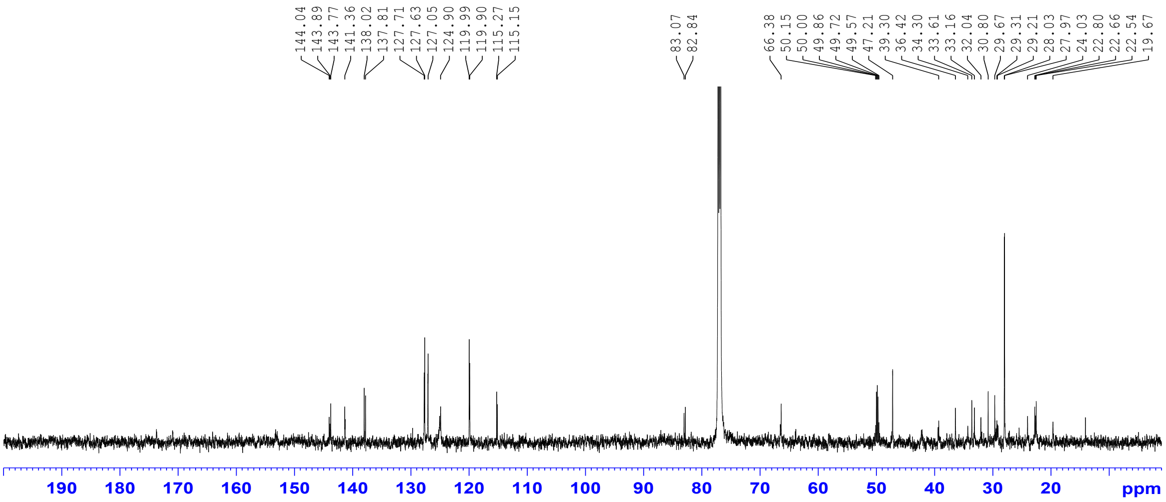

HR-Q-TOF-MS of compound 1f

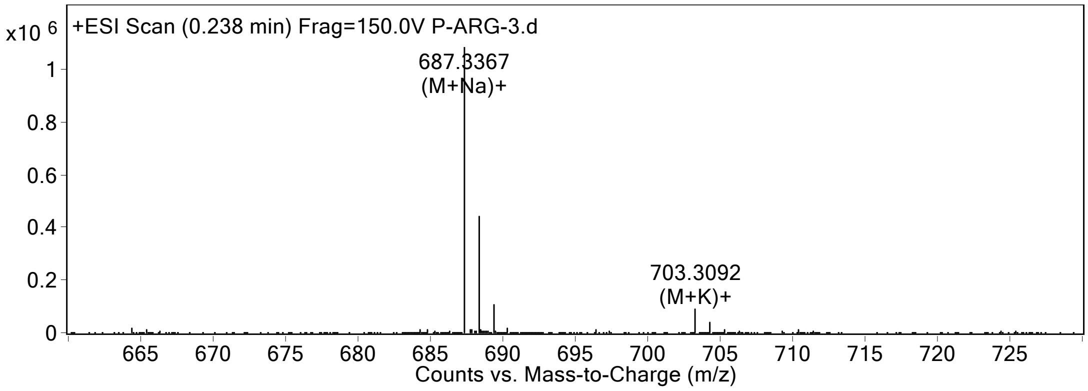

HR-Q-TOF-MS of compound Axin (469-482)

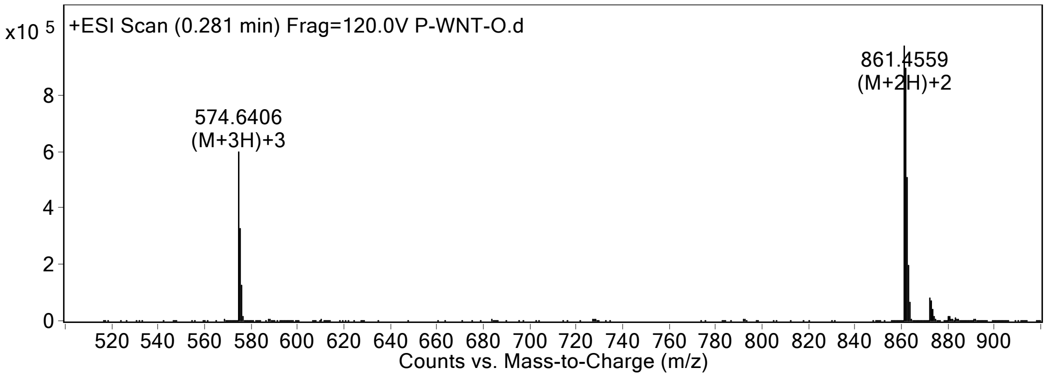

HPLC of compound Axin (469-482)

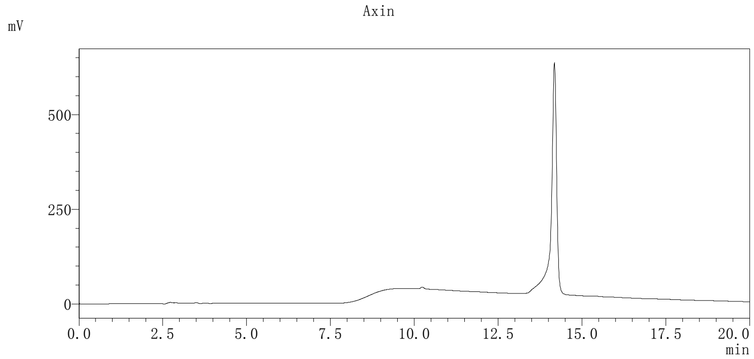

HR-Q-TOF-MS of compound WNT-1a

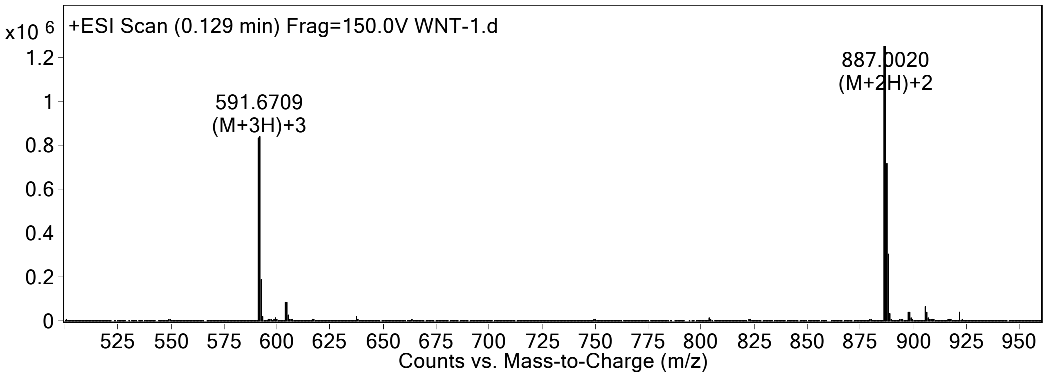

HPLC of compound WNT-1a

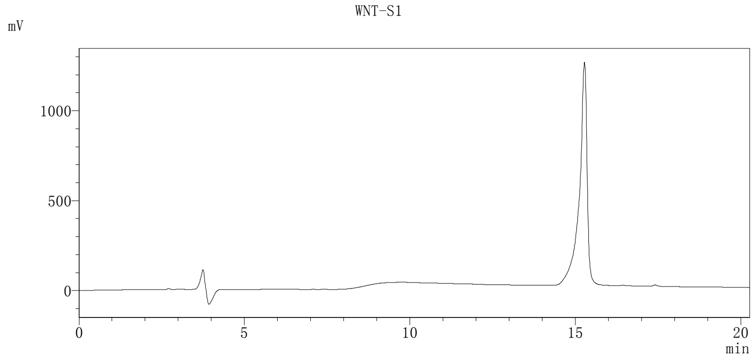

HR-Q-TOF-MS of compound WNT-2d

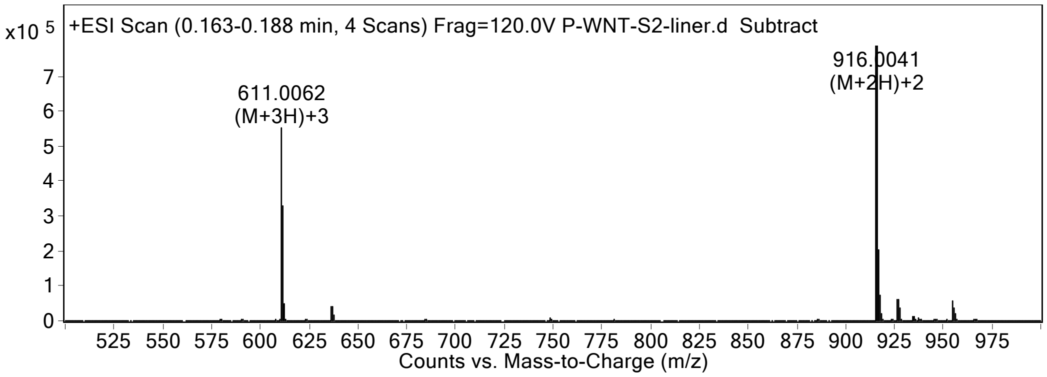

HPLC of compound WNT-2d

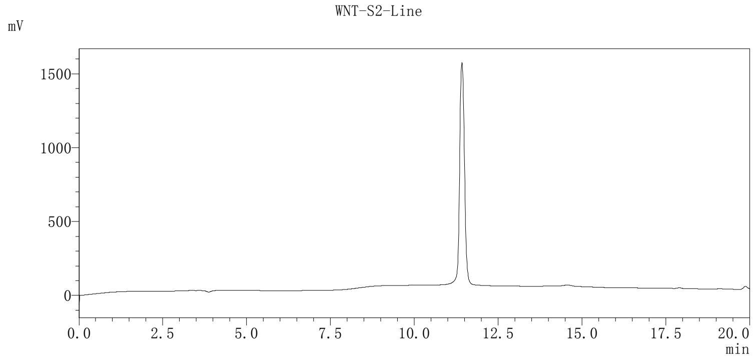

HR-Q-TOF-MS of compound WNT-3a

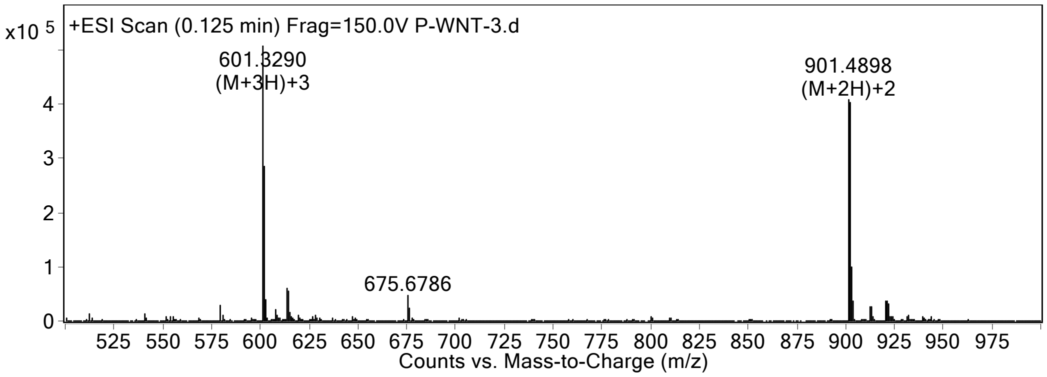

HPLC of compound WNT-3a

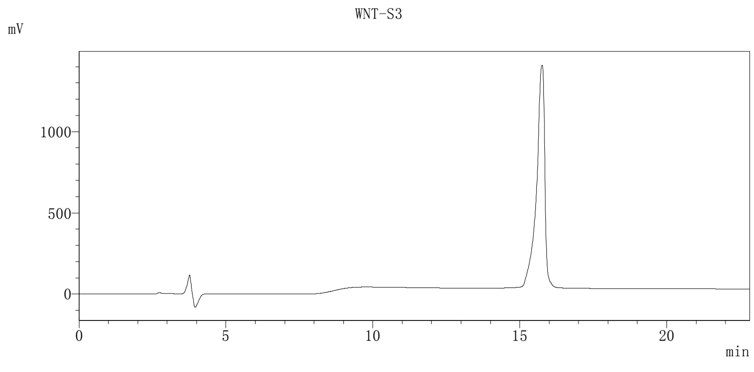

HR-Q-TOF-MS of compound WNT-3b

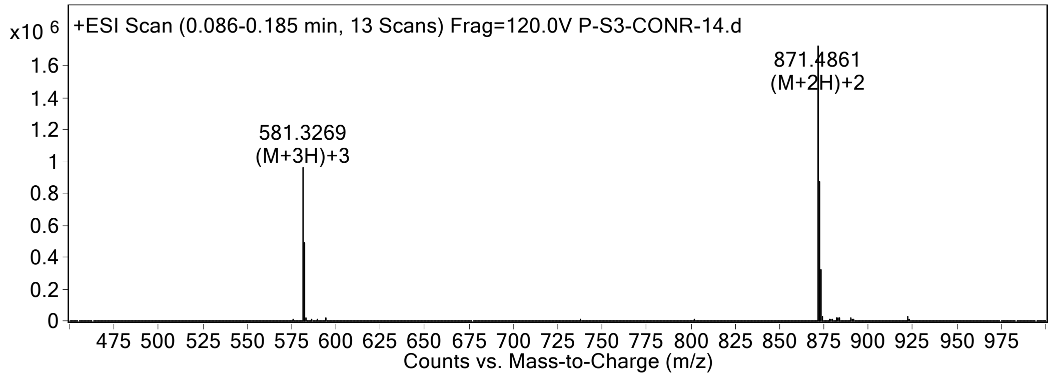

HPLC of compound WNT-3b

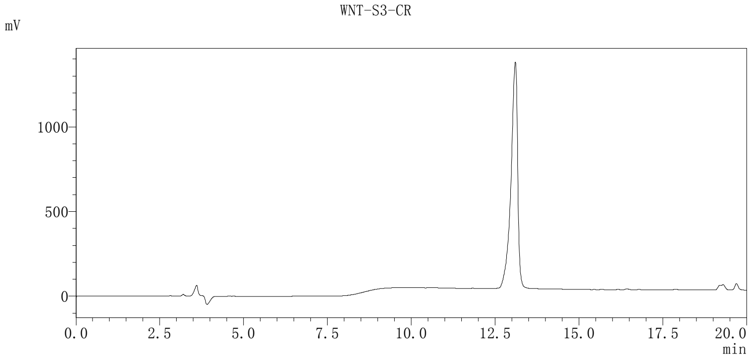

HR-Q-TOF-MS of compound WNT-3c

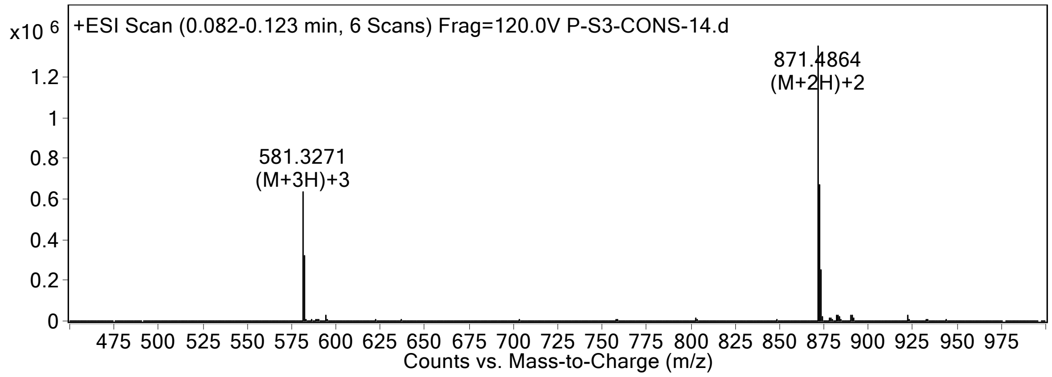

HPLC of compound WNT-3c

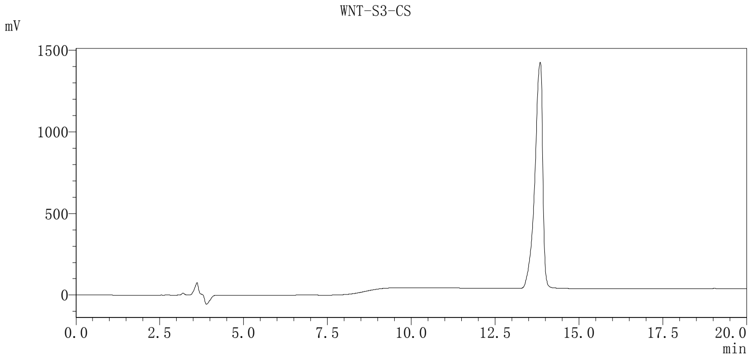

HR-Q-TOF-MS of compound WNT-3d

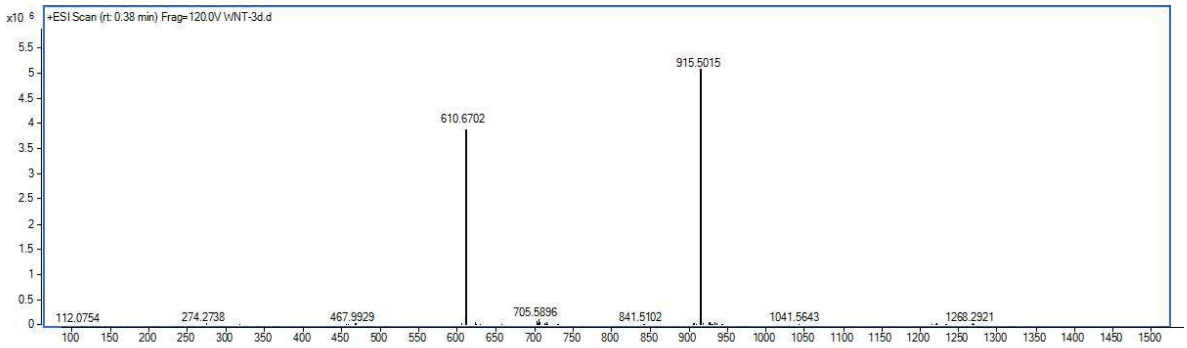

HPLC of compound WNT-3d

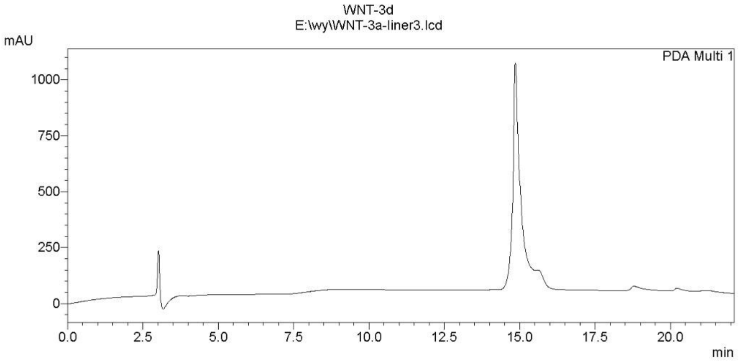

HR-Q-TOF-MS of compound WNT-4a

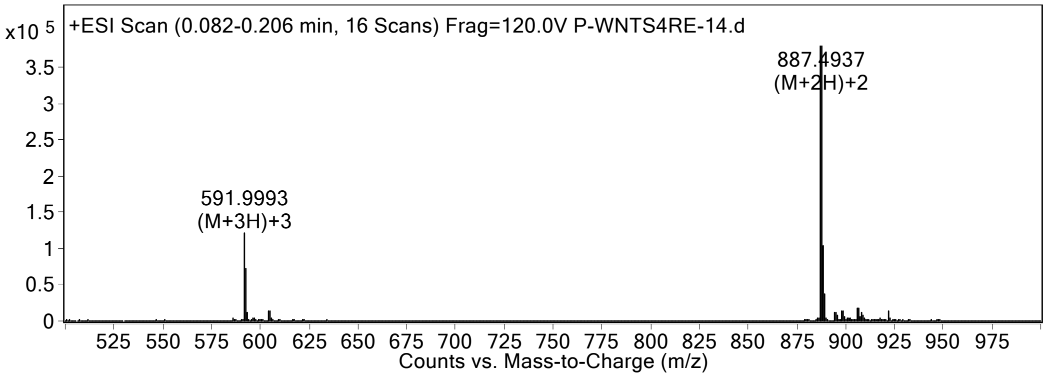

HPLC of compound WNT-4a

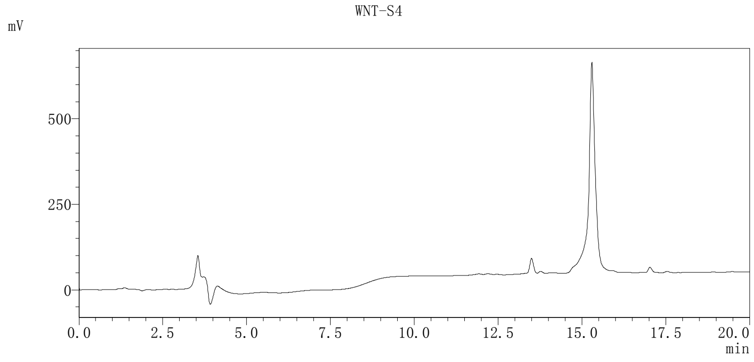

HR-Q-TOF-MS of compound WNT-5a

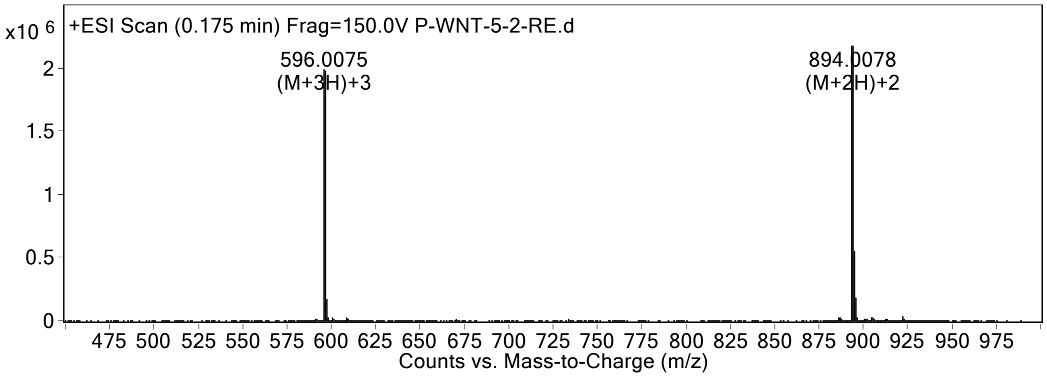

HPLC of compound WNT-5a

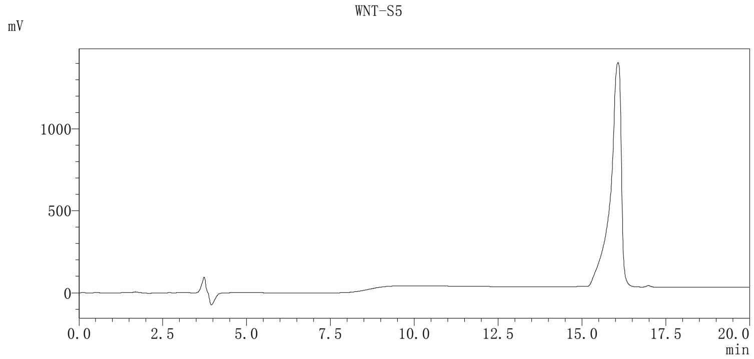

HR-Q-TOF-MS of compound WNT-5b

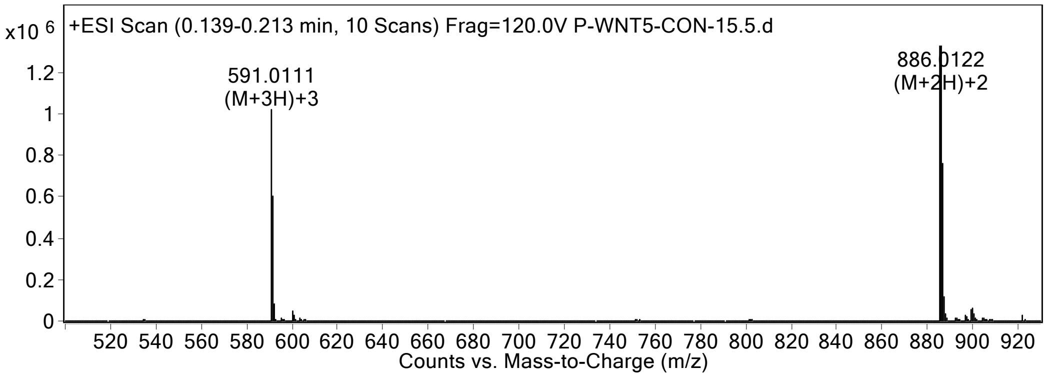

HPLC of compound WNT-5b

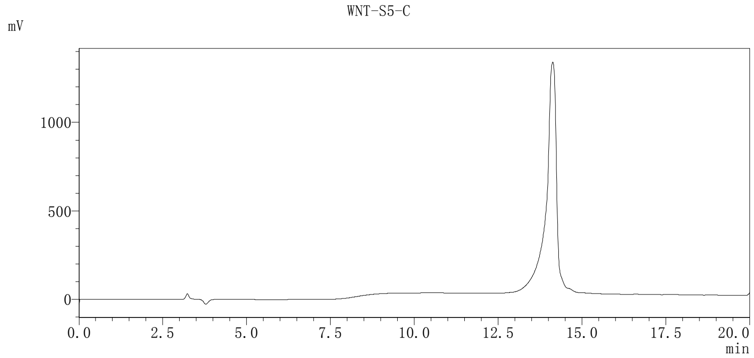

HR-Q-TOF-MS of compound WNT-6a

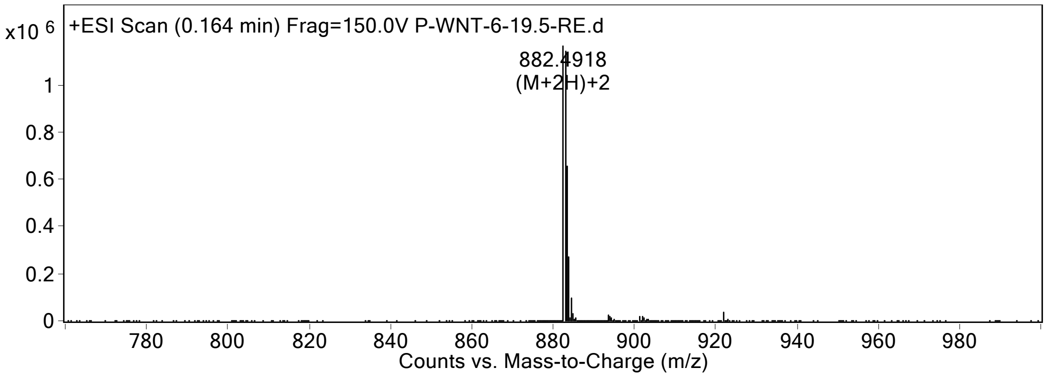

HPLC of compound WNT-6a

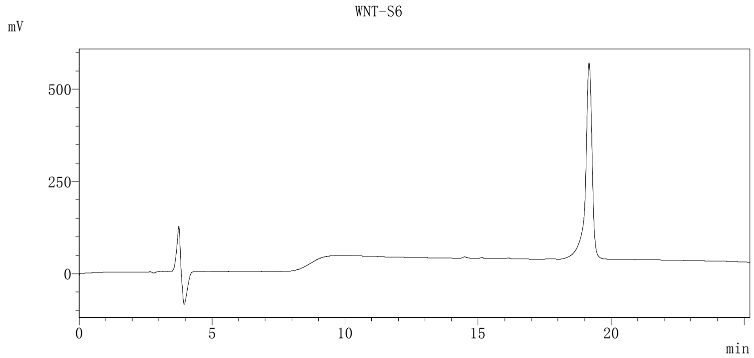

HR-Q-TOF-MS of compound WNT-6b

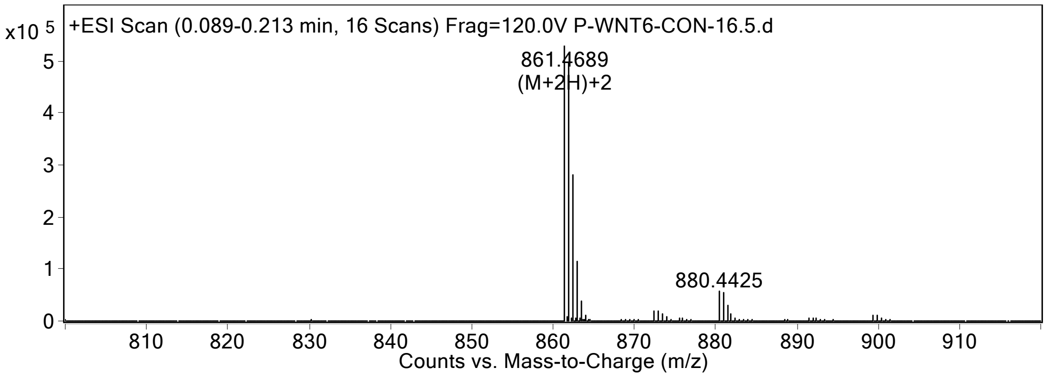

HPLC of compound WNT-6b

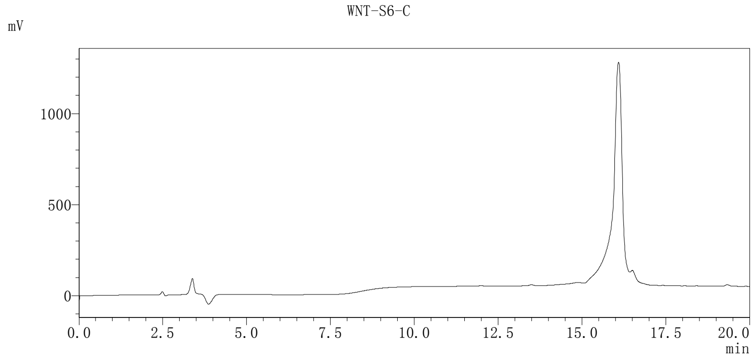

HR-Q-TOF-MS of compound WNT-7d

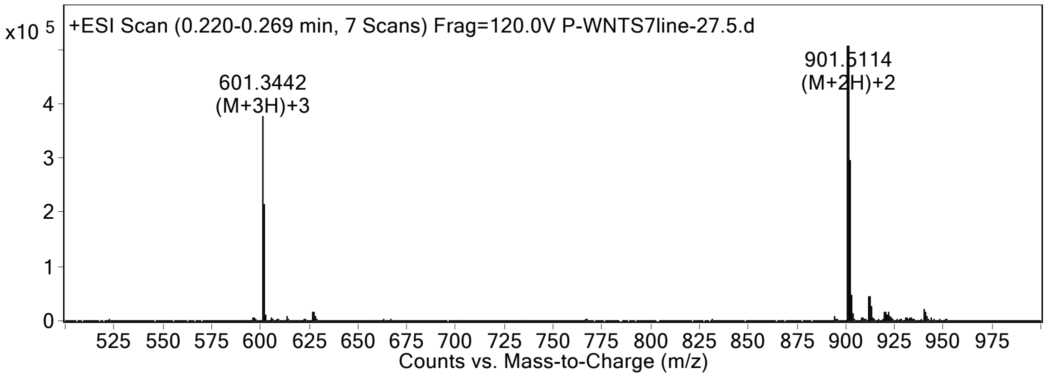

HPLC of compound WNT-7d

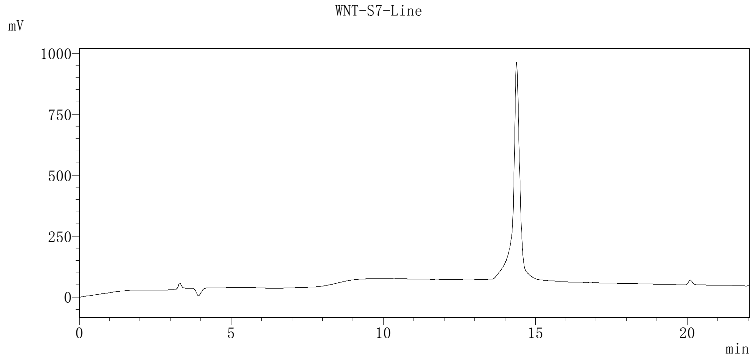

HR-Q-TOF-MS of compound WNT-8a

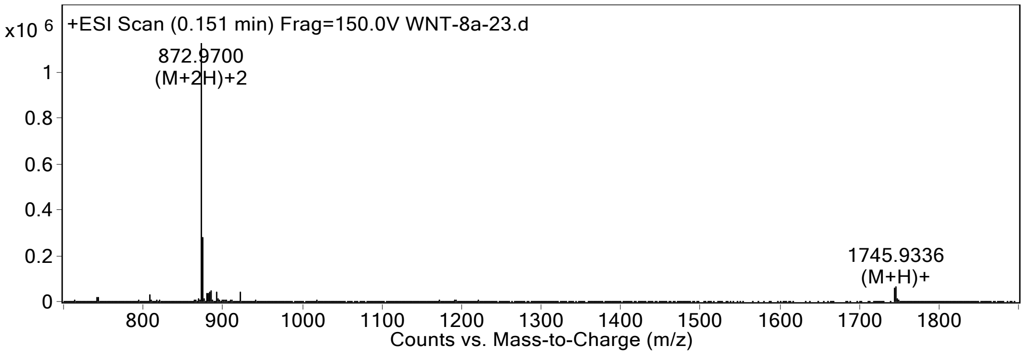

HPLC of compound WNT-8a

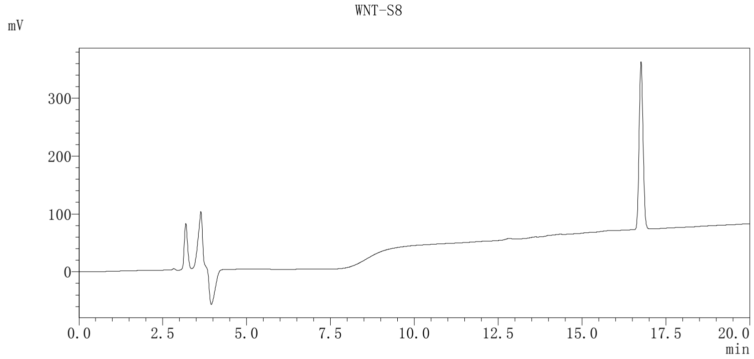

HR-Q-TOF-MS of compound FITC-β-Ala-Axin (469-482)

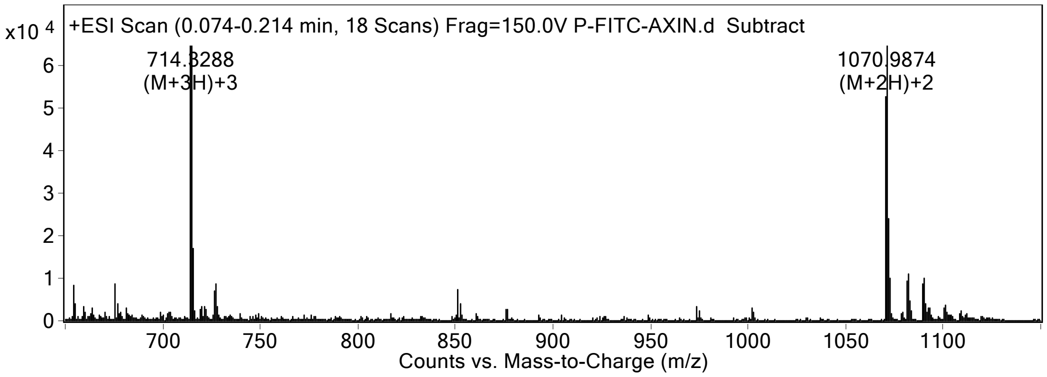

HPLC of compound FITC-β-Ala-Axin (469-482)

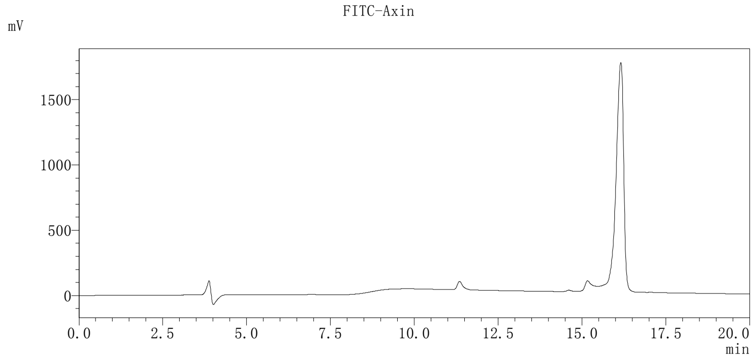

HR-Q-TOF-MS of compound FITC-β-Ala-WNT-3a

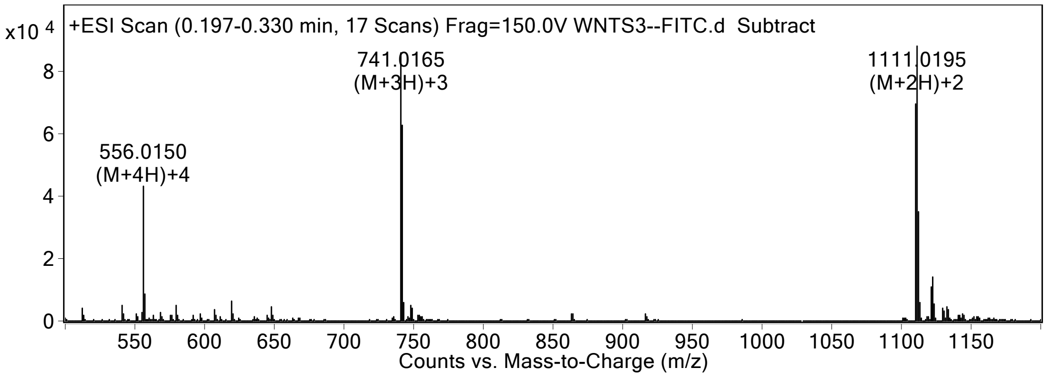

HPLC of compound FITC-β-Ala-WNT-3a

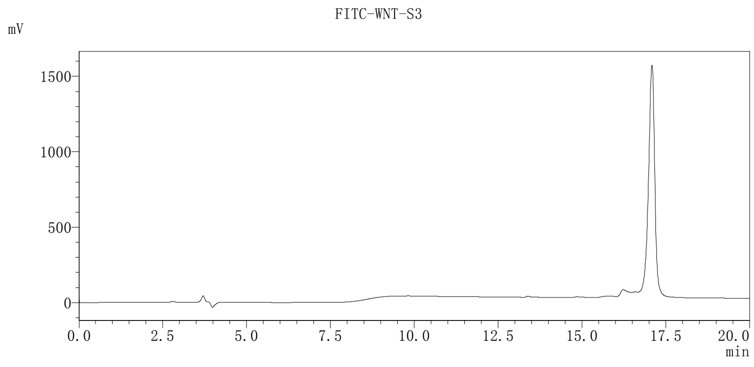

HR-Q-TOF-MS of compound FITC-β-Ala-WNT-3c

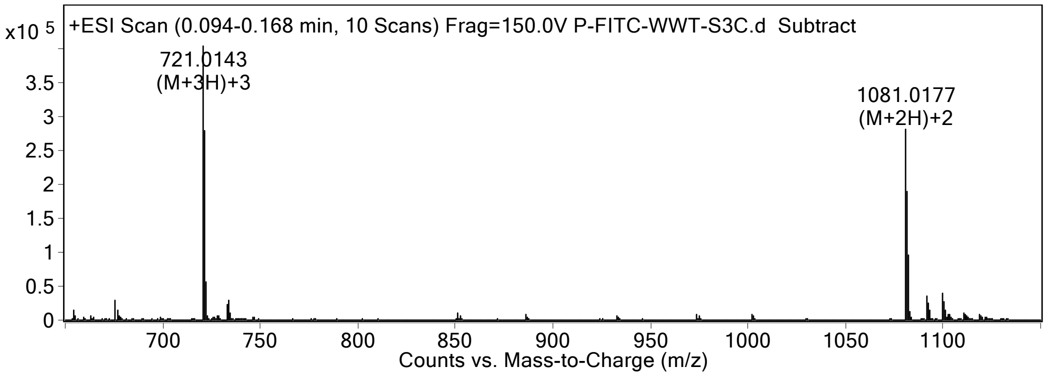

HPLC of compound FITC-β-Ala-WNT-3c

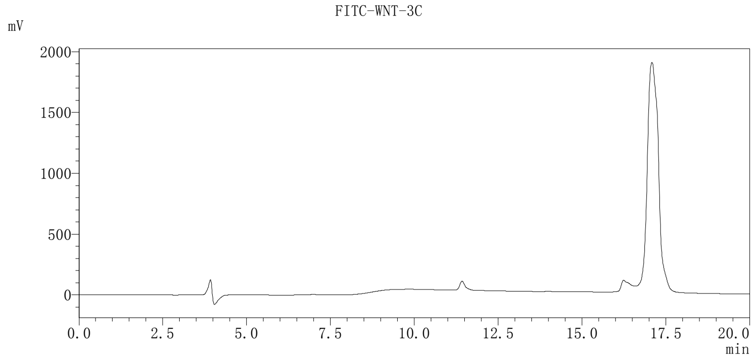

ESI-MS of compound WNT-3d-1

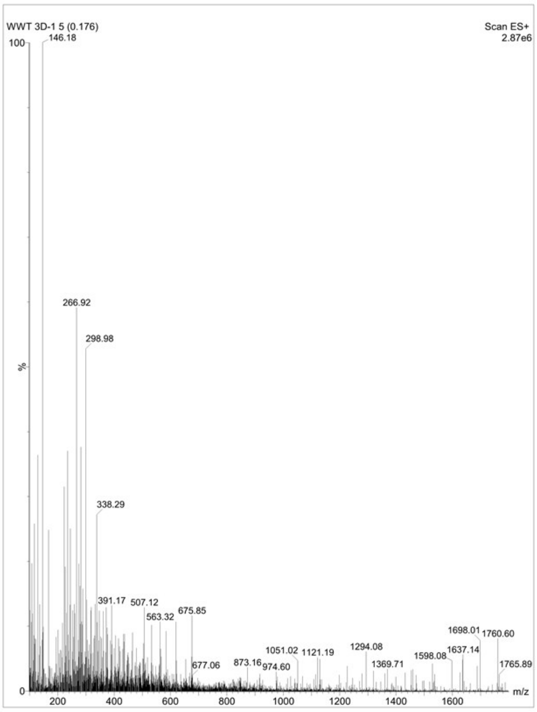

ESI-MS of compound WNT-3d-2

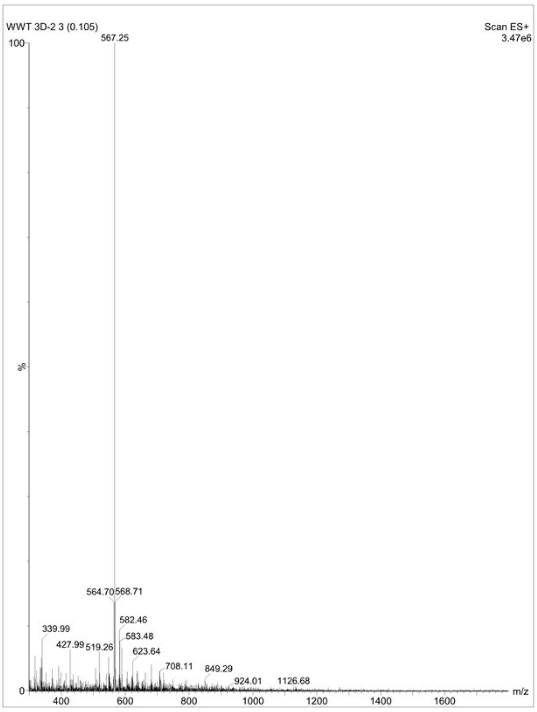

ESI-MS of compound WNT-3d-3

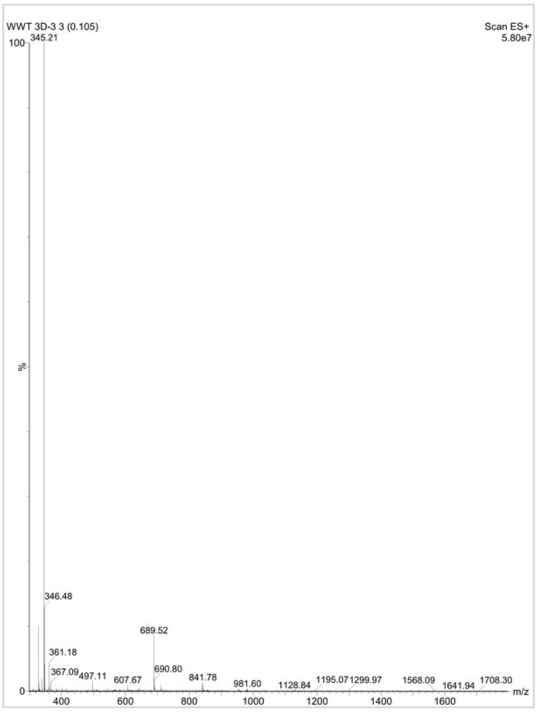

ESI-MS of compound WNT-3d-4

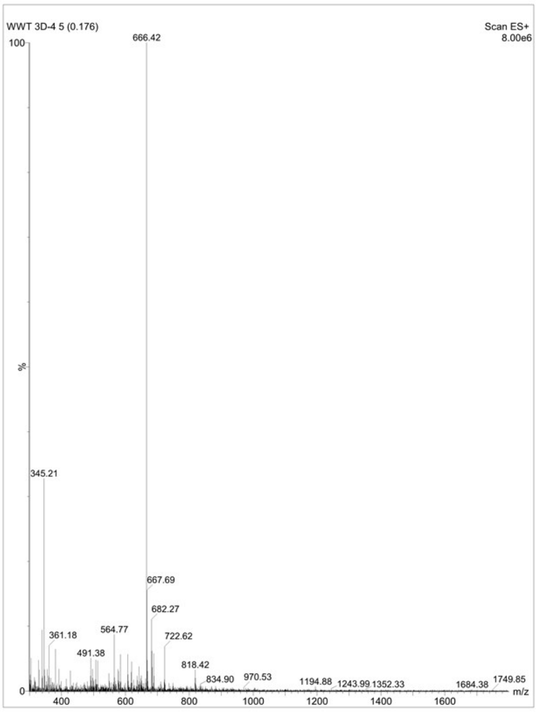

7. References:

1 Y. W. Kim, T. N. Grossmann and G. L. Verdine, *Nat. Protoc.*, 2011, **6**, 761-771.

2 V. Korinek, N. Barker, P. J. Morin, D. van Wichen, R. de Weger, K. W. Kinzler, B. Vogelstein and H. Clevers, *Science*, 1997, **275**, 1784-1787.

3 H. K. Cui, J. Qing, Y. Guo, Y. J. Wang, L. J. Cui, T. H. He, L. Zhang and L. Liu, *Bioorg. Med. Chem.*, 2013, **21**, 3547-3554.
